# Elucidating the Stereodirecting Effect of C‐4 Acyl Groups on Galactosyl Donors

**DOI:** 10.1002/anie.202521698

**Published:** 2026-02-23

**Authors:** Floor ter Braak, Frank F. J. de Kleijne, Teun van Wieringen, Peter H. Moons, Jonathan Martens, Jos Oomens, Peter A. Korevaar, Paul B. White, Thomas J. Boltje

**Affiliations:** ^1^ Institute for Molecules and Materials Radboud University Nijmegen The Netherlands; ^2^ HFML‐FELIX HFML‐FELIX Nijmegen The Netherlands

**Keywords:** galactosylation, neighboring group participation, reaction mechanisms, nuclear magnetic resonance, density functional theory

## Abstract

The stereoselective synthesis of 1,2‐*cis* glycosidic bonds is a significant challenge in carbohydrate chemistry. 1,2‐*Cis* galactosides can be obtained by using C‐4 acyl protection groups as a stereodirecting group, yet the underlying mechanism that steers selective galactosylation has remained elusive. Herein, we investigate the stereodirecting effect of C‐4 acyl groups in galactosides using glycosylation reactions, exchange NMR spectroscopy and DFT‐calculations. We found no experimental evidence for C‐4 dioxepanium ion formation through C‐4 acyl neighboring group participation. Instead, β‐glycosyl triflates were detected using exchange NMR which could afford α‐galactosides *via* S_N_2‐like displacement. Computational studies of the product‐forming transition state geometries reveal that the C‐4 benzoate group shields the β‐face of the galactosyl donor, thereby disfavoring reactions that proceed *via* the α‐glycosyl triflate pathway. These findings can explain the stereodirecting effect of C‐4 acyl groups on galactosyl donors, and provides fundamental insights for the development of stereoselective glycosylation methods in the future.

## Graphical abstract

Tipping the balance of the reaction coordinate by changing a single protecting group. We demonstrate that α‐ and β‐galactosyl triflates are formed during galactosylation reactions and likely glycosylate according to a Curtin–Hammett scenario. C‐4 acyl groups exert their stereodirecting effect by sterically blocking displacement of α‐galactosyl triflates thus providing α‐galactosides *via* the displacement of β‐galactosyl triflates.

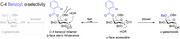

## 1 Introduction

Stereoselective glycosylation methods are crucial in the chemical synthesis of complex oligosaccharides [1, 2]. In order to form the glycosidic bonds that link the monosaccharide building blocks, a glycosyl donor carrying an anomeric leaving group is typically activated using a chemical promotor to afford *quasi*‐stable reaction intermediates. These are subsequently substituted by a nucleophilic glycosyl acceptor *via* a unimolecular or bimolecular reaction mechanism, thereby yielding a glycosidic bond [3]. The stereochemical outcome of this reaction is determined by nucleophilic attack on either the α‐ or β‐face of the reaction intermediate, leading to the respective glycoside products. The structure and reactivity of the reaction intermediates formed upon activation along with the nucleophile used are key in dictating the stereochemical outcome of the reaction [3, 4, 5, 6]. Glycosylation reactions pathways proceeding *via* a bimolecular [7] or unimolecular [8, 9, 10, 11, 12, 13] mechanism have been extensively studied. Notably, obtaining facial selectivity in the nucleophilic displacement step can be achieved using various types of neighboring group participation (NGP) by acyl groups on the glycosyl donor. For example, C‐2 acyl NGP affords a transient dioxolanium ion that shields the 1,2‐*cis*‐face, concurrently directing the nucleophile towards approach from the 1,2‐*trans*‐face. The mechanism of C‐2 NGP is supported by a robust body of evidence such as the isolation of crystalline dioxolanium ions [14], spectroscopic observations [15], theoretical studies [16], reaction concentration effects [17], and the rate acceleration observed for glycosyl donors containing a 1,2‐*trans* leaving group [14, 18]. Extension of this strategy to acyl groups at more distant positions (C‐3, C‐4, and C‐6) has frequently been proposed as a means to create the more challenging 1,2‐*cis* linkages [19, 20, 21, 22, 23, 24, 25, 26]. NGP of C‐3, C‐4, and C‐6 acyl protecting groups has long been a heavily debated topic [3, 25, 26, 27, 28, 29], and we have recently demonstrated that C‐3 acyl protecting groups on manno‐type sugars, such as mannosyl [30, 31], mannuronosyl [32], and rhamnosyl donors [33], can engage in C‐3 NGP, thereby forming a six‐membered dioxanium ion that directs the nucleophilic attack from the α‐face leading to α‐products. C‐4 acyl groups positioned on galactosyl donors are well known for their stereodirecting ability to afford α‐selectivity [34, 35, 36, 37, 38, 39, 40, 41, 42, 43, 44, 45, 46, 47, 48, 49, 50, 51, 52]. Early studies by Boons and Nifantiev showed that α‐selectivity was enhanced by the introduction of a C‐4 acyl group on galactosyl [34] and fucosyl donors [44, 45, 46, 47]. Interestingly, the observed α‐selectivity increased with more electron rich C‐4 acyl groups. Even though this is indicative of C‐4 NGP, conclusive mechanistic evidence supporting this hypothesis is lacking. C‐4 acyl NGP would afford a seven‐membered galactosyl dioxepanium ion, whose highly‐reactive nature complicates its experimental observation. We and others have shown that the use of infrared ion spectroscopy enables the characterization of galactosyl C‐4 dioxepanium ions in the gas‐phase [53, 54, 55]. In addition, feasibility of C‐4 NGP in solution‐phase conditions has been investigated using various acyl surrogates that could yield a stable bicyclic product upon galactosyl donor activation. The use of a C‐4 *tert*‐butyloxycarbonyl group was expected to afford a cyclic carbonate *via* the loss of isobutene upon NGP, but no evidence for such product was found [37, 56]. The use of a more nucleophilic C‐4 trichloroacetimidate (TCA) group did afford a stable oxazepine product, thus demonstrating that C‐4 NGP is geometrically feasible in this case [38, 39]. Contrasting research has shown that the ground‐state conformation of the C‐4 acyl group directs the carbonyl group away from the anomeric center, thereby disfavoring NGP [38]. The barrier towards a conformer that enables NGP was lowered by the introduction of an equatorial C‐4 methyl group, which converted the secondary ester into a conformationally mobile tertiary ester. Low‐temperature NMR studies did reveal the formation of the galactosyl dioxepanium ion for the tertiary ester, whereas no such bridged ion was observed for the secondary ester under similar conditions. It must be noted that the tertiary ester set did not galactosylate with significantly higher α‐selectivity, suggesting that the observed C‐4 dioxepanium ion is not the stereodirecting reaction intermediate [37, 38]. Furthermore, under superacid conditions the C‐4 acyl was not found to engage in NGP [57] and recent studies by Jensen [58], McGarrigle [59], and Nifantiev [60] also indicate that C‐4 NGP is not operative and suggest that β‐galactosyl triflates are in fact the reactive intermediates responsible for α‐galactosylation. Despite these studies, conclusive experimental evidence for the existence of β‐galactosyl triflates is lacking and the origin of the C‐4 acyl group stereodirecting remains ambiguous.

Herein, we report on the use of exchange NMR techniques to investigate the reaction intermediates that form in glycosylations with C‐4 acyl modified galactosyl donors. No evidence for the formation of galactosyl dioxepanium ions was found. Instead, β‐galactosyl triflate intermediates were detected to equilibrate with the α‐galactosyl triflate. Since an S_N_2‐like displacement of a β‐galactosyl triflate intermediate could afford α‐galactosides, we investigated how their stability and reactivity was affected by the C‐4 substituent. Exchange NMR experiments illustrated that the C‐4 benzyl and C‐4 acyl substituted β‐galactosyl triflates exhibit similar exchange kinetics. Next, we modeled the transition state geometries and energies of the product forming steps using high level computations. These calculations suggest that glycosylation of both the C‐4 acyl and C‐4 ether substituted donors are under Curtin‐Hammett control. This means that the glycosyl triflate interconversion is faster then product formation and hence that the product distribution is dependent on the difference in product‐forming rates from the α‐ and β‐galactosyl triflate reaction intermediates. Calculations suggest that in galactosylations with a C‐4 acyl group, β‐galactoside formation was disfavored by the C‐4 acyl group as it adopted a conformation that sterically blocks the β‐face. In contrast, the more mobile nature of the C‐4 ether substituent did not hinder the approach of the nucleophile from the β‐face in this case. In both cases, approach from the α‐face of the β‐galactosyl triflate was possible and neither was affected by the type of C‐4 protecting group. The C‐4 acyl group therefore tips the balance of the Curtin–Hammett scenario towards α‐selectivity by sterically disfavoring nucleophilic attack from the β‐face. Hence, this study provides novel insights in the mechanism of C‐4 acyl stereodirection and may extend to other glycosyl donors containing an axial C‐4 acyl substituent such a fucosides.

## 2 Results and Discussion

**FIGURE 1:**
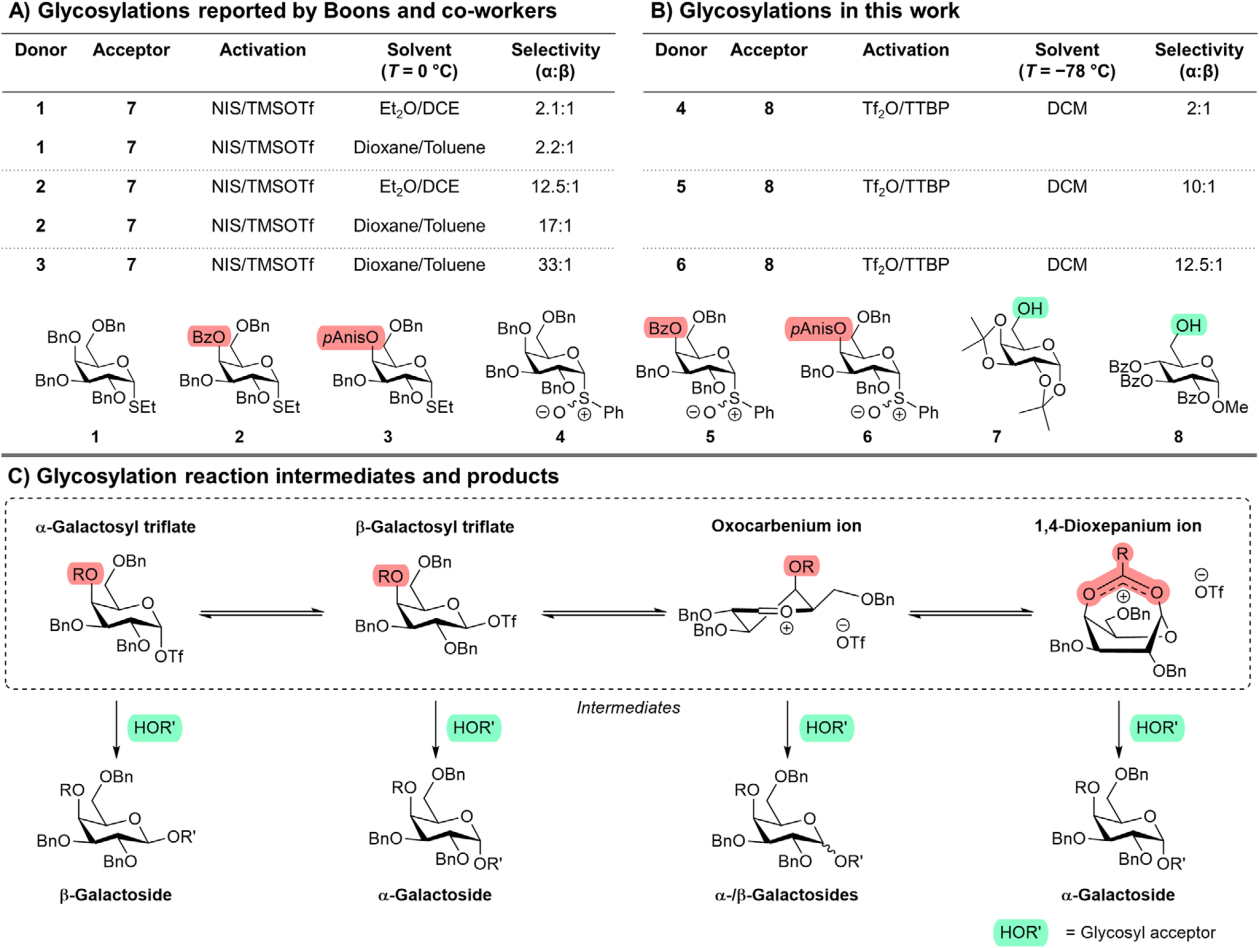
(A) Glycosylation outcomes reported by Boons et al; (B) Glycosylation under preactivation conditions for donors **4**–**6** used in this work. α/β‐selectivity was determined using quantitative HSQC [62, 63]; (C) Glycosylation intermediates and products of galactosylation.

Boons and coworkers studied the influence of C‐4 substitution on stereoselective galactosylations in various solvent systems (Figure 1A). Perbenzylated donor **1** glycosylated with a modest selectivity irrespective of solvent choice. In contrast, donors bearing C‐4 benzoate groups (**2** and **3**) exhibited much improved α‐selectivity. The best selectivity was observed for *p*‐anisoylated donor **3** using a 1,4‐dioxane/toluene solvent system. As this type of solvent system is known to improve α‐selectivity irrespective of the glycosyl donor protecting group pattern, the contribution of the solvent system and C‐4 acyl group could not be dissected [34, 61].

**FIGURE 2:**
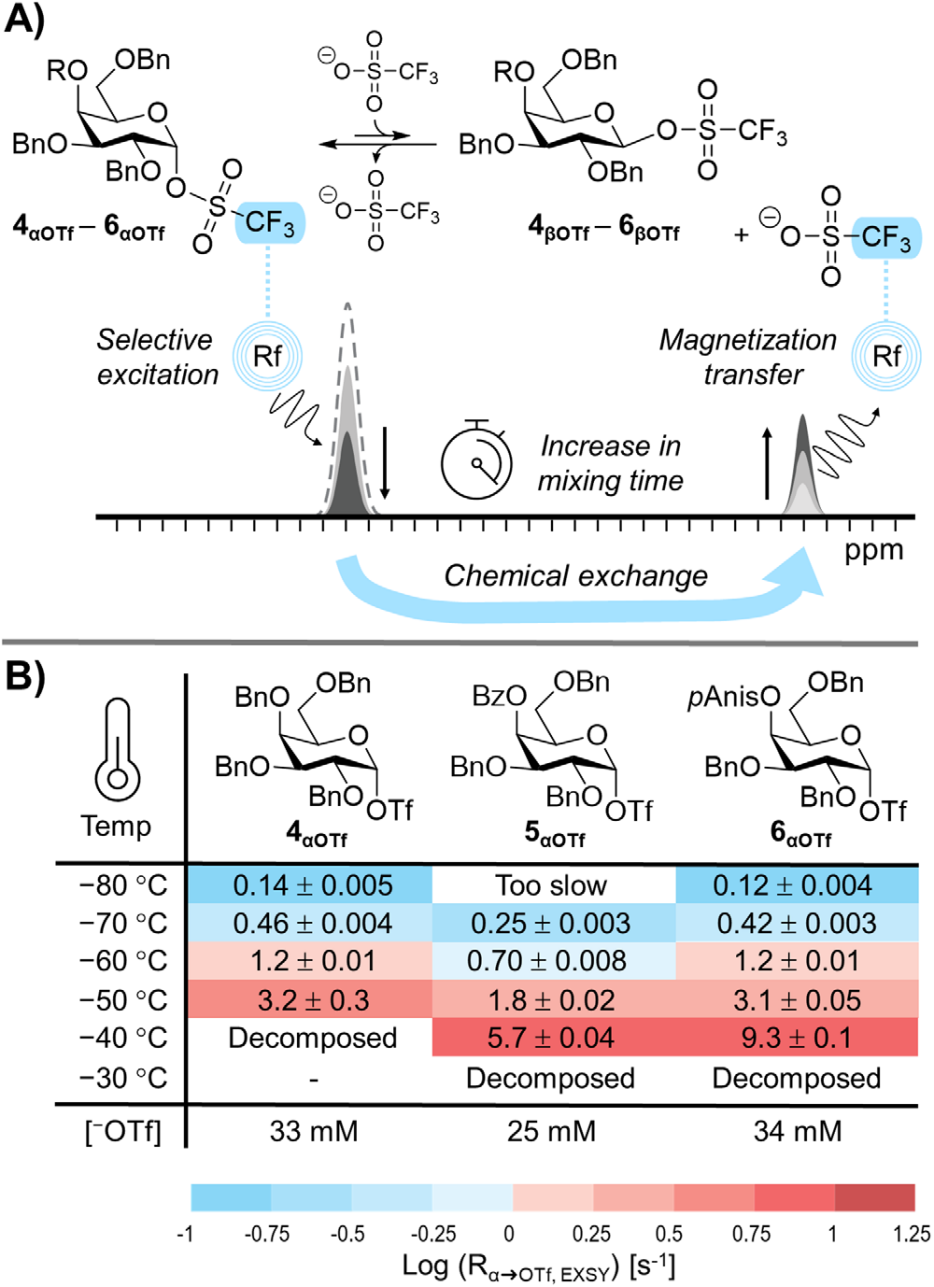
^19^F EXSY experiments of galactosides **4** – **6**. (A) Illustration of ^19^F EXSY NMR experiments on galactosyl triflates; (B) Triflate dissociation kinetics of α‐galactosyl triflates **4**_αOTf_ – **6**_αOTf_ at different temperatures.

Therefore, we repeated the glycosylation reactions under pre‐activation conditions in dichloromethane (DCM), a non‐directing solvent commonly used in glycosylation reactions (Figures S3–S5). Perbenzylated donor **4** was chosen as a benchmark, while the benzoylated and *p*‐anisoylated donors (**5** and **6**) were selected in order to explore the impact of C‐4 acyl groups on the glycosylation selectivity. Our results revealed trends similar to those observed by Boons and co‐workers, with benzylated donors exhibiting poor α/β selectivity and galactosylations with C‐4 acyl donors **5** and **6** showed significantly improved α‐stereoselectivity (Figure 1B). Interestingly, the *p*‐anisoyl group did not provide a significant increase in α‐selectivity compared to the benzoylated derivative. Nevertheless, addition of a C‐4 acyl group instead of a C‐4 benzyl ether clearly led to the emergence of α‐selectivity, consistent with earlier reports [34, 36, 37, 38, 58]. The two most commonly proposed reaction intermediates that could lead to the observed α‐selectivity are the C‐4 dioxepanium ion formed by C‐4 NGP, and β‐galactosyl triflates. In order to investigate the existence of these unstable and low‐abundance intermediates, exchange NMR experiments were conducted. To this end, galactosyl donors **4–6** were activated at −80°C using triflic anhydride (Tf_2_O) in the presence of the non‐nucleophilic base 2,4,6‐tri‐*tert*‐butyl‐pyrimidine (TTBP), which yielded the α‐galactosly‐triflate as the main observed reaction intermediate (Figures S6–S8) [64]. However, this intermediate cannot explain the predominant formation of α‐galactosides *via* a traditional S_N_2‐like displacement. Instead, a rapid chemical equilibrium could exist between the *quasi*‐stable α‐galactosyl triflate and the β‐galactosyl triflate or a 1,4‐bridged dioxepanium ion (Figure 2A). Both processes result in the displacement of the anomeric triflate group of the α‐glycosyl triflate, and both highly‐reactive intermediates could afford α‐glycosides upon reacting with a glycosyl acceptor. We previously demonstrated that the triflate dissociation rate and mechanism of dissociation can be investigated using ^19^F exchange spectroscopy (EXSY) NMR [31, 32]. Accordingly, we investigated the α‐galactosyl triflate dissociation kinetics (R_α → OTf, EXSY_) at different temperatures using the perbenzylated α‐galactosyl triflate **4** as a benchmark (Figure 2B). Moderate differences in triflate dissociation were observed for the perbenzylated **4**_αOTf_ and its C‐4 benzoylated analogue **5**_αOTf_ (Figure 2B). Whilst acylation would be expected to disarm the galactosyl donor, as observed for gluc(uron)osyl α‐triflate dissociation [32], the differences observed here were caused by the slight difference in ^−^OTf concentration between experiments. Under identical conditions, the galactosyl triflates **4_αOTf_**‐**6_αOTf_** therefore likely released triflate at comparable rates, suggesting that the stability and exchange kinetics of the galactosyl triflates does not directly explain the observed selectivity difference. Moreover, we have previously established that C‐3 NGP in mannosides result into the formation of dioxanium ions [30, 31, 32]. Notably, the C‐3 acyl mannosyl donors showed a much faster α‐glycosyl triflate dissociation rate compared to the C‐3 ether benchmark, as a result of C‐3 NGP. Such an increase in α‐galactosyl triflate dissociation was not observed for C‐4 acylated galactosyl triflates **5**_αOTf_ and **6**_αOTf,_ which suggests that C‐4 NGP does not play a major role in the dissociation of the anomeric triflate in these cases.

**FIGURE 3:**
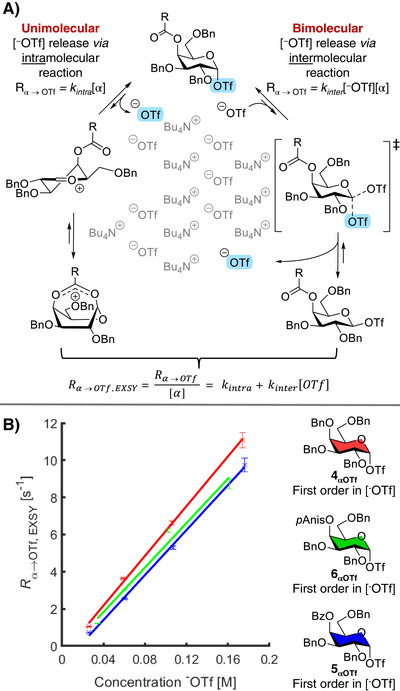
Mechanism of triflate dissociation. (A) Theoretical unimolecular and bimolecular mechanisms of triflate dissociation; (B) Triflate dissociation kinetics of α‐galactosyl triflates **4_α_** – **6_α_** at −60°C, as a function of triflate concentration demonstrates a first‐order triflate dependence in all studied galactosyl donors.

To further investigate the formation of either the equatorial β‐galactosyl triflate or the 1,4‐dioxepanium ion, we conducted the same ^19^F EXSY experiments as a function of the ^−^OTf concentration to investigate the mechanism of galactosyl triflate dissociation. The formation of the β‐galactosyl triflate is expected to be a bimolecular process and, hence, its rate should be first order with respect to the ^−^OTf concentration (Figure 3A). In contrast, C‐4 NGP represents a unimolecular pathway with a rate independent of the triflate anion concentration. Therefore, analyzing the triflate dissociation rate at varying ^−^OTf concentrations provides insights into the underlying mechanism of galactosyl triflate dissociation. Hence, α‐galactosyl triflate dissociation rates were measured at increasing ^−^OTf concentrations by the stepwise addition of tetrabutyl ammonium triflate (Figure 3B). All galactosyl donors showed an increase in R_α→OTf, EXSY_ at increasing [^−^OTf] according to bimolecular kinetics (Figure 3B). Notably, if the reaction solely proceeds *via* a bimolecular mechanism, then R_α→OTf, EXSY_ should theoretically be zero when [^−^OTf] = 0, and in that case C‐4 NGP is not operative. Indeed, for all donors, extrapolation of R_α→OTf, EXSY_ intercepted close to the origin, at which both the [^–^OTf] and R_α→OTf, EXSY_ are nearing zero. Hence, the galactosyl donors likely undergo a S_N_2‐like triflate displacement, yielding the β‐galactosyl triflates irrespective of their C‐4 protecting group.

The ^19^F EXSY experiments suggested that C‐4 acyl NGP does not take place in case of donors **5** and **6** in the solution‐phase. We have previously shown that reducing the steric bulk of the non‐participating protecting groups boosts populations of dioxanium ions that result from C‐3 acyl NGP in mannosides [30, 31]. Additionally, the introduction of a more electron rich *p*‐anisoyl ester improved dioxanium ion stability in C‐3 acyl NGP [31]. Therefore, we investigated the 2,3,6‐tri‐*O*‐methyl‐4‐*O*‐(*p*‐anisoyl‐^13^C)‐galactosyl donor **7**, which combines both properties into the most ideal galactosyl donor to investigate C‐4 NGP. Using ^13^C chemical exchange saturation transfer (CEST) NMR, we scanned for a low‐abundant 1,4‐dioxepanium ion or β‐triflate intermediate. First, galactosyl donor **7** was activated using diphenyl sulfoxide (Ph_2_SO) and Tf_2_O [65] in the absence of TTBP [30]. Next, ^13^C CEST NMR was employed to scan frequencies between 190 to 160 ppm. The ^13^C CEST profile was obtained by plotting the relative intensity of a selected resonance, the ^13^C labeled carbonyl carbon of the anisoyl group, against the degree of saturation transfer to this peak while scanning the saturation offset frequency (190 to 160 ppm). This did not provide evidence for the formation of the dioxepanium ion at −80°C (Figures S9 and S13–S15). Instead, evidence was observed for the equatorial β‐triflate, based on ^1^H and ^19^F CEST NMR (Figure S14) and ^19^F EXSY (Figure S15). Hence, despite optimizing all parameters to favor the formation and detection of the C‐4 dioxepanium ion, no such species could be detected. This could be because the kinetics of dioxepanium ion formation falls outside of the exchange NMR detection window but it should be noted that the β‐galactosyl triflate was detected under the same conditions.

**FIGURE 4:**
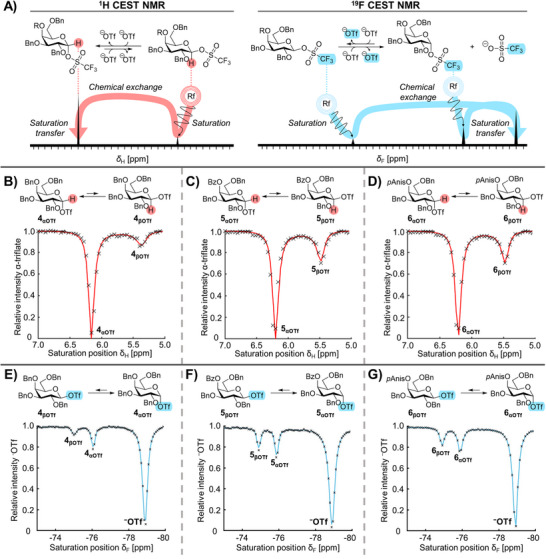
CEST profiles of activated galactosyl donors **4** – **6** (A) Graphical visualization of ^1^H and ^19^F CEST NMR; (B) ^1^H CEST profile of **4**; (C) ^1^H CEST profile of **5**; (D) ^1^H CEST profile of **6**; (E) ^19^F CEST profile of **4**; (F) ^19^F CEST profile of **5**; (G) ^19^F CEST profile of **6**; All CEST spectra were recorded at −60°C.

As the EXSY and CEST experiments indicated that galactosyl triflate dissociation is caused by the formation of β‐galactosyl triflates instead of C‐4 acyl NGP, we set out to detect the β‐galactosyl triflates derived from galactosyl donors **4–6**. To this end, ^1^H and ^19^F CEST NMR were employed (Figure 4A). The ^1^H CEST profiles were obtained by plotting the relative intensity of a selected resonance, the H‐1 signal belonging to the α‐galactosyl triflate, against the degree of saturation transfer to this peak while scanning the saturation offset frequency. All galactosyl donors (**4** – **6**) exhibited clear evidence of upfield saturation transfer *via* ^1^H CEST NMR that could be attributed to β‐galactosyl triflate intermediates **4_βOTf_** – **6_βOTf_** (Figure 4B–D). No evidence for galactosyl dioxepanium ion formation was observed due to the absence of saturation transfer at the expected chemical shift (6.4 ppm – 6.8 ppm) [37]. To further validate the observation of β‐galactosyl triflates, we also performed ^19^F CEST experiments by monitoring the triflate CF_3_ resonance. Indeed, the ^19^F CEST NMR also indicated the presence of β‐galactosyl triflates **4** – **6_βOTf_** (Figure 4E–G). The assignment of the β‐galactosyl triflate was made based on the saturation transfer peak at δ_F_ ≈‐74.8 ppm. This value is similar to that of a β‐glucosyl triflate that was directly observed and characterized using ^1^H NMR [31].

To exclude that the species at δ_H_ ≈ 5.4 ppm corresponded to a reaction intermediate specific to the activation of glycosyl sulfoxides such as glycosyl sulfenates [66], we also investigated another donor and activation system. To this end, the corresponding perbenzylated thioglycoside donor was activated with Ph_2_SO and Tf_2_O in the presence of TTBP. This resulted in the formation of the α‐galactosyl triflate along with the galactosyl α‐oxosulfonium ion (Figures S10–S12) [67]. Subsequently, a ^1^H CEST profile was recorded taking the α‐galactosyl triflate H‐1 resonance as a reference and the ^1^H CEST profile again displayed saturation transfer from *δ*_H_ ≈ 5.4 ppm. This suggests that this resonance is not specific to the activation of glycosyl sulfoxides. Interestingly, the α‐galactosyl oxosulfonium ion did not lead to saturation transfer suggesting that if there is chemical exchange, this process is too slow and T_1_ relaxation occurs before magnetization can be transferred to the glycosyl triflate. Hence, these results indicate that β‐galactosyl triflate are formed *via* rapid chemical exchange with the more stable α‐galactosyl triflate during galactosylations. The chemical shifts correspond to those previously reported for similar reactive intermediates [31], and these observations are underlined by the first‐order dependence of α‐galactosyl triflate dissociation as a function of [^−^OTf] concentration (Figure 3).

**FIGURE 5:**
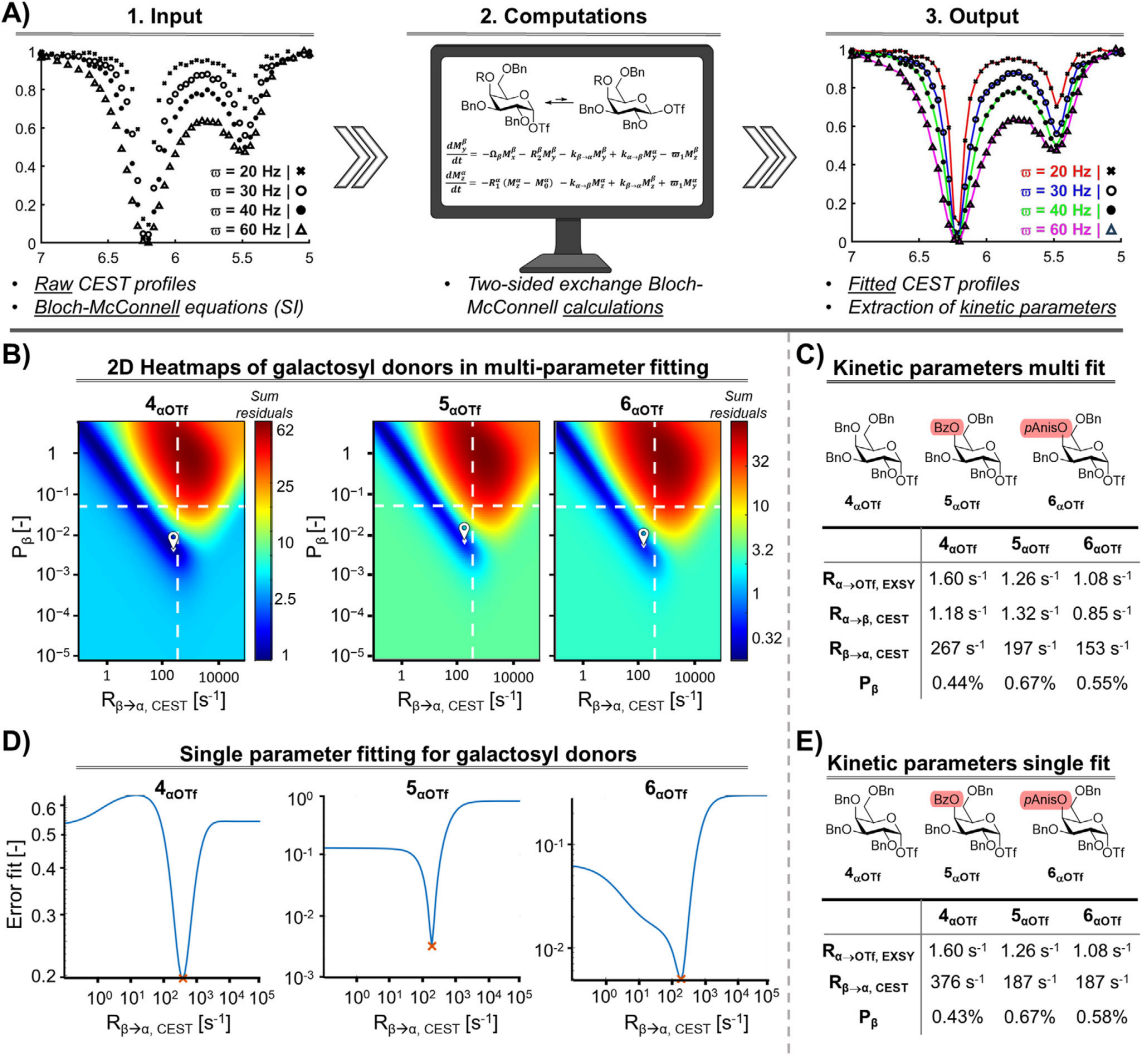
Graphical visualization of the CEST fitting procedure: experimental data to construct CEST profiles is fitted to a two‐site exchange model using the Bloch‐McConnell equations which results in fitted graphs from which the kinetic parameters can be extracted: (A) Using the Bloch‐McConnell equations the CEST profiles were simulated at varying values for the β‐triflate population and exchange rates; these simulations were compared to the input raw CEST profiles and the errors were plotted in the z‐dimension of the heatmap providing a best fit at the marker; (B) Heatmaps of R_β → α, CEST_ plotted against P_β_; (C) Summary of the kinetic parameters that were obtained from the best fitting with the rates found by ^19^F EXSY as reference. (D) Single parameter fitting experimental data. (E) Summary of the kinetic parameters that were obtained from the best single‐parameter fits.

Unfortunately, the low population of **4_βOTf_** – **6_βOTf_** at equilibrium prevented their direct characterization or quantification *via* 1D NMR. We reasoned that the β‐galactosyl triflate population could be quantified by fitting the Bloch‐McConnell equations to the obtained CEST profiles (Figure 5) [68]. The Bloch‐McConnell equations correlate the magnetization in nuclei to the exchange rate constants, concentrations of the exchanging species, and relaxation times (T_1_ and T_2_) [69, 70, 71]. CEST NMR can thus be used to determine the rate of α‐galactosyl triflate to β‐galactosyl triflate exchange and *vice versa*. First, the CEST profiles were fitted to the Bloch‐McConnell equations to determine the exchange rates.

To this end, CEST data recorded with a saturation field (ϖ) of 20 Hz was analyzed by utilizing a multiparameter fitting method based on the work by the labs of Gschwind [68], Sherry [71], and Bachert [72]. The two‐sided exchange model studied in this work provided exchange rates R_β → α, CEST_, R_α → β, CEST_, and hence allowed for the calculation of the β‐galactosyl triflate population (P_β_). Fitting of the α‐galactosyl triflate **4_αOTf_** data yielded R_β → α, CEST_ = 267 s^−1^ and a β‐galactosyl triflate population of 0.44%. Notably, multiple combinations of the exchange rates and β‐galactosyl triflate populations yielded satisfactory fits (Figure S16). Inspired by the work of Zaiss et al., [73] fitting methods were adapted that included multiple CEST profiles with saturation field strengths of 30, 40, and 60 Hz. Hence, CEST profiles recorded at different saturation field strengths were simultaneously fitted in order to improve accuracy (Figures S17–S19). As expected, this approach narrowed the number of combinations that provided accurate fits. However, a variety of combinations of R_β → α, CEST_ and P_β_ yielded fittings that matched the experimental data. These combinations were plotted against the sum of errors in a heatmap in order to provide an overview of possible solutions for R_β → α, CEST_ and P_β_ (Figure 5B). The individual fitting was considered within the area where the sum of errors was below 2.5, and the best fit was determined based on the best overall match with peak widths and height (Figures S20–S22). The best fits highlighted in the 3D plot were challenged by three experimentally validated boundary conditions to ascertain the outcome of the fitting. First, the exchange rate should be slower than the difference in chemical shift between the two species (R_β → α, CEST_ + R_α → β, CEST_ < Δϖ, vertical white dashed line in Figure 5B). Secondly, the population of the β‐galactosyl triflate should not exceed 5% (horizontal white dashed line in Figure 5B) as it would be detectable above this population in 1D ^19^F NMR despite exchange line‐broadening. Third, the rate of α‐galactosyl triflate to β‐galactosyl triflate exchange found by CEST should closely match the rate of triflate dissociation found by ^19^F EXSY NMR (R_α → β, CEST_ ≈ R_α → OTf, EXSY_). Indeed, the multi CEST profile fit approach that was based on the fitting method from the Gschwind lab provided fits that fulfilled these boundary conditions for donors **4** – **6** (Figure 5C). These results provided β‐galactosyl triflate populations of 0.44%, 0.67%, and 0.55% derived from C‐4 benzyl (**4**), benzoyl (**5**), and *p*‐anisoyl (**6**) protected donors, respectively. The exchange rates from the β‐galactosyl triflate to α‐galactosyl triflates (R_β → α, CEST_) were determined to be 267, 197, and 153 s^−1^ respectively. Critically, this fitting procedure required the α‐galactosyl triflate concentration, as well as the T_1_ and T_2_ relaxation time of both the α‐galactosyl and β‐galactosyl triflate as input. The population of the α‐galactosyl triflate was fixed (*M*_α_ = 1.0), the longitudinal relaxation time (T_1_) of the α‐galactosyl triflate anomeric proton was acquired using inversion recovery NMR and the transverse relaxation time (T_2_) of the α‐galactosyl triflate was determined using the Carr–Purcell–Meiboom–Gill sequence (CPMG) NMR experiment. Both the longitudinal and transverse relaxation of the corresponding β‐galactosyl triflate proton were assumed to be equal to the corresponding α‐galactosyl triflate, given their very similar molecular structure, as they could not be measured directly. To investigate that impact of the T_1_ and T_2_ on the quality of the obtained fit we altered the relaxation rates of the β‐galactosyl triflate by 20% and 50% relative to the measured relaxation of the α‐galactosyl triflate. This study was conducted on a single parameter fit where the measured EXSY rate was used as input and only the R_β → α, CEST_ was determined in the course of the fitting procedure (Figures S23–S25). The values found from the single parameter fit were in good agreement with those found for the multi‐parameter fit (Figure 5D). In the single parameter fit the relaxation rates of the β‐galactosyl triflate were manually varied but resulted in the same R_β → α, CEST_ across the range of T_1β_ and T_2β_ being ±50% of the measured T_1α_ and T_2α_ (Figures S26–S38). These results highlight that the fitting procedure is relatively insensitive to changes in T_1_ and T_2_ for the minor species and demonstrate that the multi‐ and single parameter fitting procedure both provide comparable exchange rates and populations at equilibrium (Figure 5C and E). This represents the first report of equatorial β‐galactosyl triflate population and exchange rates at abundances that fall below the detection limit of 1D ^1^H or ^19^F NMR.

The CEST NMR experiments demonstrated that the C‐4 benzylated (**4**) and C‐4 benzoylated donors (**5** – **6**) all form β‐galactosyl triflates with similar equilibration rates and populations at equilibrium. These intermediates could be responsible for α‐galactoside formation, but the difference in stereoselectivity is not reflected in a difference in galactosyl triflate interconversion or population. In case α‐galactosyl triflate to β‐galactosyl triflate interconversion would be faster than the product forming steps, a Curtin–Hammett scenario would emerge where the difference in product forming rates would determine the overall stereochemical outcome of the reaction. Hence, a higher overall α‐stereoselectivity would be obtained if the C‐4 acyl group would either accelerate the S_N_2‐like displacement of the β‐galactosyl triflate and/or slow down the S_N_2‐like displacement of the α‐galactosyl triflate. Real‐time NMR studies of the glycosylation reaction could aid in understanding the observed reaction selectivity [74, 75]. However, using NMR spectroscopy to study the glycosylation rate is complicated due to the fast nature of the reaction. Therefore, studying the glycosylation rate by NMR could not be achieved because the reaction was complete before recording of the first spectrum was acquired. Instead, we determined the positions of the reaction intermediates and products on the energy landscape using electronic structure calculations. Additionally, the transition states involved in the glycosyl triflate equilibration and the product‐forming steps were calculated to enable the assignment of a Curtin–Hammett scenario [74, 75]. In order to minimize computing costs, the C‐2, C‐3, and C‐6 positions were methylated instead of benzylated. Furthermore, we simplified the system by modeling the reaction with a primary nucleophile using ethanol as a substitute. Calculations were performed using an implicit solvation model of DCM using ORCA6.0.1 [76]. The geometry was optimized on CPCM‐B3LYP‐D3BJ/def2‐TZVP level of theory and the thermal energy was calculated at 213.15 K using the harmonic approximation. The thermal energy was combined with the CPCM‐RI‐MP2/aug‐cc‐pVTZ single point energy to yield the free energy. We assumed that both the galactosyl triflate interconversion and product forming steps are the result of S_N_2‐like substitutions at the anomeric center. We expected the model to describe trends correctly, although not with the same accuracy as the experiments due to the simplification of the reaction intermediate and nucleophile, and solvent effects that are only implicitly accounted for in addition to the error in theory.

**FIGURE 6:**
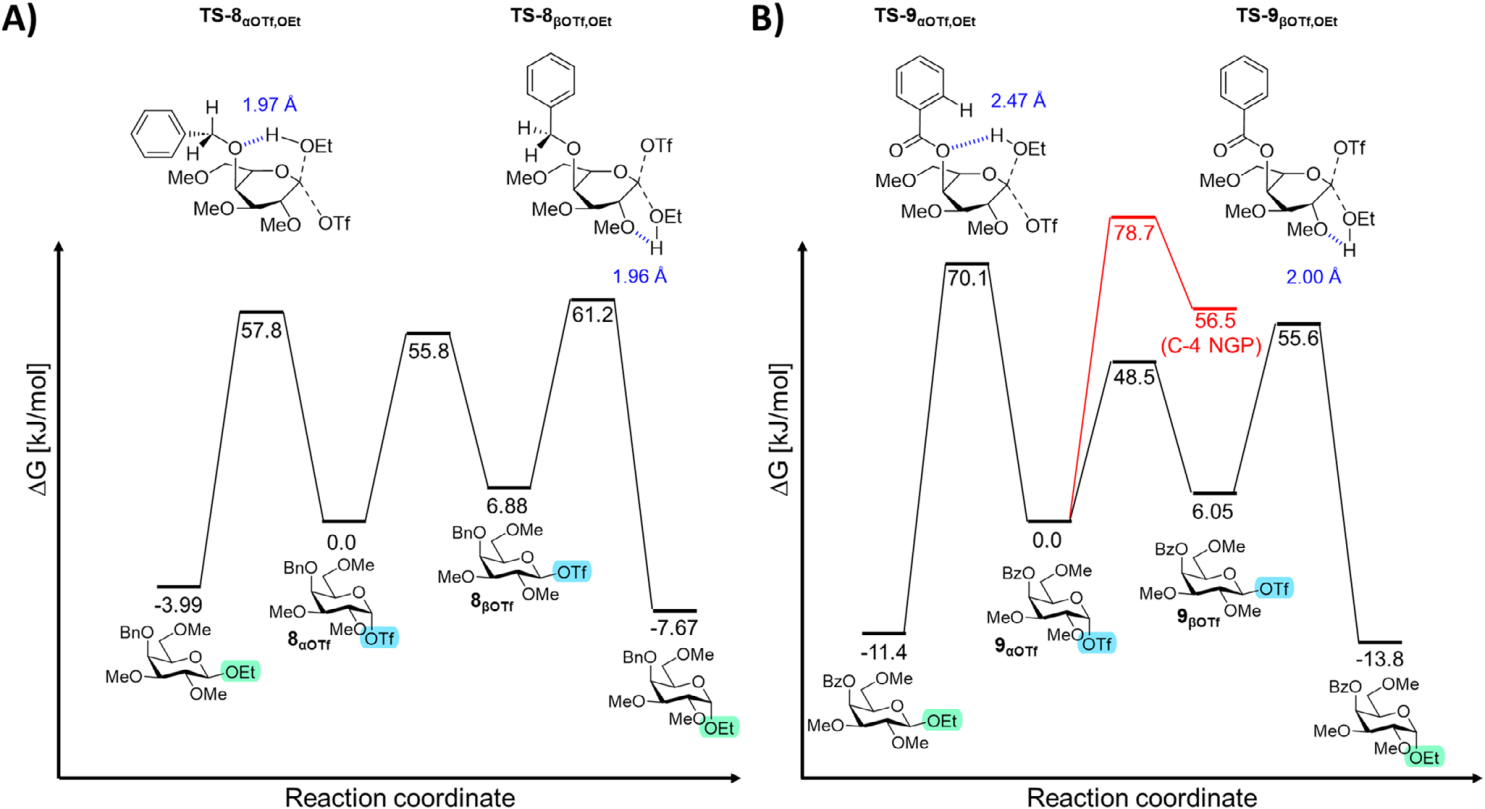
DFT‐computed free energy surface in DCM (ΔG_DCM,_ kJ/mol) for methylated analogues including their interconversion barrier, transition state energies for their reaction with a primary acceptor (ethanol) were also calculated, and the energy of glycosyl α‐triflate was set as the reference point (0 kJ/mol) for the (A) C‐4 benzylated analogues and (B) C‐4 benzoylated analogues including their interconversion barrier and 1,4‐dioxepanium ion formation (B).

Galactosylation with benzylated donor **4** and a primary glycosyl acceptor displayed the poorest reaction selectivity (α:β = 2:1). The calculated ground state energies show that the α‐galactosyl triflate intermediate was more stable than the β‐galactosyl triflate, consistent with its stabilization by the anomeric effect (Figure 6) [77, 78, 79]. With respect to the transition states, interconversion between the triflate intermediates was predicted to proceed with the lowest barrier, which is indicative of a reaction under Curtin‐Hammett control. In this case, the product distribution is not controlled by the ratio of the intermediates, but rather by the relative barriers of the transition states leading to the products. As the transition state energies for α‐ and β‐galactoside formation with ethanol were predicted to be nearly identical, this could explain the poor stereoselectivity observed. Assuming Curtin–Hammet kinetics, we expected a theoretical product distribution of 87/13 favoring the β‐product. We are aware that while ethanol serves as an effective model for simplifying calculations and assessing baseline energetics, it does not fully capture the steric and electronic influences imparted by a carbohydrate acceptor.

Next, we calculated the glycosyl triflate ground‐state energies for benzoylated donor **5** (Figure 6). The ground state energy of the β‐galactosyl triflate was again higher relative to the α‐galactosyl triflate, albeit with a smaller energy difference (Δ*G* = 6.05 kJ/mol). Possibly, the electron‐withdrawing nature of the benzoyl group at C‐4 weakens the anomeric effect of the α‐galactosyl triflate, thereby reducing the relative energy difference between **9_αOTf_** and **9_βOTf_** compared to the benzylated system. The relative interconversion barrier between **9_αOTf_** and **9_βOTf_** was calculated to be lower than product forming steps, hence placing the reaction under Curtin‐Hammett control as the product distribution is governed by the relative energies of the product forming transition states. In sharp contrast to the C‐4 benzyl analogue, the C‐4 benzoyl galactosyl donor did display a significant difference in the relative transition state energies towards product formation. The barrier for forming the β‐galactosyl product (TS‐**9_αOTf_**, OEt) was significantly higher than the barrier of α‐galactosyl product formation (TS‐**9_βOTf_**, OEt), consistent with the observed α‐stereoselectivity. Overall these results indicate that S_N_2‐like substitutions according to a Curtin–Hammett scenario are able to explain the observed selectivity trends. To challenge this assumption, we also investigated two additional mechanistic scenarios that may explain the observed selectivity.

First, we computed the C‐4 acyl NGP pathway and determined both the calculated ground‐state energy of the galactosyl dioxepanium ion and the transition state for its formation (Figure 6B, red line). The calculated barrier for galactosyl dioxepanium ion formation is significantly higher than the activation barriers for both the α‐ and β‐galactosyl triflate interconversion and product‐forming transition states (Figure 6B, red line). This suggests that the galactosyl‐dioxepanium intermediate was unlikely to play a significant role in driving α‐stereoselectivity, consistent with experimental observations. The same calculations were performed for *p*‐anisoylated galactosyl donor **6**. This provided a very similar profile to that of the benzoylated donor **5** (Figure S39), consistent with the experimental observation that the glycosylation stereoselectivity for the *p*‐anisoylated system is similar to that of the benzoylated system. It must be noted that the ground‐state energy of the dioxepanium ion is slightly lower for the *p*‐anisoylated donor, likely due to the added stability from the *p*‐anisoyl group. However, the transition state for the formation of the dioxepanium ion remained similar. These results suggest that the enhanced α‐selectivity is also driven by the transition‐state energy difference towards product formation in this case. The second consideration was to explore the possibility of a front‐side S_N_2 reaction (S_N_2‐f) on the α‐galactosyl triflate [80, 81, 82]. This mechanism was explored by computing the transition state of ethanol reacting with the galactosyl triflates from the C‐4 benzylated and C‐4 benzoylated donors. The transition state energies were found to be 72.8 and 74.2 kJ/mol respectively (Figures S43 and S44). These transitions are 11.6 and 18.6 kJ/mol higher compared to the S_N_2‐like mechanism *via* the β‐galactosyl triflate. Hence, these experiments suggest that the Curtin‐Hammett scenario is most likely responsible for the observed selectivity trends.

In order to understand the emergence of α‐stereoselectivity upon the introduction of a C‐4 acyl group, we examined the differences in transition‐state energies and geometries for product formation more closely (Figure 6). In the case of α‐product formation from the β‐galactosyl triflate, the nucleophile approaches from the α‐face, which is sterically unhindered for all galactosyl donors irrespective of the C‐4 protecting group (**TS‐8_βOTf, OEt_** and **TS‐9_βOTf, OEt_**). A hydrogen bond is observed between the ethanol hydroxyl group (H_ethanol_) and the O‐2 methyl group, possibly assisting α‐galactosyl triflate displacement (Figures S40A, S41A, and S42A). Next, we examined the transition states for product formation *via* the α‐galactosyl triflate (**TS‐8_αOTf, OEt_** and **TS‐9_αOTf, OEt_**). In this case, the nucleophile approaches from the β‐face. In the case of the benzylated donor, we found that the nucleophile forms a hydrogen bond between the ethanol hydroxyl group (*H*_ethanol_) and the *O*‐4 of the benzyl group, possibly stabilizing **TS‐8_αOTf, OEt_** (Figure 40B). The benzyl group is directed away from the pyranose ring system and does not sterically hinder the approach of the nucleophile. In contrast, the C‐4 benzoate group on donor **5** was found to adopt a different conformation in the transition state (**TS‐9_αOTf, OEt_**). The carbonyl oxygen of the secondary ester group is known to adopt a conformation in which it eclipses with the α─C─H bond of the alkyl moiety [26, 83]. This rotamer in combination with the sp^2^ hybridization of the benzylic position restricts the rotation of the acyl substituent placing it on the β‐face. This hinders the approach of the nucleophile from the β‐face, thereby disfavoring β‐glycoside formation and boosting α‐stereoselectivity.

**FIGURE 7:**
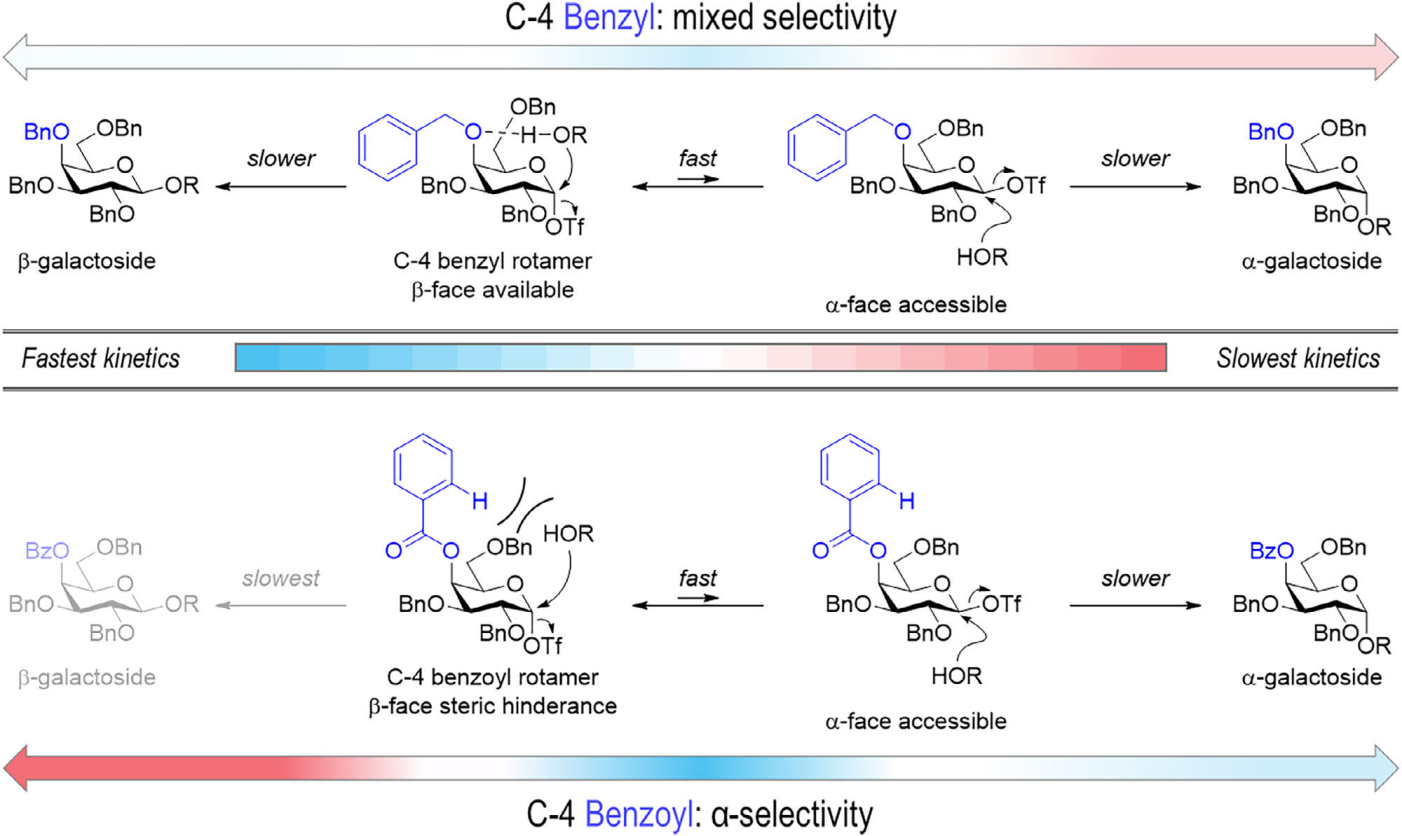
Mechanistic summary.

With this in mind, we subsequently investigated the rotameric mobility of the C‐4 protecting groups and the extent of conformational lock. We calculated the free energy as a function of the dihedral angle between the C_3_‐C_4_‐O_4_‐C_R4_, and a relaxed potential energy scan was performed on DFT level (Figure S45). The dihedral was changed stepwise and fixed while the other internal coordinates were optimized. Similar potential energy surfaces were obtained for the benzoyl and the anisoyl donor. In the lowest energy conformation the C‐4 substituent is pointing away from the carbohydrate ring, while the aromatic ring is positioned perpendicular to the pyranose ring. Two barriers are observed in which the substituent eclipses the C_5_ or the C_3_ substituent. Notably, the C‐4 benzyl donor exhibited lower barriers for the rotation and a lower energy minimum in which the substituent is facing the ring. Moreover, the benzylic carbon is sp^3^ hybridized thereby proving additional rotational freedom and possibilities to position the benzyl substituent away from the anomeric center (Figure S45). Based on these results, we propose that the C‐4 acyl group adopts a conformation in the transition state that places the bulky aromatic phenyl group of the benzoate above the β‐face where it blocks the incoming nucleophile through steric repulsion (Figure 7). Moreover, hydrogen bond formation is prohibited, given that the calculated distances between *H*_ethanol_ and the *O*‐4 were 2.47 Å for the benzoylated system and 2.44 Å for the *p*‐anisoylated system *vs* 1.97 Å in case of the C‐4 benzyl analogue (Figures S41B and S42B). The absence of stabilizing hydrogen bonds, as well as the steric hindrance introduced by the rotamer of the C‐4 benzoate or *p*‐anisoyl group, destabilizes TS‐_αOTf, OEt_. Instead, this favors the α‐selective pathway proceeding *via* the β‐galactosyl triflate under Curtin–Hammett control. In contrast, steric shielding is less pronounced in the C‐4 benzyl system and a hydrogen bond between O‐4 and the acceptor positions the acceptor closer to the electrophilic anomeric site. Hence, these two factors make the barrier towards α‐product or β‐product formation similar, which explains the poor glycosylation selectivity in this donor.

## 3 Conclusion

This study provides a novel, experimentally supported hypothesis for the mechanism of α‐selective galactosylations that utilize C‐4 benzoylated donors. A combination of extensive solution‐phase NMR studies and computational DFT experiments were used to investigate the stereodirecting effect of C‐4 acyl groups during galactosylations in the solution‐phase. We detected β‐galactosyl triflates as key reaction intermediates using exchange NMR and established their exchange kinetics using the Bloch‐McConnell equations. DFT calculations of the galactosyl triflate interconversion and product forming transition states indicate that these reactions are likely under Curtin–Hammett control. A comparative analysis of the transition states reveals that the C‐4 acyl group sterically blocks the β‐face and therefore tips the balance of the Curtin–Hammett scenario towards α‐selectivity by sterically disfavoring nucleophilic attack from the β‐face. These fundamental considerations could aid the development of novel protecting groups or protecting group strategies as well as the design of new stereoselective glycosylation methods in the future.

## Conflicts of Interest

The authors declare no conflicts of interest.

## Supporting Information

The authors have cited additional references within the Supporting Information [1–17].

## Supporting information




**Supporting File 1**: The authors have cited additional references within the Supporting Information [1–17].

## Data Availability

The data that support the findings of this study are available in the supplementary material of this article.
