# Supplementary material for: Elucidating the Stereodirecting Effect of C‐4 Acyl Groups on Galactosyl Donors
Source: Angew Chem Int Ed Engl. 2026 Feb 23;65(14):e21698. doi: 10.1002/anie.202521698 (PMC13023705; doi:10.1002/anie.202521698)
Supplement: Supplementary file 1 — Supporting File 1: The authors have cited additional references within the Supporting Information [1–17]. [file ANIE-65-e21698-s001.pdf]

Supplementary Information

Elucidating the Stereo Directing Effect of C-4 Acyl Groups on Galactosyl Donors

Floor ter Braak,[‡a] Frank F. J. de Kleijne,[‡a] Teun van Wieringen,[‡b] Peter H. Moons,[a] Jonathan Martens,[b] Jos Oomens,[b] Peter A. Korevaar,[*c] Paul B. White,[*a] Thomas J. Boltje[*a]

[a] Synthetic Organic Chemistry, Institute for Molecules and Materials, Radboud University Nijmegen, Heyendaalseweg 135, 6525 AJ, Nijmegen, The Netherlands
[b] FELIX Laboratory, Institute for Molecules and Materials, Radboud University Nijmegen, Toernooiveld 7, 6525 ED, Nijmegen, The Netherlands
[C] Physical Organic Chemistry, Institute for Molecules and Materials, Radboud University Nijmegen, Heyendaalseweg 135, 6525 AJ, Nijmegen, The Netherlands
‡ These authors have contributed equally to this work.
* Corresponding authors: thomas.boltje@ru.nl; paul.white@science.ru.nl; p.korevaar@science.ru.nl.

Table of Contents

Experimental details S2
- Instrumentation S2
- Chemical exchange saturation transfer (CEST) NMR spectroscopy S2
- Selective ^{19}F Exchange Spectroscopy (EXSY) NMR S2
- Relaxation studies S3
- Sample preparation VT NMR S3
- Theoretical background exchange NMR: initial rate approximation S4
- Standard deviations S6
- CEST profile fitting S7
- General synthetic conditions S8
- General procedure 1: Pre-activation conditions for donors **4-6** S8
- General procedure 2: S-oxidation of thioglycosides S9
- Computational protocol S13

Supporting NMR data S15
- Glycosylation results S15
- VT-NMR spectra S18
- Phenyl 4-*O*-(4-methoxybenzoyl-α-^{13}C)-2,3,6-tri-*O*-methyl-1-thio-β-D-galactopyranoside variable temperature NMR experiment S24
- CEST fitting results S27
  - Single data set multiparameter fitting results S27
  - Multiple data set multiparameter fitting results S28
  - Single parameter fitting results S34
  - T_1 and T_2 test for fitting S36

Supporting transition state calculations S43

References S47

Data
- EXSY at different temperatures S49
- EXSY at different concentrations ^{-}OTf S56
- Synthesis spectra S65

Experimental details

Instrumentation: Variable temperature NMR (VT NMR) experiments were conducted on a Bruker 300 MHz Avance III HD nanobay equipped with a BBFO probe and on the JEOL 500 ECZ-R spectrometer equipped with a ROYAL-HFX or ROYAL probe. Low temperature VT operations were achieved with the aid of LN2 evaporator to supply the cold gas, which the probe heated to the desired temperature. The temperature for VT experiments was calibrated against a pure MeOH standard to accurately determine the probe temperature.

Chemical Exchange Saturation Transfer (CEST) NMR spectroscopy: CEST NMR methods were used for ^{1}H, ^{19}F, and ^{13}C NMR spectroscopy. CEST NMR profiles were recorded by incrementing the saturation over a domain of interest. ^{1}H CEST was typically performed in a window between δ_H = 9.0 to 5.0 ppm; ^{19}F CEST was typically performed in a window between δ_F = −72 to −81 ppm; and ^{13}C CEST was performed from δ_C = 210 to 150 ppm. Before each experiment, the 90° pulse was calibrated. A saturation field strength was chosen with regard to experimental duration, resolution, and signal intensity. High resolution (small saturation field strength) leads to weak signals and long experimental times. In contrast, low resolution (large saturation field strength) leads to strong signals and faster experiments. Typically, saturation field strengths were 20 – 40 Hz for ^{1}H CEST, 30 – 60 Hz for ^{19}F CEST, and 80 Hz for ^{13}C CEST. The saturation was achieved either by CW saturation (Bruker) or by pulsed saturation using laminar pulses (JEOL). Finally, typical saturation times were set to 2 – 3 seconds. The relaxation delay was set one second longer then the saturation time (3 – 4 seconds) and the number of scans were typically 2 – 4 per frequency; Two dummy scans were executed.

CEST profiles were made using 1D ^{1}H, ^{19}F, and ^{13}C NMR spectra with saturation at varying positions. A spectrum with no saturation or with off-resonance saturation to both the major and minor observable signal is required as reference named $M_z(0)$. This is typically a 1D spectrum with saturation at δ_H = 9.0 ppm or δ_F = −72 ppm. The peak intensity for the axial triflate (^{1}H CEST), $^{-}$OTf (^{19}F CEST), and acyl carbonyl (^{13}C CEST) resonances, which are the reporter peaks, were determined for all individual spectra after applying phasing and baseline correction. The peak intensity of every spectrum was divided by the peak intensity of the unaltered spectrum ($M_z(0)$) in order to obtain the relative peak intensity of the main observable species as a function of saturation frequency. Plotting the relative intensity of the reporter peak as function of the saturation frequency provides the CEST profile.

^{1}H CEST profiles for CEST fitting were recorded at fixed positions. For example, when recording a CEST profile at 20 Hz saturation strength, then the scanning typically proceeds in increments of 18 Hz. For a CEST profile with a saturation strength of 40 Hz the CEST profile was recorded by incrementing with 35 Hz steps. When recording CEST profiles for fitting, CEST profiles were recorded from 7 ppm to 5 ppm with 20 Hz increments independent of the field strength employed. Saturation was applied for 3 seconds with a 3.2 seconds relaxation delay.

Selective ^{19}F Exchange Spectroscopy (EXSY) NMR

EXSY NMR was utilized in ^{19}F NMR spectroscopy. Before each experiment, the 90° pulse was calibrated and then the selective excitation offset was set to the resonance of interest. The selection pulse typically spanned 0.2 ppm and was on-resonance with the glycosyl triflate. The power levels of the excitation pulse were calculated against the actual 90-degree pulse and the selection window. Relaxation delays were typically

set to 5xT_1 of the triflates, and the number of scans were set to 8 with 2 dummy scans. The longest mix times were determined empirically so that they fit within the initial rate approximation (≈10% conversion). For experiments where the shortest mix time was >80 ms, a pulse sequence that contains a Z-gradient element was used to crush zero-quantum magnetization and clean up artifacts (Bruker: selnogp, JEOL: noesy_1d). When the shortest mix time was <<80 ms, the above experiments were performed without the Z-gradient element.

Relaxation studies: R_1 experiments were performed on a JEOL500 ECZR equipped with a RoyalHFX probe. T_1 values were determined using pseudo 2Ds recorded from several double pulse experiments with varying inversion recovery delays and a 10 seconds relaxation delay. Quick 1D estimates were performed in order to optimize the inversion for the subsequent pseudo-2Ds. The resulting pseudo-2Ds were processed to exponential functions to extract out R_1. T_2 values were determined using a CPMG pulse sequence. The array number was determined after quick 1D estimates were performed in order to optimize the array for the subsequent pseude-2Ds.

Sample preparation VT NMR

Glycosyl sulfoxide donor (1.0 eq, typically 15 mg) and TTBP (2.5 eq) were weighted and dissolved in dried DCM-d_2 (600 μL). Two spherical molecular sieves (4 Å) were added to the NMR tube and the tube was transferred to an analytical scale where internal standard (trimethyl(4-trifluoromethylphenyl)silane) was added. A stock solution of Tf_2O was prepared in DCM-d_2 such that upon addition of stock solution (0.05 mL), the desired amount Tf_2O (1.5 eq) could be added. When the NMR sample and Tf_2O stock solution were ready, the NMR tube was cooled to -80 °C (dry ice/acetone bath) and to the cold tube was added the freshly prepared Tf_2O stock solution (50 μL). The solution generally became (light) yellow upon addition of Tf_2O, was shaken quickly (3x) and was carefully transferred to the NMR. In the probe, the temperature was heated to -60°C for 15-30 minutes. The sample was then cooled to -80°C, at which point a battery of kinetic and characterization experiments were conducted.

Glycosyl thioether (1.0 eq, typically 15 mg), Ph_2SO (1.1 eq), and TTBP (2.5 eq) were weighted and dissolved in dried DCM-d_2 and two spherical molecular sieves (4 Å) were added. A stock solution of Tf_2O was prepared in DCM-d_2, such that the desired amount Tf_2O (1.5 eq) could be added upon addition of stock solution (0.05 mL). When the NMR sample and Tf_2O stock solution were ready, the NMR tube was cooled to -80 °C (dry ice/acetone bath), after which freshly prepared Tf_2O stock solution (50 μL) was added to the cold tube. The solution generally became (light) yellow upon addition of Tf_2O, was shaken quickly (3x) and was carefully transferred to the NMR. In the probe the temperature was heated to -40°C for one hour. The sample was then cooled to -80°, at which point a battery of kinetic and characterization experiments were conducted.

A 1.0 M solution of tetrabutylammonium triflate (TBAT) was prepared in DCM-d_2. Activated molecular sieves (4 Å) were added to the solution and the solution was stored under argon at -80°C. This solution was removed from the -80°C freezer 60 min before the NMR experiment.

NMR experiments at various concentrations triflate anion were executed as described above. After activation at the desired temperature, the probe was set to -60°C. At this

temperature, the sample displayed an exchange ($R_{\alpha \rightarrow OTf, EXSY}$) of about 1 s^{-1} in order to allow sufficient exchange at the lowest concentration and sufficient opportunity to increase as a consequence of the increased triflate concentration before falling out the window of EXSY NMR (see main text). After recording the triflate dissociation under standard conditions, the sample was removed from the probe, quickly stored in a dry ice/acetone bath (-80°C) and the TBAT solution was added (20 μL). The sample was quickly shaken to homogenize the solution (3x) and was carefully transferred to the probe. The sample was locked to DCM-d_2, tuned, and shimmed before performing NMR experiments. After finishing the EXSY and CEST experiments, the cycle was repeated for two more time (by adding 30 μL and 50 μL TBAT solution). In the data workup, the internal standard was used to accurately correct the concentration to volume and TBAT added.

Theoretical background exchange NMR: Initial rate approximation

This section is describes the triflate rate dissociation determination as introduced in previous work.[1] No, or only minor, changes were made to this section since first publication. In order to display the experimental setup and reproduce the experiment without the need to search for the required information in other articles, this section was added to the SI presented here as well.

As outlined in the main text, the *α*-triflate dissociates to form triflate anion in two possible mechanisms (Eq. S1 and S2). The rate laws of the corresponding reactions are described in equation S3 and S4 for the intramolecular glycosyl stabilization and intermolecular glycosyl stabilization respectively. Both processes can proceed simultaneously, hence the overall rate law could be a combination of both (Eq. S5).

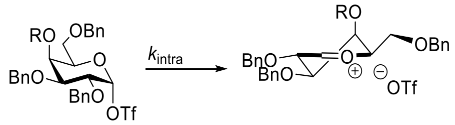

Eq. S1

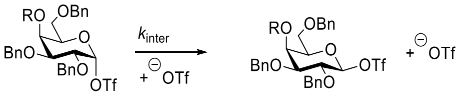

Eq. S2

$$\frac{d[OTf]}{dt} = -\frac{d[\alpha]}{dt} = R_{a\rightarrow OTf} = k_{intra}[\alpha] \quad \text{Eq. S3}$$

$$\frac{d[OTf]}{dt} = -\frac{d[\alpha]}{dt} = R_{a\rightarrow OTf} = k_{inter}[\alpha][^{-}OTf] \quad \text{Eq. S4}$$

$$\frac{d[OTf]}{dt} = -\frac{d[\alpha]}{dt} = R_{a\rightarrow OTf} = k_{intra}[\alpha] + k_{inter}[\alpha][^{-}OTf] \quad \text{Eq. S5}$$

Selective 1D ^{19}F EXSY NMR is a suitable method to study the exchange. By applying a selective excitation pulse on the α-triflate resonance, formation of triflate anion can be measured despite the high population triflate already present in the reaction mixture. This is possible for two main reasons: 1) the resonances of both the *α*-triflate and triflate anion are sufficiently separated to selectively excite the α-triflate in ^{19}F NMR, and, 2) only excited-state nuclei are detected in EXSY NMR. Varying the delay (mix time, τ_m) between the excitation of the *α*-triflate resonance and the spectrum acquisition allows to measure different degrees of conversion for the *α*-triflate into the triflate anion resonance. Eventually, by applying an 'infinitely long' mix time, the equilibrium ratio of *α*-triflate and triflate anion is obtained Figure S5).

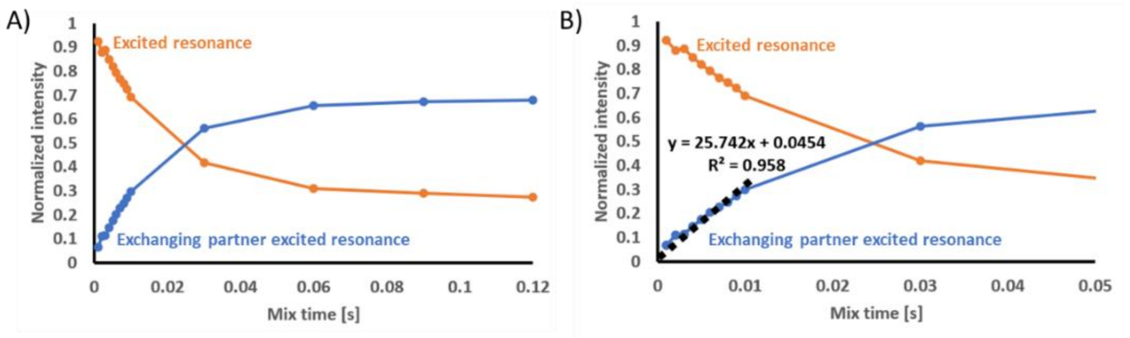

Figure S1: The normalized extent of magnetization transfer from the excited CF3-group of the α-triflate to the unbound triflate is plotted against the mix time. (A) Correlation of EXSY mix time to intensity selected/excited resonance and its exchanging partner that is forming; (B) Displaying the linear initial rate approximation.

The initial triflate formation is linear and kinetics could be described according to the initial rate approximation. Over the initial linear interval, the reaction rate is the α-triflate consumption and triflate anion formation (Eq. S6 and S7). Herein, $[\boldsymbol{\alpha}]_t$ = concentration excited state α-triflate a set mix time after applying the excitation pulse on the α-triflate resonance; $[\boldsymbol{\alpha}]_0$ = concentration excited state α-triflate directly after applying a selective excitation pulse on the α-triflate resonance; $[^{-}\mathbf{OTf}]_t$ = concentration excited state $^{-}$OTf a set mix time after applying the excitation pulse on the α-triflate resonance; $[^{-}\mathbf{OTf}]_0$ = concentration excited state $^{-}$OTf directly after applying a selective excitation pulse on the α-triflate resonance (hence, $[^{-}\mathbf{OTf}]_0 = 0$). Substituting equation S5 into equation S6 gives the concentration excited state α-triflate in terms of concentration and mix time (Eq. S8).

$$\frac{[\boldsymbol{\alpha}]_t - [\boldsymbol{\alpha}]_0}{\tau_m} = -R_{\alpha\rightarrow OTf} \qquad \text{Eq. S6}$$

$$\frac{[^{-}\boldsymbol{OTf}]_t - [^{-}\boldsymbol{OTf}]_0}{\tau_m} = R_{\alpha\rightarrow OTf} \qquad \text{Eq. S7}$$

$$[\boldsymbol{\alpha}]_t = [\boldsymbol{\alpha}]_0 - (k_{intra}[\boldsymbol{\alpha}] + k_{inter}[\boldsymbol{\alpha}][^{-}OTf]) \times \tau_m \qquad \text{Eq. S8}$$

At the very start of the reaction, the concentration *α*-triflate deviates only marginally compared to the starting concentration ($[\boldsymbol{\alpha}]_0$). In accordance to the initial rate approximation, equation S8 becomes equation S9. Additionally, within the initial rate approximation, only an α-triflate conversion of about 5-15% is recorded. Therefore, the concentration excited state triflate ($[^{-}\mathbf{OTf}]$) is sufficiently small (especially compared to the bulk concentration non-excited triflate anion ($[^{-}$OTf$]$)) such that the backwards reaction can be neglected. Subsequently, dividing the equation by $[\boldsymbol{\alpha}]_0$ simplifies the equation to S10.

$$[\boldsymbol{\alpha}]_t = [\boldsymbol{\alpha}]_0 - (k_{intra}[\boldsymbol{\alpha}]_0 + k_{inter}[\boldsymbol{\alpha}]_0[^{-}OTf]) \times \tau_m \qquad \text{Eq. S9}$$

$$\frac{[\boldsymbol{\alpha}]_t}{[\boldsymbol{\alpha}]_0} = 1 - (k_{intra} + k_{inter}[^{-}OTf]) \times \tau_m \qquad \text{Eq. S10}$$

Within NMR spectroscopy, the concentration is proportional (with a constant, c) related to the absolute integral ($\int I_x$) of the observed resonances (Eq. S11 and S12). Substituting equation S10 with S11 and S12 gives the absolute integral of the excited state *α*-triflate resonance as function of mix time (Eq. S13).

$$\int I_{\boldsymbol{\alpha},t} = c \times [\boldsymbol{\alpha}]_t \qquad \text{Eq. S11}$$

$$\int I_{\boldsymbol{\alpha},0} = c \times [\boldsymbol{\alpha}]_0 \qquad \text{Eq. S12}$$

$$\frac{\int I_{\alpha,t}}{\int I_{\alpha,0}} = 1 - (k_{intra} + k_{inter}[^{-}OTf]) \times \tau_m \qquad \text{Eq. S13}$$

One complication is that T_1 relaxation occurs during the mixing time, which will reduce the absolute integral or intensity of the selected and exchanged resonances over time and plotting S13 will result in a multiexponential decay process if $k \leq T_1^{-1}$. This can be easily taken into account if the T_1 is known for each in the absence of exchange. However, if the T_1s for the species are very similar and thus experience similar rates of relaxation, then an approximation can be made where the integral of the α-triflate at t=0 ($I_{\alpha,0}$) is the sum of the integrals of the α-triflate and triflate anion at a given mix time (Eq. S14). This then allows Equation S13 to be rewritten in an internally-consistent manner where the decay is normalized by the measurable peaks for each given mix time. The slope of the plot (Eq. S16) is directly related to the rate constants of both exchange processes (Eq. S1 and S2).

$$\int I_{\alpha,0} = \int I_{\alpha,t} + \int I_{OTf,t} \qquad \text{Eq. S14}$$

$$\frac{\int I_{\alpha,t}}{\int I_{\alpha,t} + \int I_{OTf,t}} = 1 - (k_{intra} + k_{inter}[^{-}OTf]) \times \tau_m \qquad \text{Eq. S15}$$

$$Slope = k_{intra} + k_{inter}[^{-}OTf] \qquad \text{Eq. S16}$$

The rates measured and determined by selective ^{19}F EXSY spectroscopy is, as described above, directly the slope of the normalised absolute integral of the α-triflate ($\int I_{\alpha,t}$) versus mixing time. Hence, equation S16 is in the main text referred to as $R_{\alpha \rightarrow OTf, EXSY}$ (Eq. S17).

$$R_{a \rightarrow OTf,EXSY} = Slope = k_{intra} + k_{inter}[^{-}OTf] \qquad \text{Eq. S17}$$

Standard deviations: The rates determined by EXSY are directly the slope of the normalized α-triflate peak integral against mix time (Eq. S17). The corresponding graph is based on the measured data analysed with the least square regression method. Equations S28 is used to determine the error in the determined EXSY rate based on the measured input data.

$$s_x = \sqrt{\frac{\sum (X_i - \bar{X})^2}{n-1}} \qquad \text{Eq. S28}$$

CEST profile fitting:

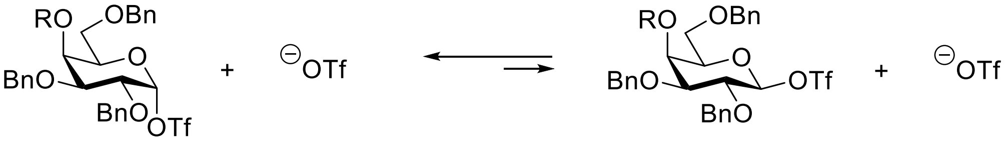

Figure S2: Two-sided exchange model for CEST fitting.

For CEST fitting the exchange model in Figure S2 is considered. The simulations are based on work reported by the Gschwind lab.[2] The Bloch-McConnell equations describing the two-sides exchange system are described in equations S29-34.

$$\frac{dM_x^\alpha}{dt} = -\Omega_\alpha M_y^\alpha - R_2^\alpha M_x^\alpha - R_{\alpha\to\beta,\ CEST} M_x^\alpha + R_{\beta\to\alpha,\ CEST} M_x^\beta \qquad \text{Eq. S29}$$

$$\frac{dM_x^\beta}{dt} = -\Omega_\beta M_y^\beta - R_2^\beta M_x^\beta - R_{\beta\to\alpha,\ CEST} M_x^\beta + R_{\alpha\to\beta,\ CEST} M_x^\alpha \qquad \text{Eq. S30}$$

$$\frac{dM_y^\alpha}{dt} = -\Omega_\alpha M_x^\alpha - R_2^\alpha M_y^\alpha - R_{\alpha\to\beta,\ CEST} M_y^\alpha + R_{\beta\to\alpha,\ CEST} M_y^\beta - \varpi M_z^\alpha \qquad \text{Eq. S31}$$

$$\frac{dM_y^\beta}{dt} = -\Omega_\beta M_x^\beta - R_2^\beta M_y^\beta - R_{\beta\to\alpha,\ CEST} M_y^\beta + R_{\alpha\to\beta,\ CEST} M_y^\alpha - \varpi M_z^\beta \qquad \text{Eq. S32}$$

$$\frac{dM_z^\alpha}{dt} = -R_1^\alpha\,(M_z^\alpha - M_0^\alpha)\ - R_{\alpha\to\beta,\ CEST} M_z^\alpha + R_{\beta\to\alpha,\ CEST} M_z^\beta + \varpi M_y^\alpha \qquad \text{Eq. S33}$$

$$\frac{dM_z^\beta}{dt} = -R_1^\beta\left(M_z^\beta - M_0^\beta\right) - R_{\beta\to\alpha,\ CEST} M_z^\beta + R_{\alpha\to\beta,\ CEST} M_z^\alpha + \varpi M_y^\beta \qquad \text{Eq. S34}$$

Herin,

- $M_{x,y,z}^\alpha$ and $M_{x,y,z}^\beta$ are magnetization of the α-triflate and β-triflate in either the x, y, or, z planes.
- $R_{\alpha\to\beta,\ CEST}$ and $R_{\beta\to\alpha,\ CEST}$ are normalized rates.

  Normalized rates are defined: $R_{\alpha\to\beta,\ CEST} = \frac{\mathrm{R}_{\alpha\to\beta}}{[\alpha]}$ and $R_{\beta\to\alpha,\ CEST} = \frac{\mathrm{R}_{\beta\to\alpha}}{[\beta]}$.
  Where $R_{\alpha\to\beta} = k_{\alpha\to\beta}[\alpha][^{-}OTf]$ and $R_{\beta\to\alpha} = k_{\beta\to\alpha}[\beta][^{-}OTf]$
- Ω_α and Ω_β are offset frequencies of α-triflate and β-triflate anomeric protons respectively.
- R_2^α and R_2^β are spin-spin relaxation times for the anomeric proton for the α-triflate and β-triflate respectively.
- R_1^α and R_1^β are spin-lattice relaxation rates for the anomeric proton for the α-triflate and β-triflate respectively.

  Relaxation is defined $R_1^\alpha = \frac{1}{T_{1,\alpha}}$, $R_2^\alpha = \frac{1}{T_{2,\alpha}}$, $R_1^\beta = \frac{1}{T_{1,\beta}}$, and $R_2^\beta = \frac{1}{T_{2,\beta}}$.
- ϖ is strength of RF saturation.

CEST fitting was performed using the simulation method reported by Gschwind and co-workers[2] which was based on obtaining numerical solution for the Bloch-McConnell equations as described by the labs of Van Zijl[3-4] and Sherry.[5] As we noted that the numerical solutions for the Bloch-McConnel equations allowed reasonable fits for multiple combinations of $R_{\beta\to\alpha,\ CEST}$ and the β-triflate population (P_β), the CEST fitting procedure was adjusted to include CEST profiles recorded at four different RF

saturation strengths (20, 30, 40, and 60 Hz). This approach was based on reports by Zaiss and co-workers.[6]

For the fitting input values are: M_0^{α}, which is the initial magnetization of the α-triflate its anomeric proton resonance, and was set to 1. ϖ was set at 20, 30, 40, and 60 Hz for the corresponding datasets. $T_{1,\alpha}$, and $T_{1,\beta}$, which were both fixed to the value recorded for the α-triflate its anomeric resonance using a double pulse inversion recovery experiment. $T_{2,\alpha}$, and $T_{2,\beta}$, which were both fixed to the value recorded for the α-triflate its anomeric resonance using a CPMG experiment. Fitted were M_0^{β} and $R_{\beta\rightarrow\alpha,\ CEST}$, herein: M_0^{β}, is the initial magnetization of the β-triflate its anomeric proton resonance, and $R_{\beta\rightarrow\alpha,\ CEST}$ is the normalized rate of β- to α-triflate interconversion.

General synthetic conditions: Synthetic product characterisations were recorded with a Bruker 500 MHz AVANCE III spectrometer or JEOL 500 ECZ-R spectrometer. The Bruker 500 MHz Avance III spectrometer is equipped with a Prodigy BB cryoprobe. The JEOL 500 ECZ-R spectrometers were equipped with either a SuperCOOL broadband probe, ROYAL broadband probe, or ROYAL HFX broadband probe. Chemical shifts are reported in parts per million (ppm) with tetramethylsilane (TMS) as the internal standard or solvent residual signals (SRP) if stated otherwise. ^{1}H NMR spectroscopic data is presented as follows: chemical shift, multiplicity (s = singlet, d = doublet, t = triplet, dd = doublet of doublets, dt = doublet of triplets, m = multiplet and/or multiple resonances), coupling constant (J) in hertz (Hz), integration and assignments. All NMR signals were assigned based on ^{1}H NMR, ^{13}C NMR, COSY, HSQC, HMBC, TOCSY, NOESY and ROESY experiments. Mass spectra were recorded with a JEOL JMST100CS AccuTOF mass spectrometer. Automatic silica-flash column chromatography was done with a Biotage Isolera Spektra One, using pre-packed cartridges ultrapure irregular silica gel (Screening Devices, 40-63 μm, 60 Å). TLC analysis was conducted on Silica gel F254 (Merck KGaA) with detection by UV absorption (254 nm) where applicable and by dipping in a stain followed by heating. Stains used for TLC analysis were either 10% sulphuric acid in MeOH, cerium molybdate stain (0.03 M $(NH_4)6Mo_7O_{24}\cdot 4H_2O$; 6 mM $Ce(NH_4)_4(SO_4)_4\cdot 2H_2O$; 1 M H_2SO_4 in H_2O), potassium permanganate (0.06 M $KMnO_4$; 0.5 M, K_2CO_3; 0.02 M NaOH in H_2O). Reactions that used anhydrous solvents were performed under Schlenk conditions and were conducted under an argon atmosphere. Molecular Sieves (0.4 nm) were activated overnight by heating *in vacuo* at 150°C.

General procedure 1: Pre-activation conditions for donors 4-6
A solution of the oxidized thioglycoside donor (1.0 equiv, 50 mg) in dry DCM (0.05 M) was prepared and stirred over activated 3 Å molecular sieves under a nitrogen atmosphere for 30 minutes. TTBP (2.5 equiv) and Ph_2SO (1.3 equiv) were added, and the reaction mixture was cooled to −80 °C. Tf_2O (1.3 equiv) then warmed to −60 °C and stirred for 30 minutes to activate the donor. After activation, the reaction was cooled back to −80 °C, and the acceptor (2.0 equiv) in dry DCM (0.5 M) was added. The reaction was allowed to warm slowly to room temperature over 5 hours. Upon completion, the reaction was quenched with saturated aqueous $NaHCO_3$ at −60 °C and diluted with DCM. The organic layer was washed with water and brine, dried over

$MgSO_4$, filtered, and concentrated *in vacuo* The crude product was analyzed by quantitative HSQC to determine selectivity, and coupled HSQC was performed to assess the stereochemistry at C1-H.

General procedure 2: S-oxidation of thioglycosides

To a solution of the thioglycoside (1.0 equiv.) in DCM (0.10 M) at -78 °C was added *m*-CPBA (1.1 equiv., 70% Wt) in DCM dropwise. The reaction mixture was stirred for 3h after which it was then quenched by the addition of sat. aqueous $Na_2S_2O_3$ and diluted using DCM. The organic layer was washed with $NaHCO_3$ (sat. aq.; 2x) and brine, dried over $MgSO_4$ and concentrated *in vacuo*. The residue was purified by silica gel flash column chromatography (0→25% EtOAc/n-heptane, *v*/*v*).

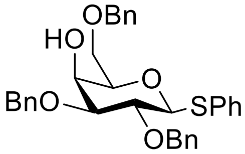

Phenyl 2,3,6-tri-*O*-benzyl-1-thio-β-D-galactopyranoside

To a solution of phenyl 2,3-di-*O*-benzyl-4,6-*O*-benzylidene-1-thio-β-D-galactopyranoside[7] (3.40 g, 6.30 mmol, 1 equiv.) in DCM (0.1 M, 63 mL) were added triethylsilane (6.0 mL, 38 mmol, 6 equiv.) and trifluoroacetic anhydride (0.89 mL, 6.30 mmol, 1 equiv.). The resulting solution was cooled to 0 °C where after trifluoroacetic acid (2.90 mL, 38 mmol, 6 equiv.) was added dropwise. The mixture was allowed to warm up to room temperature and was stirred for 3h. The solution was diluted with DCM and quenched with $NaHCO_3$ (sat. aq., 100 mL). The aqueous phase was extracted using DCM (3x 100 mL) and the combined organic phases were washed with water (50 mL), dried over Na_2SO_4, filtered and concentrated *in vacuo*. Phenyl 2,3,6-tri-*O*-benzyl-1-thio-β-D-galactopyranoside (2.85 g, 84%) was obtained as a white solid after recrystallization from hot ethanol.

TLC: R_f 0.44 (EtOAc/n-heptane, 1:1, *v*/*v*); **^{1}H NMR** (500 MHz, CHLOROFORM-*D*) δ 7.69 – 7.50 (m, 2H), 7.49 – 7.16 (m, 18H), 4.83 (d, *J* = 10.2 Hz, 1H), 4.77 – 4.66 (m, 3H), 4.64 (d, *J* = 9.8 Hz, 1H, C1-H), 4.57 (s, 2H), 4.16 – 4.06 (m, 1H, C4-H), 3.83 – 3.75 (m, 2H, C2-H; C6-H), 3.59 (ddd, *J* = 12.1, 7.8, 4.3 Hz, 2H, C3-H; C5-H), 2.51 (dd, *J* = 2.5, 1.0 Hz, 1H, C4-OH). **^{13}C NMR** (126 MHz, CHLOROFORM-*D*) δ 138.2, 138.0, 137.7, 133.9 (all quaternary), 131.8, 128.9, 128.6, 128.5, 128.4, 128.3, 128.0, 127.9, 127.8, 127.8, 127.8, 127.4 (all aromatic), 87.78 (C-1), 82.6 (C-3), 77.1 (C-2, C-5), 75.8, 73.7, 72.2, 69.5 (C-6), 66.9 (C-4). **HRMS:** $[M + Na]^+$ calcd. for $C_{33}H_{34}O_5S$ 543.2200, found 543.2208.

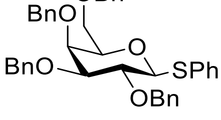

Phenyl 2,3,4,6-tetra-*O*-benzyl-1-thio-β-D-galactopyranoside

To a solution of phenyl 2,3,6-tri-*O*-benzyl-1-thio-β-D-galactopyranoside (400 mg, 737 µmol, 1 equiv.) in DMF (3.69 mL, 0.2 M) was added NaH (35.4 mg, 60% Wt, 1.47 mmol, 2 Equiv.) at 0 °C. After 15 minutes benzyl bromide (131 µL, 1.1 mmol, 1.5 equiv.) was added and the reaction mixture was stirred for 12h at rt. The reaction mixture was quenched with the addition of MeOH (5 mL) and H_2O (10 mL) and thereafter diluted with EtOAc (50 mL). Subsequently, the water layer was extracted with EtOAc (3 x 25 mL). The combined organic layers were washed with $NaHCO_3$ (sat. aq., 10 mL), brine (10 mL), dried over $MgSO_4$ and concentrated *in vacuo*. The crude oil was then purified using silica gel flash

column chromatography (0→20% EtOAc/n-heptane, *v*/*v*) to obtain phenyl 2,3,4,6-tetra-*O*-benzyl-1-thio-β-D-galactopyranoside as a waxy solid (429 mg, 92%)

TLC: R_f 0.78 (EtOAc/n-heptane, 1:1, *v*/*v*); **^{1}H NMR** (500 MHz, CHLOROFORM-*D*) δ 7.56 – 7.47 (m, 2H), 7.36 – 7.17 (m, 20H), 7.13 (dd, *J* = 5.1, 1.9 Hz, 3H), 4.91 (d, J = 11.5 Hz, 1H), 4.73 (d, J = 10.2 Hz, 1H), 4.68 – 4.66 (m, 3H), 4.55 (d, J = 11.5 Hz, 1H), 4.42 (d, J = 11.7 Hz, 1H), 4.37 (d, J = 11.7 Hz, 1H), 3.93 (dd, *J* = 2.7, 0.9 Hz, 1H, C4-H), 3.88 (t, *J* = 9.4 Hz, 1H, C2-H), 3.60 (dd, *J* = 6.3, 1.6 Hz, 2H, C6-H), 3.57 – 3.52 (m, 2H, C3-H; C5-H). **^{13}C NMR** (126 MHz, CHLOROFORM-*D*) δ 138.9, 138.5, 138.4, 138.0, 134.3 (all quaternary), 131.6, 128.9, 128.6, 128.5, 128.3, 128.1, 128.0, 127.9, 127.9, 127.8, 127.7, 127.6, 127.2 (all aromatic), 87.9 (C-1), 84.3 (C-3), 77.6 (C-2; C-5), 75.8, 74.6, 73.7 (C-4), 73.7, 72.9, 68.9 (C-6). **HRMS:** [M + Na]$^+$ calcd. for $C_{40}H_{40}O_5S$ 655.2489, found 655.2490.

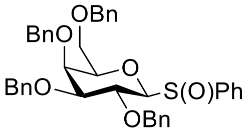

Phenyl 2,3,4,6-tetra-*O*-benzyl-1-thiosulfinyl-β-D-galactopyranoside (4)

Via general S-oxidation procedure starting with phenyl 2,3,4,6-tetra-*O*-benzyl-1-thio-β-D-galactopyranoside (400 mg, 632 µmol, 1 equiv.) to afford phenyl 4-*O*-(*p*-methoxybenzoyl)-2,3,6-tri-*O*-benzyl-1-thiosulfinyl-β-D-galactopyranoside as a mixture of diastereomers (364 mg, 89 %)

Diastereomer 1

Clear oil, 121 mg. **TLC**: R_f 0.52 (EtOAc/n-heptane, 1:1, *v*/*v*); **^{1}H NMR** (500 MHz, CHLOROFORM-*D*) δ 7.70 – 7.62 (m, 2H), 7.49 – 7.18 (m, 21H), 7.14 – 7.05 (m, 2H), 5.02 (d, J = 10.2 Hz, 1H), 4.98 (d, J = 10.2 Hz, 1H), 4.92 (d, J = 11.8 Hz, 1H), 4.75 (d, J = 11.8 Hz, 1H), 4.72 (d, J = 11.8 Hz, 1H), 4.62 (d, J = 11.8 Hz, 1H), 4.48 (t, J = 9.6 Hz, 1H, C2-H), 4.16 (s, 2H), 3.94 (d, J = 9.6 Hz, 1H, C1-H), 3.89 (d, J = 2.7 Hz, 1H, C4-H), 3.68 (dd, J = 9.5, 2.8 Hz, 1H, C3-H), 3.54 – 3.46 (m, 1H, C6-H), 3.45 – 3.36 (m, 2H, C5-H; C6-H).**^{13}C NMR** (126 MHz, CHLOROFORM-D) δ 140.2, 138.5, 138.1, 138.0, 138.0 (all quaternary), 130.9, 128.9, 128.7, 128.6, 128.6, 128.5, 128.4, 128.4, 128.1, 128.0, 127.9, 127.8, 127.8, 127.7, 125.5 (all aromatic), 94.2 (C-1), 84.4 (C-3), 79.2 (C-5), 76.1, 74.5, 74.0 (C-2), 73.5, 73.3 (C-4), 72.8, 69.1 (C-6). **HRMS:** [M + Na]$^+$ calcd. for $C_{40}H_{40}O_6S$ 671.2438, found 671.2434.

Diastereomer 2

clear oil, 243 mg. **TLC**: R_f 0.44 (EtOAc/n-heptane, 1:1, *v*/*v*); **^{1}H NMR** (500 MHz, CHLOROFORM-*D*) δ 7.61 – 7.46 (m, 2H), 7.43 – 7.14 (m, 21H), 7.06 (m, 2H), 4.86 – 4.73 (m, 3H), 4.66 (d, *J* = 11.6 Hz, 1H), 4.59 (d, *J* = 11.6 Hz, 1H), 4.49 – 4.42 (m, 2H, C1-H), 4.39 (d, *J* = 11.7 Hz, 1H), 4.35 (d, *J* = 11.8 Hz, 1H), 4.00 (t, *J* = 9.0 Hz, 1H, C2-H), 3.90 (dd, *J* = 2.7, 1.3 Hz, 1H, C4-H), 3.65 (ddd, *J* = 7.0, 5.7, 1.9 Hz, 2H, C3-H; C5-H), 3.60 (dd, *J* = 9.2, 5.5 Hz, 1H, C6-H), 3.48 (dd, *J* = 9.2, 7.3 Hz, 1H, C6-H). **^{13}C NMR** (126 MHz, CHLOROFORM-*D*) δ 140.3, 138.7, 138.1, 137.9, 137.9 (all quaternary), 131.2, 128.7, 128.6, 128.6, 128.4, 128.3, 128.1, 128.0, 128.0, 127.8, 127.8, 127.4, 127.3, 126.2 (all aromatic), 95.5 (C-1), 84.0 (C-3), 77.6 (C-5), 74.6, 74.2, 73.8 (C-2), 73.7, 72.8 (C-4), 72.5, 68.2 (C-6). **HRMS:** [M + Na]$^+$ calcd. For $C_{40}H_{40}O_6S$ 671.2438, found 671.2434.

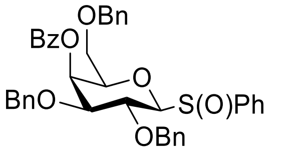

Phenyl 4-*O*-benzoyl-2,3,6-tri-*O*-benzyl-1-thiosulfinyl-β-D-galactopyranoside (5)

To a solution of phenyl 2,3,6-tri-*O*-benzyl-1-thio-β-D-galactopyranoside (425 mg, 783 µmol, 1 equiv.) in pyridine (0.1 M, 7.83 mL) were added Benzoyl chloride (364 µl, 3.13 mmol, 4 equiv.) and DMAP (10 mg, µmol, 0.1 equiv.) at 0°C. The resulting mixture was then allowed to warm up to room temperature and stirred for 12h. Subsequently, the solvent was removed *in vacuo* after which the residue was redissolved in DCM (100 mL) and washed with $NaHCO_3$ (sat. aq.; 25 mL) and brine (25 mL) The organic layer was dried over Na_2SO_4 and concentrated to get the benzoyl derivative as an oil, which was purified using silica gel flash column chromatography (0→25% EtOAc/n-heptane, *v*/*v*) to obtain phenyl 4-*O*-benzoyl-2,3,6-tri-*O*-benzyl-β-D-thiogalactopyranoside (440 mg, 87 %) as a clear oil. **TLC**: R_f 0.74 (EtOAc/n-heptane, 1:1, *v*/*v*; ^{1}H NMR data is in accordance with values reported in literature.[8] The isolated compound was then S-oxidized *via* general S-oxidation procedure starting with phenyl 4-*O*-benzoyl-2,3,6-tri-*O*-benzyl-β-D-thiogalactopyranoside (200 mg, 309 µmol, 1 equiv.) to afford phenyl 4-*O*-benzoyl-2,3,6-tri-*O*-benzyl-1-thiosulfinyl-β-D-galactopyranoside as a mixture of diastereomers (176 mg, 86%).

Diastereomer 1

Clear oil, 90 mg. **TLC**: R_f 0.53 (EtOAc/n-heptane, 1:1, *v*/*v*); **^{1}H NMR** (500 MHz, CHLOROFORM-*D*) δ 8.21 – 8.09 (m, 2H), 7.75 – 7.09 (m, 23H), 5.85 (dd, *J* = 3.2, 1.1 Hz, 1H, C4-H), 4.97 (d, *J* = 10.3 Hz, 1H), 4.91 (d, *J* = 10.3 Hz, 1H), 4.88 (d, *J* = 11.5 Hz, 1H), 4.57 (d, *J* = 11.5 Hz, 1H), 4.38 (t, *J* = 9.5 Hz, 1H, C2-H), 4.24 (d, *J* = 11.7 Hz, 1H), 4.21 (d, *J* = 11.7 Hz, 1H), 4.04 (d, *J* = 9.8 Hz, 1H, C1-H), 3.84 (dd, *J* = 9.3, 3.2 Hz, 1H, C3-H), 3.71 (td, *J* = 6.1, 1.2 Hz, 1H, C5-H), 3.54 (dd, *J* = 10.1, 6.1 Hz, 1H, C6-H), 3.42 (dd, *J* = 10.1, 6.1 Hz, 1H, C6-H).**^{13}C NMR** (126 MHz, CHLOROFORM-*D*) δ 165.8 (C=O), 140.0, 137.8, 137.5, 137.4 (all quaternary), 133.6, 133.3, 131.0, 130.2, 129.8, 129.5 (quaternary), 128.8, 128.6, 128.5, 128.4, 128.4, 128.3, 128.2, 128.2, 128.0, 127.8, 127.8, 127.7, 125.4 (all aromatic), 93.5 (C-1), 81.3 (C-3), 77.6 (C-5), 76.1, 73.6, 73.3 (C-2), 71.6, 68.3 (C-6), 67.1 (C-4). **HRMS:** $[M + Na]^+$ calcd. for $C_{40}H_{38}O_7S$ 685.2230, found 685.2214.

Diastereomer 2

Milky oil, 86 mg. **TLC**: R_f 0.33 (EtOAc/n-heptane, 1:1, *v*/*v*); **^{1}H NMR** (500 MHz, CHLOROFORM-*D*) δ 7.70 – 7.63 (m, 2H), 7.61 – 7.49 (m, 4H), 7.47 – 7.37 (m, 4H), 7.33 – 7.14 (m, 15H), 5.86 (dd, *J* = 3.0, 1.0 Hz, 1H, H-4), 4.86 (d, *J* = 11.0 Hz, 1H), 4.85 (d, *J* = 11.0 Hz, 1H), 4.70 (d, *J* = 11.0 Hz, 1H), 4.66 (d, *J* = 9.7 Hz, 1H), 4.49 – 4.38 (m, 3H), 3.93 (ddd, *J* = 7.5, 5.5, 1.1 Hz, 1H, H-5), 3.85 (dd, *J* = 9.0, 3.0 Hz, 1H, H-3), 3.72 (dd, *J* = 9.8, 8.9 Hz, 1H, H-2), 3.67 (dd, *J* = 9.5, 5.5 Hz, 1H, H-6), 3.43 (dd, *J* = 9.5, 7.5 Hz, 1H, H-6).**^{13}C NMR** (126 MHz, CHLOROFORM-*D*) δ 165.6 (C=O), 139.6, 138.1, 137.8 (all quaternary), 137.6, 133.6, 131.6, 130.3, 129.8 (quaternary), 129.0, 128.8, 128.8, 128.7, 128.3, 128.2, 128.2, 127.0 (all aromatic), 94.9 (C-1), 82.1 (C-3), 76.9 (C-5), 74.8, 74.2, 73.1 (C-2), 72.0, 67.9 (C-6), 66.7 (C-5). **HRMS:** $[M + Na]^+$ calcd. for $C_{40}H_{38}O_7S$ 685.2230, found 685.2214.

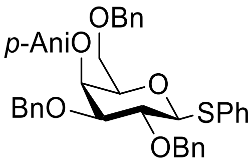

phenyl 4-*O*-(*p*-methoxybenzoyl)-2,3,6-tri-*O*-benzyl-1-thio-β-D-galactopyranoside

To phenyl 2,3,6-tri-*O*-benzyl-1-thio-β-D-galactopyranoside (400 mg, 737 µmol, 1 equiv.) dissolved in DCM (7.37 mL, 0.1 M) was added 4-methoxybenzoic acid (123 mg, 811 µmol, 1.1 equiv.). The mixture was activated by the addition of DIC (173 µL, 1.11 mmol, 1.5 equiv.) and DMAP (9 mg, 73.7 µmol, 0.1 equiv.) where after it was stirred for 12h at rt. The reaction mixture was then filtered and concentrated *in vacuo*. The crude material was purified using column purified using silica gel flash column chromatography (0→15% EtOAc/Toluene, *v*/*v*) to afford phenyl 4-*O*-(*p*-methoxybenzoyl)-2,3,6-tri-*O*-benzyl-1-thio-β-D-galactopyranoside (278 mg, 56%) as a clear oil.

TLC: R_f 0.48 (EtOAc/n-heptane, 1:1, *v*/*v*); **^{1}H NMR** (500 MHz, CHLOROFORM-*D*) δ 7.98 – 7.88 (m, 2H), 7.42 – 7.11 (m, 20H), 6.90 (d, *J* = 8.9 Hz, 2H), 5.83 (dd, *J* = 3.1, 1.0 Hz, 1H, H-4), 4.82 (d, *J* = 11.2 Hz, 1H), 4.71 (s, 2H), 4.67 (d, *J* = 9.3 Hz, 1H, H-1), 4.49 (d, J = 11.2 Hz, 1H), 4.48 (d, *J* = 11.6 Hz, 1H), 4.43 (d, *J* = 11.8 Hz, 1H), 3.87 (s, 4H, H-5; CH_3), 3.72 (dd, *J* = 9.1, 3.1 Hz, 1H, H-3), 3.70 – 3.67 (m, 1H, H-2), 3.67 – 3.64 (m, 1H, H-6), 3.56 (dd, *J* = 9.7, 6.6 Hz, 1H, H-6).**^{13}C NMR** (126 MHz, CHLOROFORM-*D*) δ 165.5 (C=O), 163.7, 138.4, 137.8, 133.2 (all quaternary), 133.0, 132.2, 129.0, 128.5, 128.5, 128.5, 128.4, 128.3, 128.0, 127.9, 127.84, 127.8, 122.3 (quaternary), 113.8 (all aromatic), 87.3 (C-1), 81.6 (C-2), 76.8 (C-3), 76.5 (C-5), 75.8, 73.9, 71.9, 68.7 (C-6), 67.2 (C-4), 55.6. **HRMS:** $[M + Na]^+$ calcd. for $C_{41}H_{40}O_7S$ 699.2387, found 699.2373.

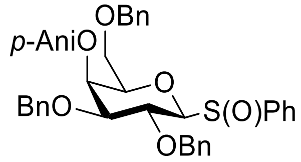

Phenyl 4-*O*-(*p*-methoxybenzoyl)-2,3,6-tri-*O*-benzyl-1-thiosulfinyl-β-D-galactopyranoside (6)

Via general S-oxidation procedure starting with phenyl 4-*O*-(*p*-methoxybenzoyl)-2,3,6-tri-*O*-benzyl-1-thio-β-D-galactopyranoside (170 mg, 251 µmol, 1 equiv.) to afford phenyl 4-*O*-(*p*-methoxybenzoyl)-2,3,6-tri-*O*-benzyl-1-thiosulfinyl-β-D-galactopyranoside as a mixture of diastereomers (113 mg, 65%).

Diastereomer 1

Clear oil, 53 mg **TLC**: R_f 0.52 (EtOAc/n-heptane, 1:1, *v*/*v*); **^{1}H NMR** (500 MHz, CHLOROFORM-*D*) δ 8.13 – 8.07 (m, 2H), 7.76 – 7.09 (m, 20H), 7.00 – 6.92 (m, 2H), 5.82 (dd, *J* = 3.2, 1.1 Hz, 1H, C4-H), 4.98 (d, *J* = 10.3 Hz, 1H), 4.91 (d, *J* = 10.3 Hz, 1H), 4.87 (d, *J* = 11.5 Hz, 1H), 4.56 (d, *J* = 11.5 Hz, 1H), 4.38 (t, *J* = 9.6 Hz, 1H, C2-H), 4.22 (d, *J* = 2.2 Hz, 2H), 4.04 (d, *J* = 9.8 Hz, 1H, C1-H), 3.88 (s, 3H), 3.83 (dd, *J* = 9.3, 3.2 Hz, 1H. C3-H), 3.70 (td, *J* = 6.0, 1.1 Hz, 1H, C5-H), 3.54 (dd, *J* = 10.1, 5.9 Hz, 1H, C6-H), 3.42 (dd, *J* = 10.1, 6.2 Hz, 1H, C6-H). **^{13}C NMR** (126 MHz, CHLOROFORM-*D*) δ 165.5 (C=O), 163.7, 139.9, 137.8, 137.6, 137.5 (all quaternary), 133.6, 132.3, 131.0, 130.2, 129.8, 128.8, 128.4, 128.4, 128.3, 128.3, 128.2, 128.0, 127.8, 127.8, 127.7, 125.4, 121.9 (quaternary), 113.9 (all aromatic), 93.5 (C-1), 81.3 (C-3), 77.8 (C-5), 76.1, 73.6, 73.3 (C-2), 71.5, 68.5 (C-6), 66.7 (C-4), 55.5. **HRMS:** $[M + Na]^+$ calcd. for $C_{41}H_{40}O_8S$ 715.2336, found 715.2319.

Diastereomer 2

Clear oil, 60 mg. **TLC**: R_f 0.36 (EtOAc/n-heptane, 1:1, *v/v*); **^{1}H NMR** (500 MHz, CHLOROFORM-*D*) δ 7.66 – 7.11 (m, 20H), 6.94 – 6.65 (m, 2H), 5.82 (dd, *J* = 3.0, 1.1 Hz, 1H, C4-H), 4.86 (d, *J* = 11.0 Hz, 1H), 4.84 (d, *J* = 11.0 Hz, 1H), 4.69 (d, *J* = 11.0 Hz, 1H), 4.64 (d, *J* = 9.7 Hz, 1H, C1-H), 4.49 – 4.38 (m, 3H), 3.95 – 3.87 (m, 4H, C5-H), 3.83 (dd, *J* = 8.9, 3.0 Hz, 1H, C3-H), 3.74 (t, *J* = 9.3 Hz, 1H, C2-H), 3.66 (dd, *J* = 9.6, 5.6 Hz, 1H, C6-H), 3.43 (dd, *J* = 9.6, 7.2 Hz, 1H, C6-H).**^{13}C NMR** (126 MHz, CHLOROFORM-*D*) δ 164.1 (C=O), 162.7, 138.5, 136.9, 136.7, 136.4 (all quaternary), 131.1, 130.3, 127.8, 127.6, 127.5, 127.4, 127.0, 127.0, 127.0, 126.9, 125.6, 121.0 (quaternary), 112.8 (all aromatic), 93.8 (C-1), 80.9 (C-3), 75.8 (C-5), 73.5, 72.9, 71.8 (C-2), 70.6, 66.8 (C-6), 65.1, 54.6. **HRMS:** $[M + Na]^+$ calcd. for $C_{41}H_{40}O_8S$ 715.2336, found 715.2319.

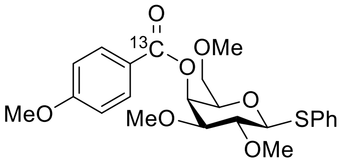

Phenyl 4-*O*-(4-methoxybenzoyl-α-^{13}C)-2,3,6-tri-*O*-methyl-1-thio-β-D-galactopyranoside (7)

To a solution of 4-methoxybenzoic acid-α-^{13}C (41 mg, 0.27 mmol, 2.1 equiv.)[1] in dry CH_3CN (1.3 mL, 0.1M) was added N,N'-carbonyldiimidazole (45 mg, 0.28 mmol, 2.2 quiv) under argon atmosphere. The reaction mixture was stirred at 70 °C for 3h. Thereafter the reaction mixture was cooled to room temperature and phenyl 2,3,6-tri-*O*-methyl-β-D-thiogalactopyranoside (40 mg, 0.13 mmol, 1 equiv)[9] in CH_3CN (1 mL) was added. DBU (40.0 µL, 0.27 mmol, 2.1 equiv) was added and the reaction mixture was stirred at 60 °C overnight for 12h. The reaction mixture was cooled to room temperature and poured in $NaHCO_3$ (sat aq., 5 mL) and extracted using DCM (3 × 10 mL). The combined organic layers were washed with brine (5 mL) and dried over $MgSO_4$. The filtrate was concentrated *in vacuo* and subsequently the crude product was purified using column chromatography (0→20% EtOAc/n-heptane, *v/v*) to obtain phenyl 4-*O*-(4-methoxybenzoyl-α-^{13}C)-2,3,6-tri-*O*-methyl-1-thio-β-D-galactopyranoside (49 mg, 86%) as a clear waxy solid.

TLC: R_f 0.52 (EtOAc/n-heptane, 1:1, *v/v*); **^{1}H NMR** (500 MHz, $CDCl_3$) δ 8.02 – 7.81 (m, 2H), 7.73 – 7.60 (m, 2H), 7.34 (dd, *J* = 5.0, 2.0 Hz, 3H), 6.92 (dd, *J* = 9.0, 0.9 Hz, 2H), 5.70 (t, *J* = 0.9 Hz, 1H, C4-H), 4.57 (d, *J* = 9.5 Hz, 1H, C1-H), 3.89 (s, 3H), 3.80 (td, *J* = 6.1, 1.1 Hz, 1H, C5-H), 3.58 – 3.51 (m, 4H, C6-H), 3.47 – 3.42 (m, 4H, C6-H), 3.39 (dd, *J* = 9.0, 3.2 Hz, 1H, C3-H), 3.35 – 3.27 (m, 4H, C2-H).**^{13}C NMR** (126 MHz, $CDCl_3$) δ 165.5 (C=O), 163.8 (quartenary), 133.2, 132.9 (quarternary), 132.2, 132.2, 128.9, 127.8, 122.1 (d, *J* = 78.3 Hz, quartenary), 113.8, 113.8 (all aromatic), 87.0 (C-1), 84.0 (C-3), 78.2 (C-2), 76.6 (C-5), 71.3 (C-6), 66.8 (d, *J* = 2.6 Hz, C-4), 61.3, 59.6, 57.9, 55.7 (all CH_3). **HRMS:** $[M + Na]^+$ calcd. For $C_{22}{}^{13}CH_{28}O_7S$ 472.1481, found 472.1482.

Computational protocol

The conformational space was explored with the software package CREST[10] version 2.12 using the GFN2-xTB method. The distance matrix for all pairs of conformers (excluding hydrogens) up to 40 kJ/mol from the lowest energy conformation found in CREST was evaluated using the RDKit AllChem.GetBestRMS algorithm. The 60 most unique conformers were subsequently selected by hierarchical clustering of the distance matrix and further optimized using Gaussian 16[11] revision C.02 at PCM(dichloromethane)- B3LYP/6-31++G(d,p) level of theory, followed by a vibrational

analysis at the same level of theory and a single point energy calculation at PCM(dichloromethane)-MP2/6-31++G(d,p) level of theory. Initial guesses for the transition state were made by combining the structure of the reactant and product and manually changing the distances and angles around the C1 atom. The lowest energy conformer based on free energy was further optimized using ORCA 6.0.1[12] at CPCM(dichloromethane)-B3LYPD3/def2-TZVP level of theory and the Gibbs free energy at 213.15 K determined by a frequency analysis. Every stationary point was checked for the absence of imaginary frequencies for energy minima or for the presence of only a single imaginary frequency for transition states. Single point CPCM(dichloromethane)-RI-MP2/aug-cc-pVTZ calculations were performed on the B3LYP-D3 optimized geometries and combined with the thermal correction from the frequency analysis to give the Gibbs free energies.

Supporting NMR data
Glycosylation results

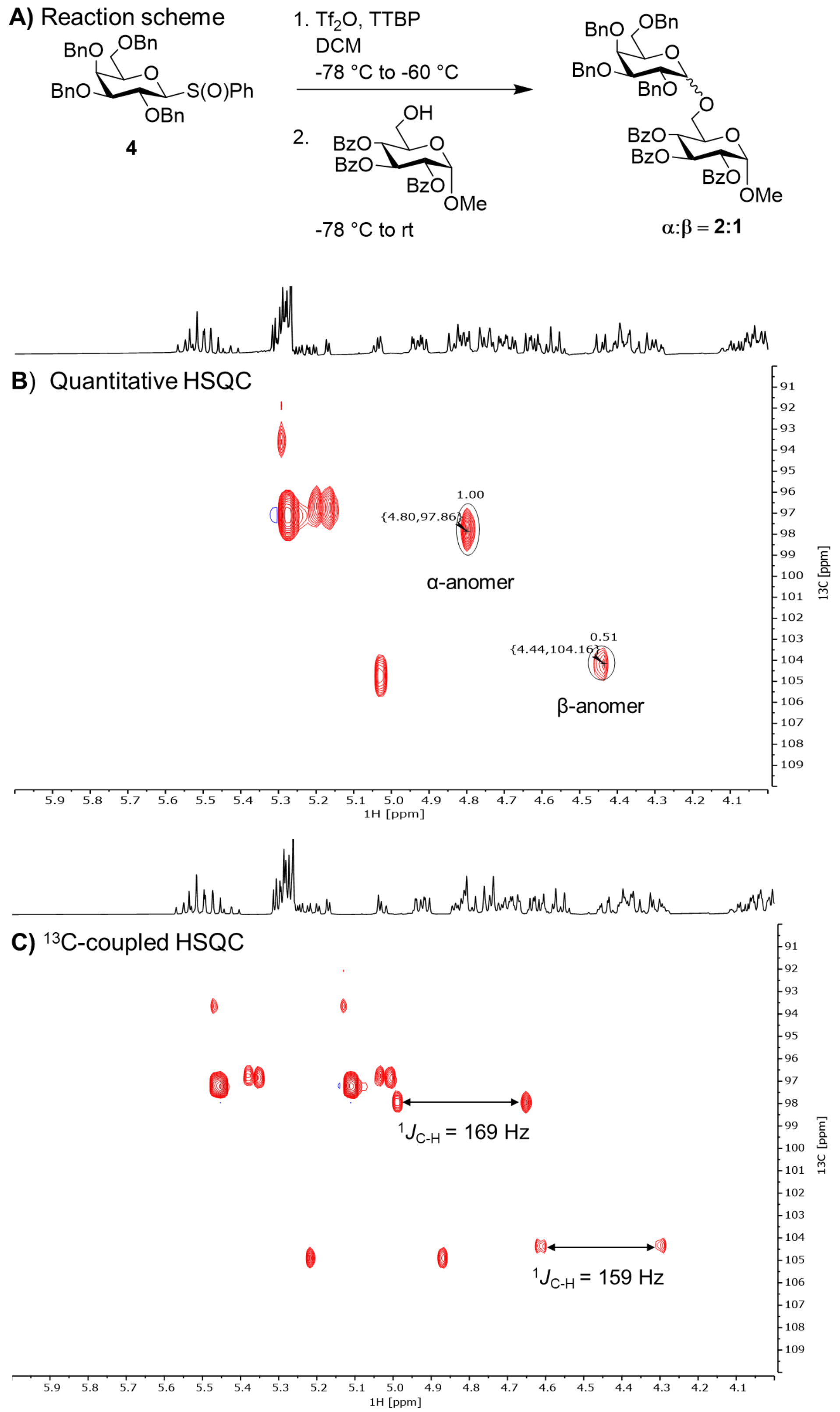

Figure S3: (A) Preactivation-based glycosylation of perbenzylated donor **4** with a primary glucosyl acceptor; (B) α/β-selectivity was determined using quantitative HSQC; (C) Determination of the stereochemistry of glycosylation products by measuring $^{1}J_{C,H}$-couplings.[13] All measurements were performed on crude reaction samples without column purification.

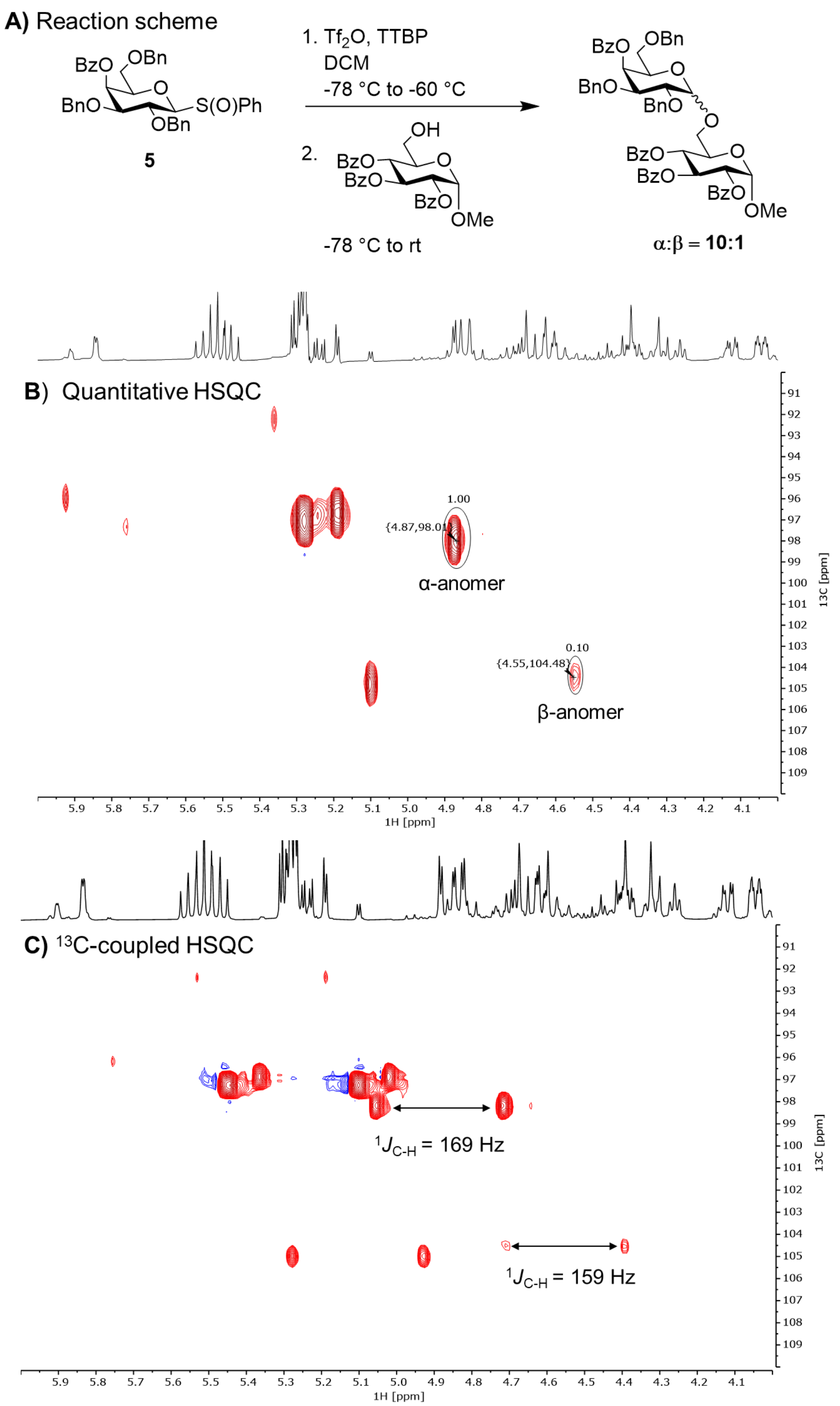

Figure S4: (A) Preactivation-based glycosylation of perbenzylated donor **5** with a primary glucosyl acceptor; (B) α/β-selectivity was determined using quantitative HSQC; (C) Determination of the stereochemistry of glycosylation products by measuring $^{1}J_{C,H}$-couplings.[13] All measurements were performed on crude reaction samples without column purification.

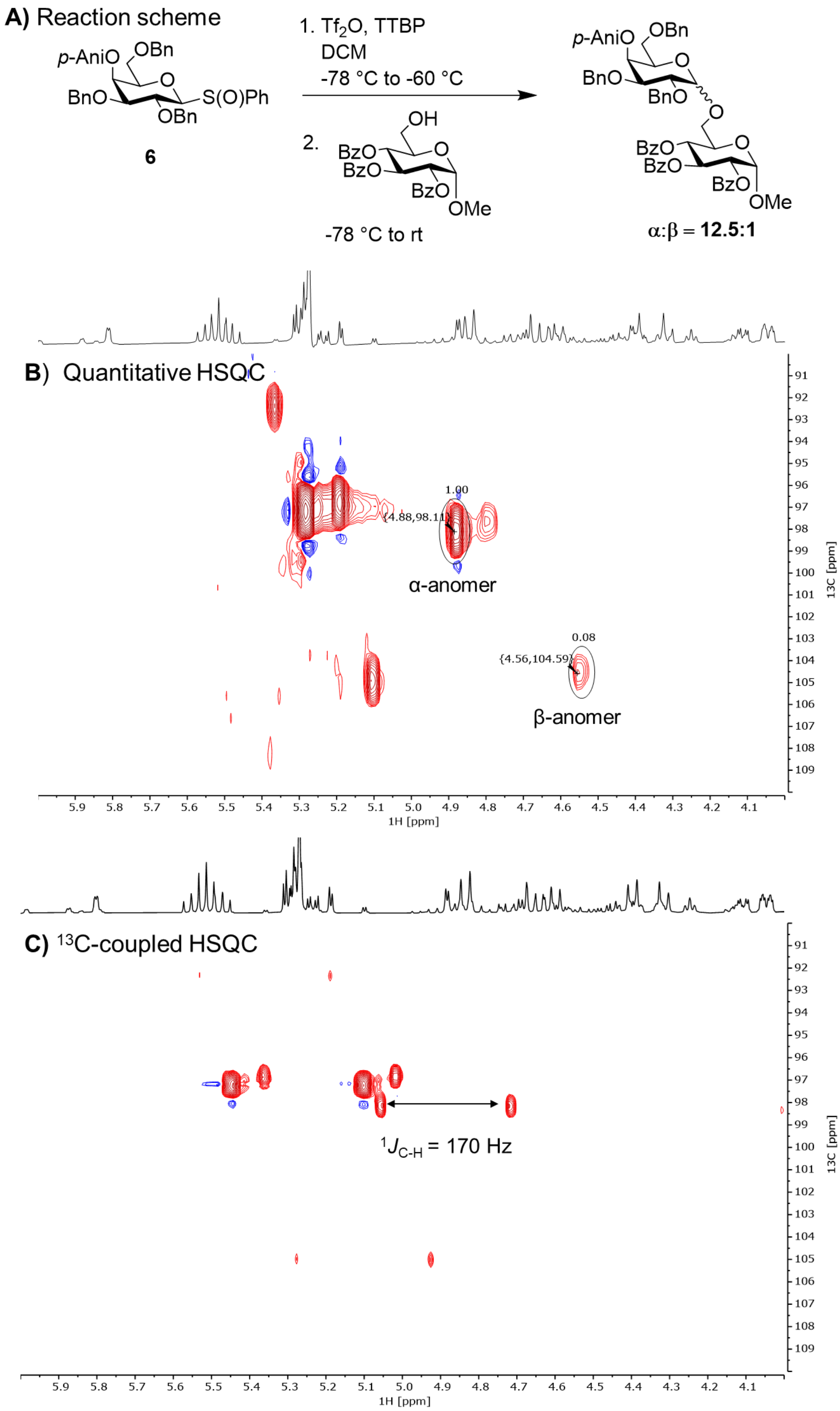

Figure S5: (A) Preactivation-based glycosylation of perbenzylated donor **6** with a primary glucosyl acceptor; (B) α/β-selectivity was determined using quantitative HSQC; (C) Determination of the stereochemistry of glycosylation products by measuring ${}^{1}J_{C,H}$-couplings.[13] Due to the low amount of β-product, the ${}^{1}J_{C,H}$-coupling of the β-anomer could not be determined with reasonable S/N. All measurements were performed on crude reaction samples without column purification.

Supporting NMR data

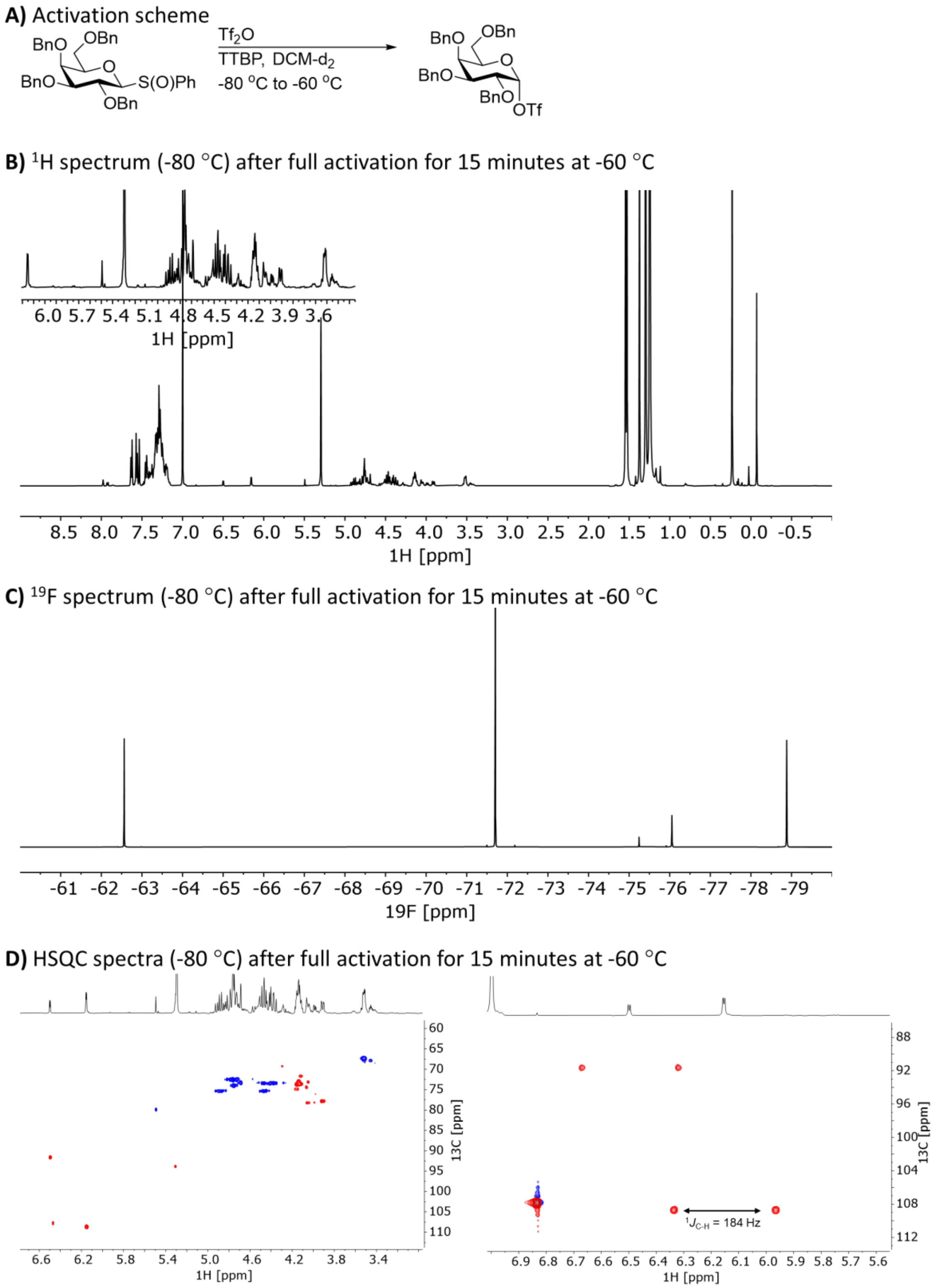

Figure S6: Activation spectra after activation of the C-4 benzyl donor. Activation was conducted at −80 °C using Tf_2O (1.5 eq.) in the presence of TTBP (2.5 eq.), followed by heating to −60 °C for 15 minutes and cooling the sample to −80 °C; (A) Activation scheme including only the main observable triflate as product of activation; (B) ^{1}H spectrum after activation displaying the α-triflate as major intermediate; (C) ^{19}F spectrum after activation; (D) HSQC after activation displaying the α-triflate as major intermediate.

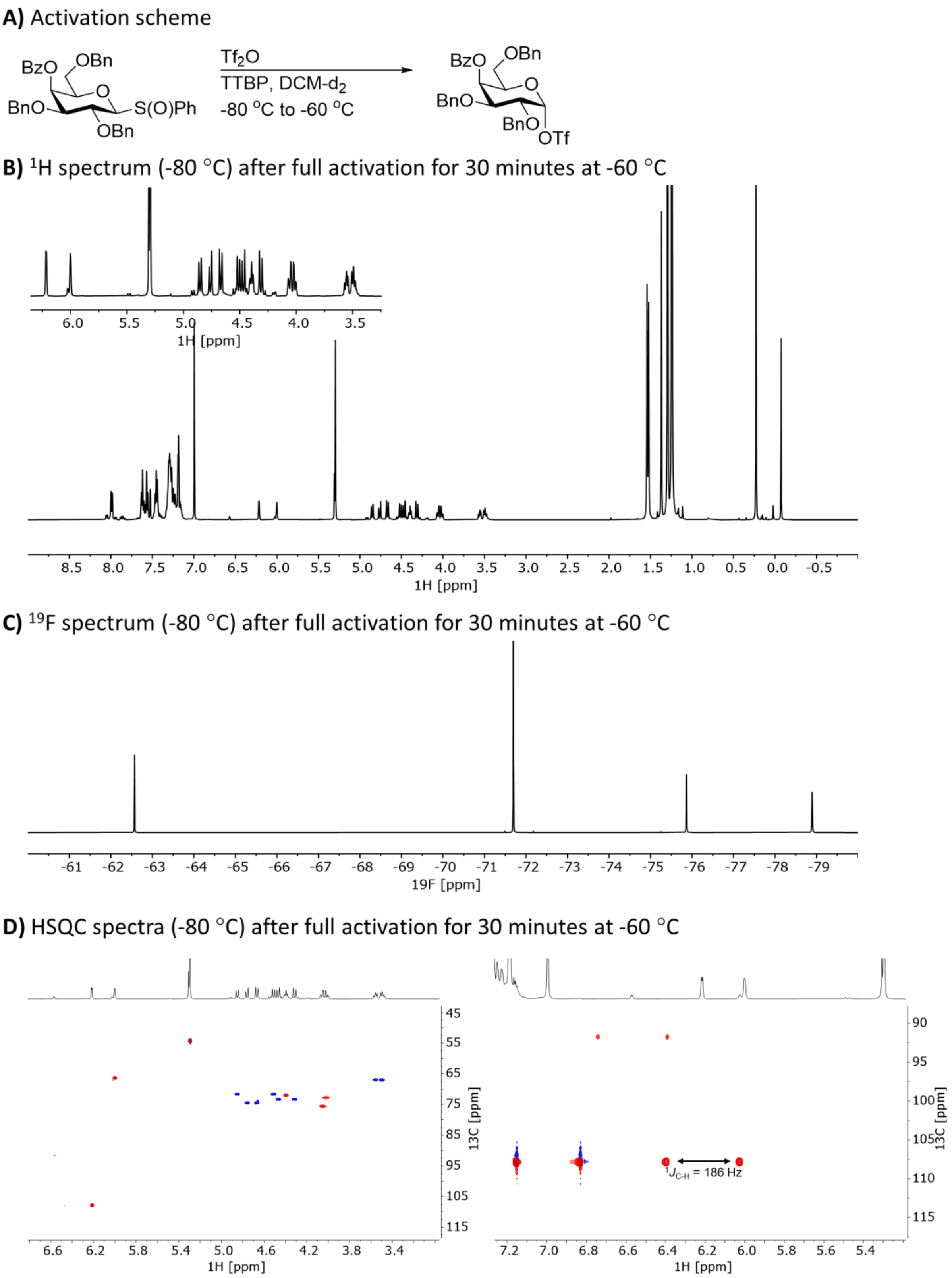

Figure S7: Activation spectra after activation of the C-4 benzoyl donor. Activation was conducted at −80 °C using Tf_2O (1.5 eq.) in the presence of TTBP (2.5 eq.), followed by heating to −60 °C for 30 minutes and cooling the sample to −80 °C; (A) Activation scheme including only the main observable triflate as product of activation; (B) ^{1}H spectrum after activation displaying the α-triflate as major intermediate; (C) ^{19}F spectrum after activation; (D) HSQC after activation displaying the α-triflate as major intermediate and no evidence for dioxepanium ions were observed due to the lack off a cross-peak at δ_H/δ_C = 6.8/110 ppm[14] which would have an expected $^1J_{CH} \approx 200$ Hz.[15]

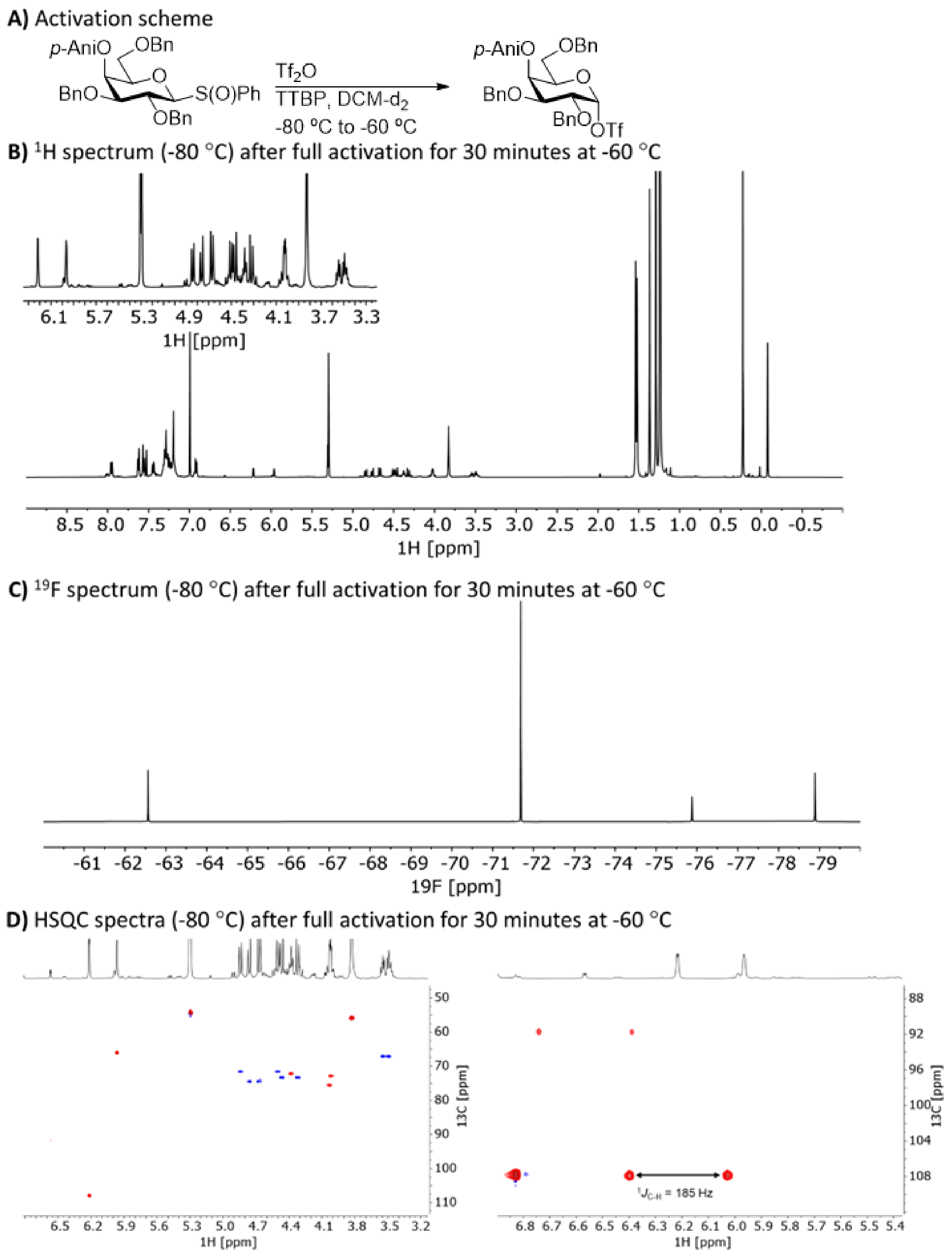

Figure S8: Activation spectra after activation of the C-4 *p*-methoxy-benzoyl donor. Activation was conducted at −80 °C using Tf_2O (1.5 eq.) in the presence of TTBP (2.5 eq.), followed by heating to −60 °C for 30 minutes and cooling the sample to −80 °C; (A) Activation scheme including only the main observable triflate as product of activation, (B) ^1H spectrum after activation displaying the α-triflate as major intermediate; (C) ^{19}F spectrum after activation; (D) HSQC after activation displaying the α-triflate as major intermediate and no evidence for dioxepanium ions were observed due to the lack off a cross-peak at δ_H/δ_C = 6.8/110 ppm[14] which would have an expected $^1J_{CH} \approx 200$ Hz.[15]

A) Activation scheme

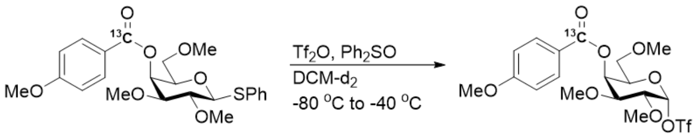

B) ^{1}H spectrum (-80 °C) after full activation for 1h at -40 °C

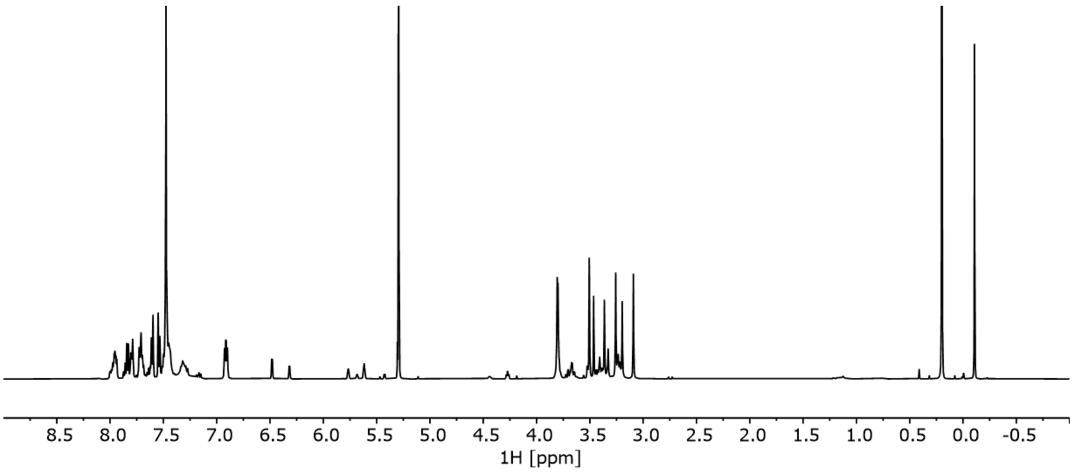

C) ^{19}F spectrum (-80 °C) after full activation for 1h at -40 °C

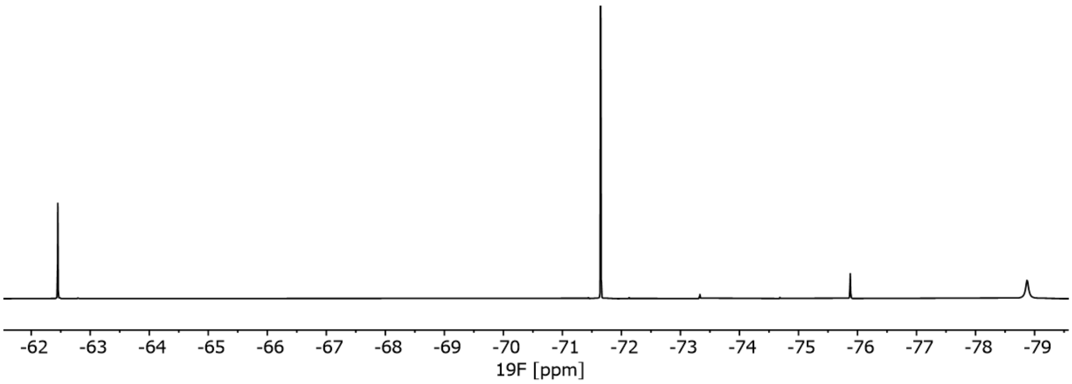

D) HSQC spectra (-80 °C) after full activation for 1h at -40 °C

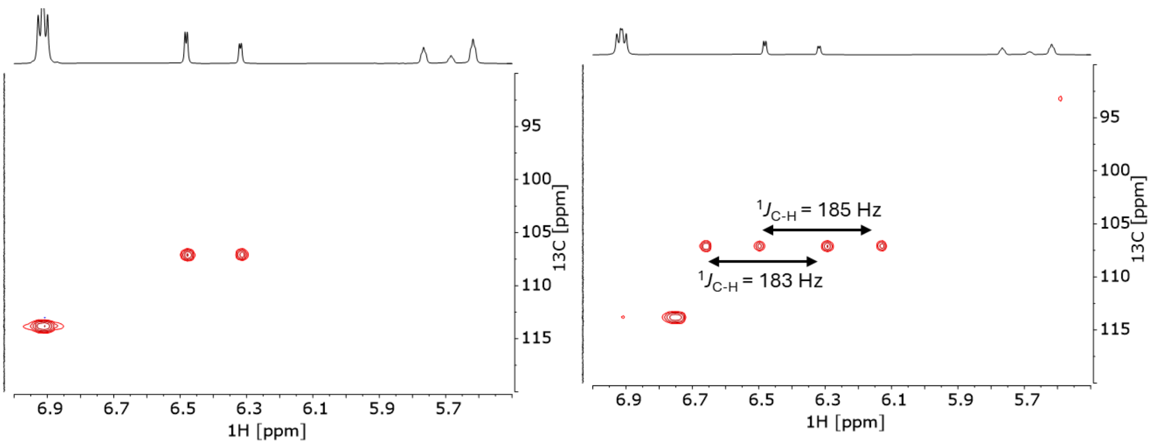

Figure S9: Activation spectra after activation of the C-4 *p*-methoxy-benzoyl donor. Activation was conducted at −80 °C using Tf_2O (1.5 eq.) and Ph_2SO (1.1 eq.) in the absence of TTBP, followed by heating to −40 °C for one hour and cooling the sample to −80 °C; (A) Activation scheme; (B) ^{1}H spectrum after activation displaying the α-triflate as intermediate along with some oxosulfonium ion;[16] (C) ^{19}F spectrum after activation.

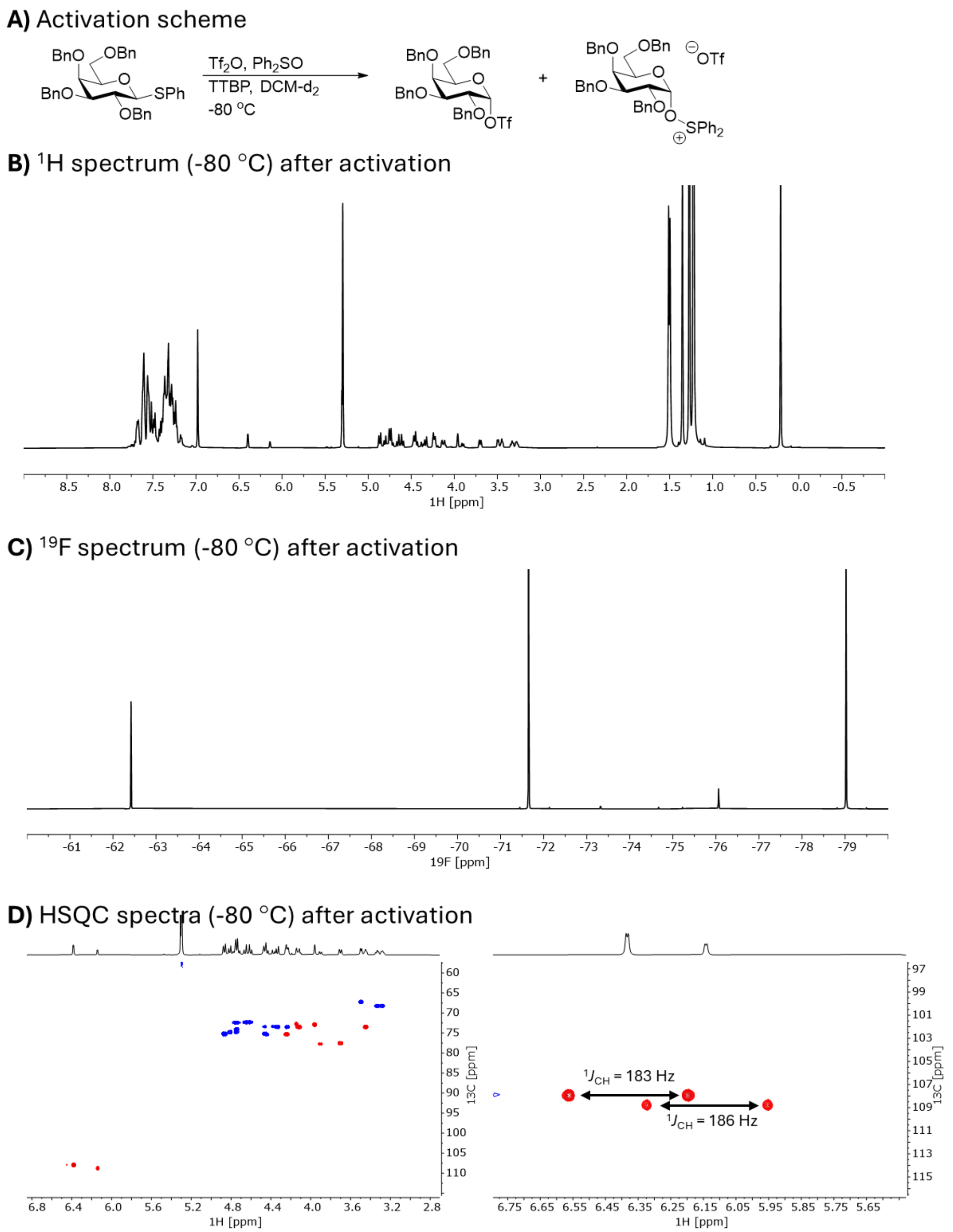

Figure S10: Activation spectra after activation of the C-4 benzyl donor equipped with an anomeric thioether. Activation was conducted at −80 °C using Tf_2O (1.5 eq.) and Ph_2SO (1.1 eq.) in the presence of TTBP (2.5 eq.). (A) Activation scheme including the main observable products of activation. The oxosulfonium ion was assigned upon comparing the characterization spectra (^1H and HSQCs) with literature on oxosulfonium ions; (B) ^1H spectrum after activation displaying the α-triflate as major intermediate; (C) ^{19}F spectrum after activation; (D) HSQC after activation displaying the α-triflate as major intermediate.

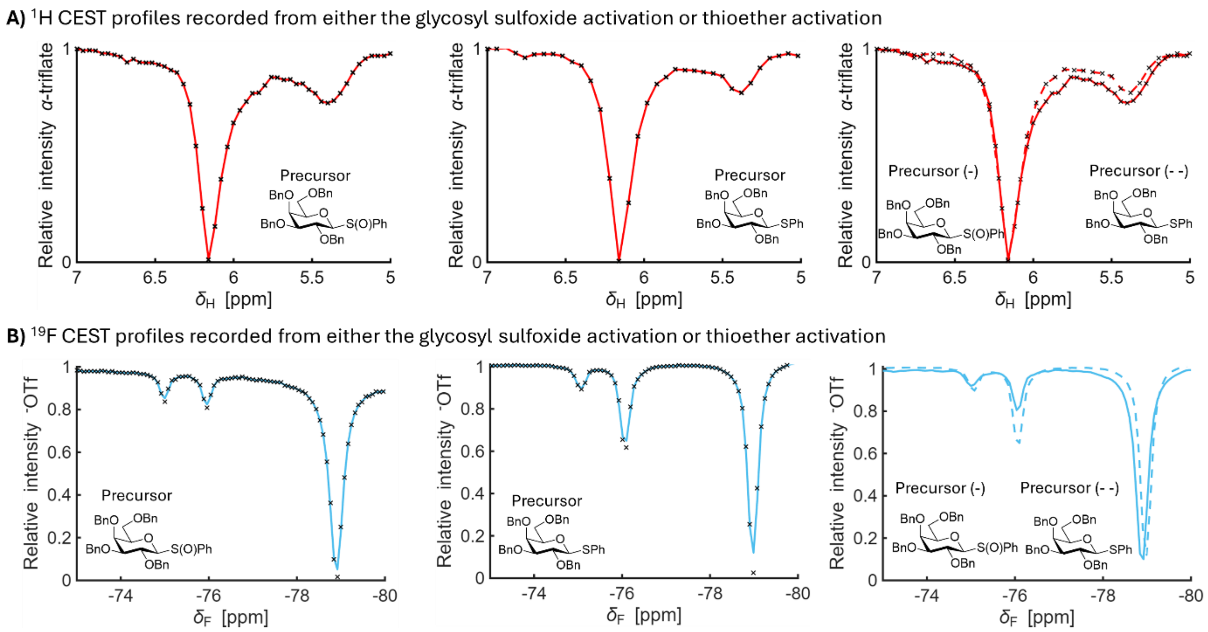

Figure S11: ^{1}H CEST profiles (A) and ^{19}F CEST profiles (B) recorded after activation of the given precursor towards the axial α-triflate. Glycosyl sulfoxide activation was achieved by mixing the glycosyl sulfoxide in DCM-d_2 in the presence of TTBP (2.5 eq.) in an NMR tube, cool to -80 °C, and active with Tf_2O (1.5 eq.). Subsequent heating in the probe to -60 °C resulted in the formation of the axial α-triflate. The sample was cooled to -80 °C and CEST profiles were recorded. The thioether precursor was activated using Ph_2SO (1.1 eq.) and Tf_2O (1.5 eq.) in the presence of TTBP (2.5 eq.). Activation was conducted at -80 °C and CEST profiles could directly be obtained.

Discussion on Figure S11: In ^{1}H CEST NMR the anomeric proton was taken as read-out signal to construct the CEST profile whilst in ^{19}F CEST the unbound triflate ($^{-}$OTf) resonance was used. The ^{1}H CEST spectra indicates that the axial α-triflate is in exchange with a species upfield from it. This has been assigned to be the equatorial β-triflate and as the two individual activation methods yield the same CEST profile it is likely that this species is present in both cases thus further supporting an equatorial β-triflate. Other intermediates resulting from the activation method could also be considered however it is unlikely that different intermediates would provide such identical chemical shift for the minor species. The ^{19}F CEST profile required the $^{-}$OTf resonance as read-out and displays two species to be in chemical exchange with it. The most upfield one is assigned to be the axial a-triflate as this is clearly visible in 1D ^{19}F spectroscopy. The more downfield one is assigned to be the equatorial β-triflate. The axial α-triflate is not ideal as read-out for ^{19}F CEST because saturation from the equatorial β-triflate requires multiple steps to become visible on the axial α-triflate and once the non-magnetized $^{-}$OTf (which became unbound due to axial α-triflate formation) its population relative to magnetized $^{-}$OTf is statistically unfavoured it becomes particularly difficult to detect by taking the axial α-triflate resonance as read-out (Figure S12).

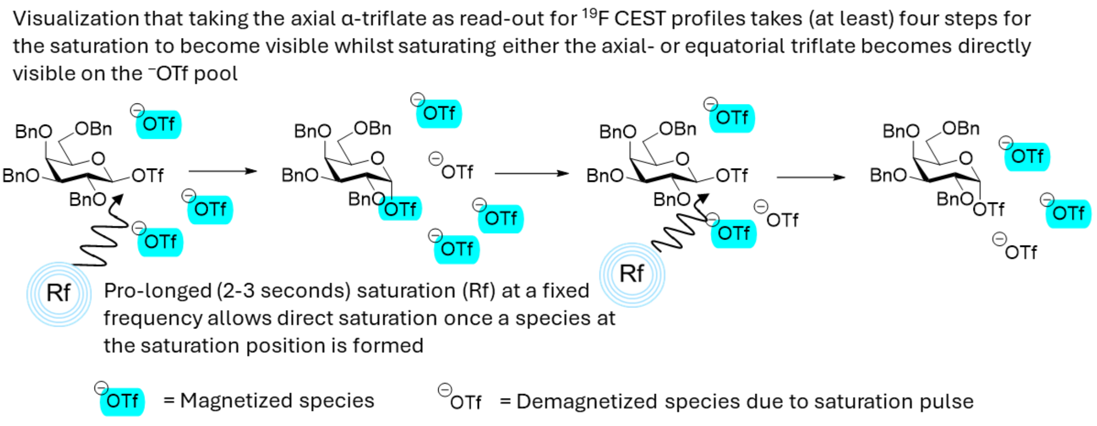

Figure S12: ^{19}F CEST read-out signal comparison.

Phenyl 4-*O*-(4-methoxybenzoyl-α-^{13}C)-2,3,6-tri-*O*-methyl-1-thio-β-D-galactopyranoside variable temperature NMR experiment

Phenyl 4-*O*-(4-methoxybenzoyl-α-^{13}C)-2,3,6-tri-*O*-methyl-1-thio-β-D-galactopyranoside was studied to force C-4 acyl participation. Activation of the donor with Tf_2O and Ph_2SO in the absence of TTBP was found to be the most C-4 acyl NGP forcing solution phase conditions (without substituting H-4 for a methyl group[14, 17]). Under these conditions the galactosyl α-triflate was formed along with α-oxosulfonium ion.[16] No evidence for the 1,4-bridged dioxepanium ion was found based on 1D ^{1}H and ^{13}C experiments or 2D ^{1}H/^{13}C HMBC and HSQC experiments. The triflate dissociation rate was measured using EXSY ($R_{\alpha\rightarrow OTf,\ EXSY}$) at varying temperatures and with fixed temperature (-80 °C) with varying concentrations tetrabutyl ammonium triflate (Bu_4NOTf). Additionally, ^{1}H CEST, ^{19}F CEST, and ^{13}C CEST spectra were recorded. All these experiments support a mechanism where C-4 acyl NGP is not occurring under the reaction conditions hence excluding this mechanism as explanation for the observed α-selectivity.

A) Activation scheme

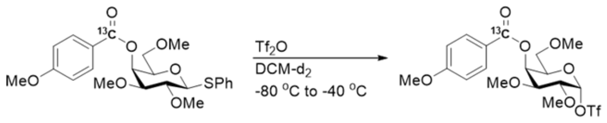

B) ^{1}H/^{13}C HMBC of the activated glycosyl donor at -80 °C

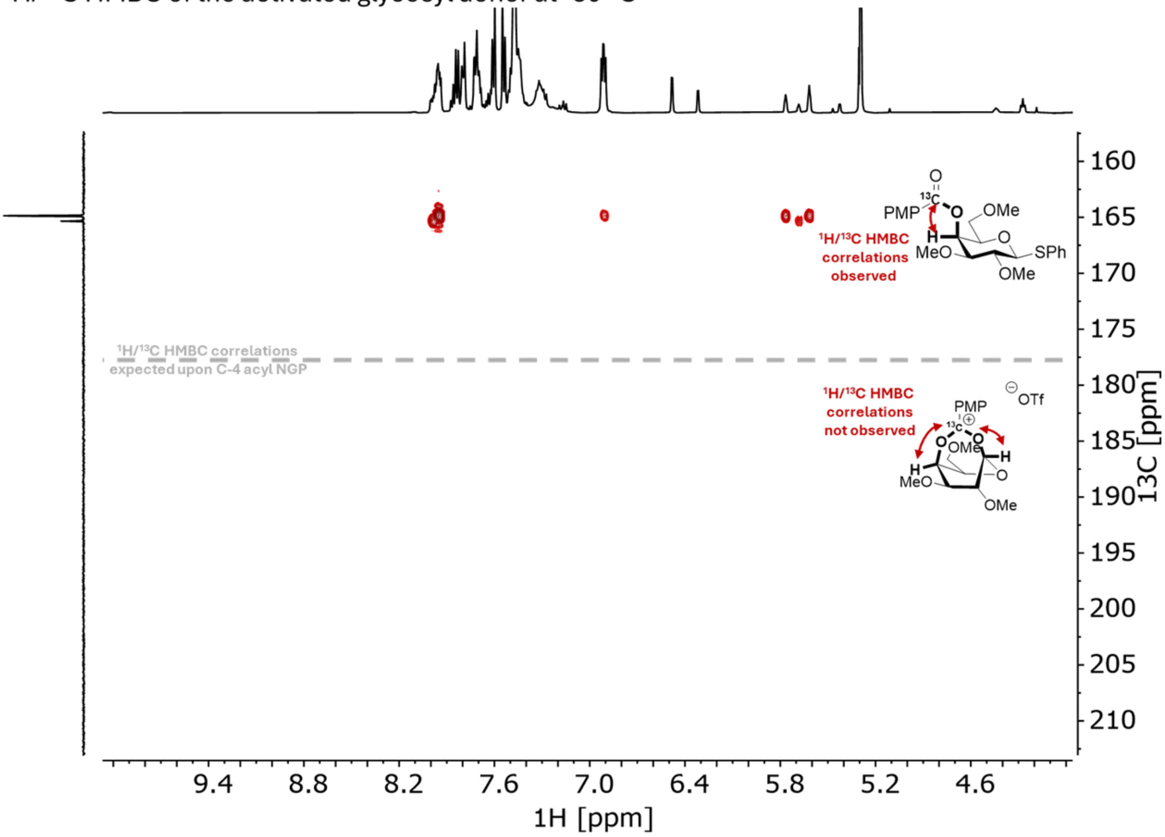

Figure S13: ^{1}H/^{13}C HMBC spectrum recorded at -80 °C on the activated galactosyl donor solution. The experiments does not display evidence of 1,4-bridged dioxepanium ion formation due to absence of some expected cross-peaks at $\delta_C \approx 177$ ppm and $\delta_H \approx 6.6$ and 5.8 ppm.

A) ^{1}H CEST profile at -80 °C

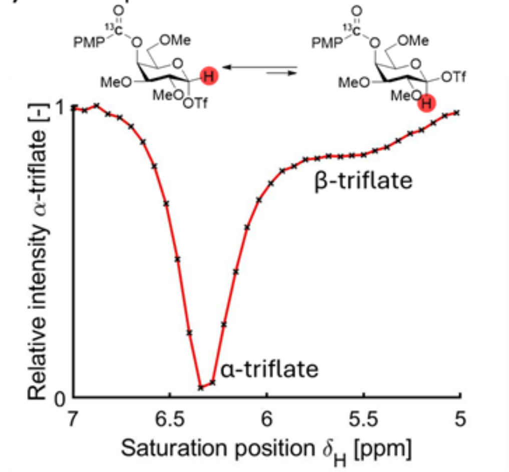

Explanations:
The major dip at δ_H = 6.3 ppm is due to direct saturation of the α-triflate resonance

The minor dip at δ_H = 5.6 ppm is due to saturation of the β-triflate and as this is in chemical exchange with the α-triflate a CEST dip is observed in the CEST profile due to saturation transfer to the main observable species.

B) ^{19}F CEST profile at -80 °C

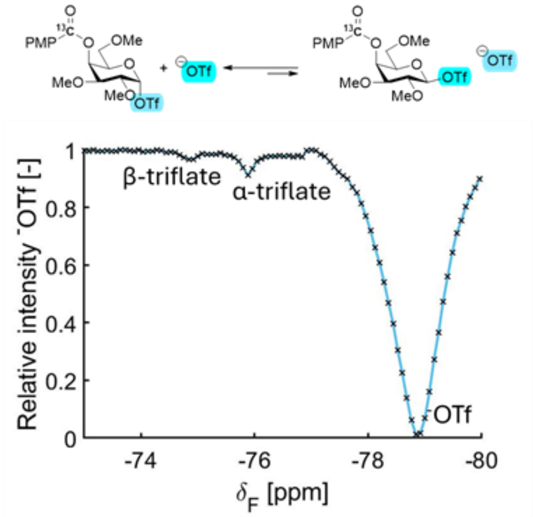

Explanations:
The major dip at δ_F = -79 ppm is due to direct saturation of the ⁻OTf resonance

The minor dip at δ_F = -74.8 ppm is due to saturation of the β-triflate passed to the ⁻OTf resonance as saturated ⁻OTf is released upon α-face attack of another ⁻OTf species.

The minor dip at δ_F = -75.9 ppm is due to saturation of the α-triflate passed to the ⁻OTf resonance as saturated ⁻OTf is released upon β-face attack of another ⁻OTf species.

C) ^{13}C CEST profile at -80 °C

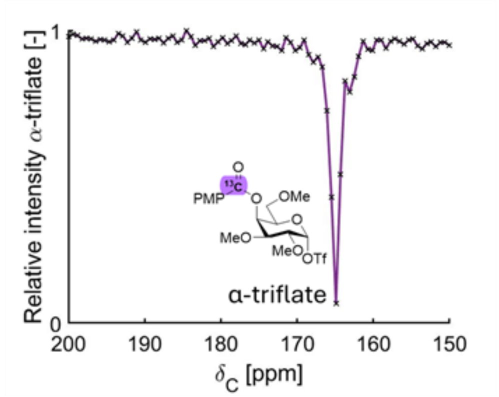

Explanations:
No additional dips are observed in the ^{13}C CEST profile apart from direct saturation of the ^{13}C carbonyl at the α-triflate. No β-triflate (or a weak signal at δ_C = 163.1 ppm) is observed since the carbonyl resonances are very similar due to its remoteness from the anomeric centre. More notably, with 1,4-dioxepanium ions a cationic ^{13}C 'carbonyl' resonance would be expected around δ_C = 180 ppm which is not observed suggesting absence of the species (or detection outside the CEST window). Since multiple experiments point to the absence of the bridging species, likely this intermediate is not formed.

Figure S14: ^{1}H,^{19}F, and ^{13}C CEST profiles (A, B, and C respectively). In the ^{1}H and ^{13}C profiles, the α-triflate resonance was used as a read-out. In ^{19}F CEST the ⁻OTf resonance was used as main observable read-out signal. The ^{1}H and ^{19}F CEST profiles display evidence of β-triflates being in exchange with the α-triflate. In both ^{1}H and ^{13}C CEST no evidence for a 1,4-bridged dioxepanium ion was observed due to absence of resonances at $\delta_H \approx 6.6$ and $\delta_C \approx 185$ ppm respectively.

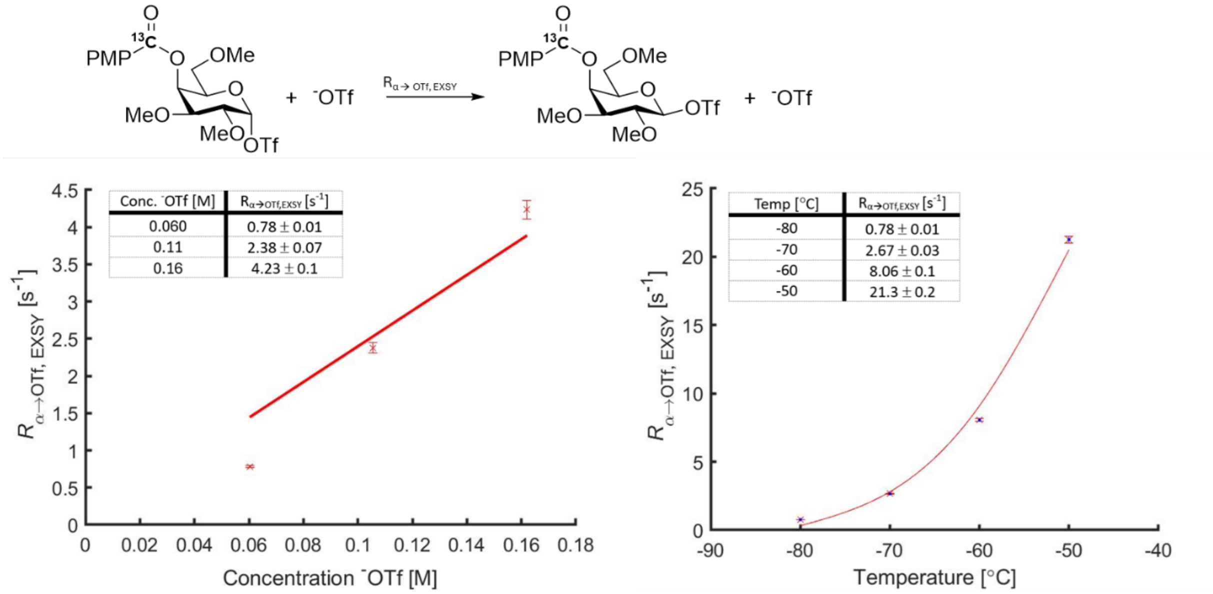

Figure S15: ^{19}F EXSY experiments to determine the rate of α-triflate dissociation at −80 °C at varying temperatures demonstrating linear increase of the rate as the concentration $^{-}$OTf increases which suggests a S_N2 exchange process. Triflate dissociation was also measured at varying temperatures displaying the same trend observed where increasing the temperature increases the triflate dissociation rate.

CEST fitting results

Single data set multiparameter fitting results

A) Exchange scheme

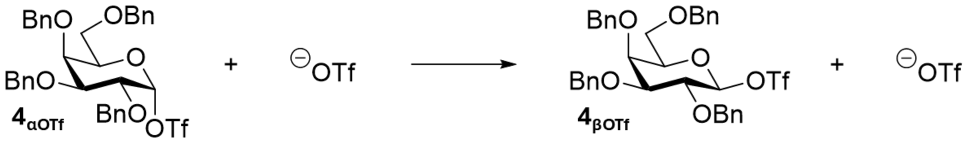

B) Fitting outcome with a β-population of 0.2% and β- to α-triflate exchange rate of 200 s^{-1}

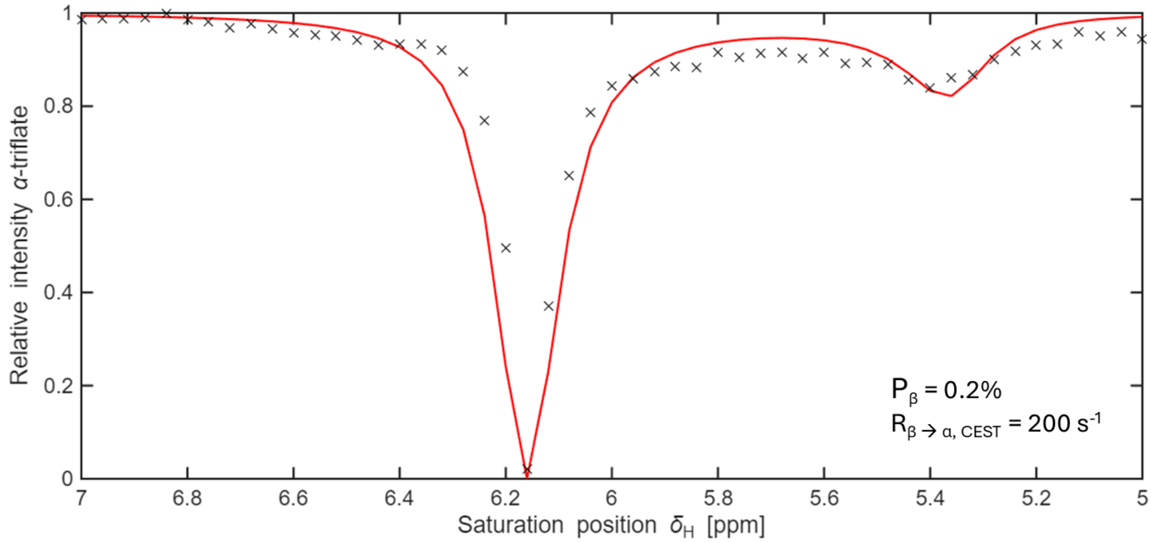

C) Fitting outcome with a β-population of 1.3% and β- to α-triflate exchange rate of 20 s^{-1}

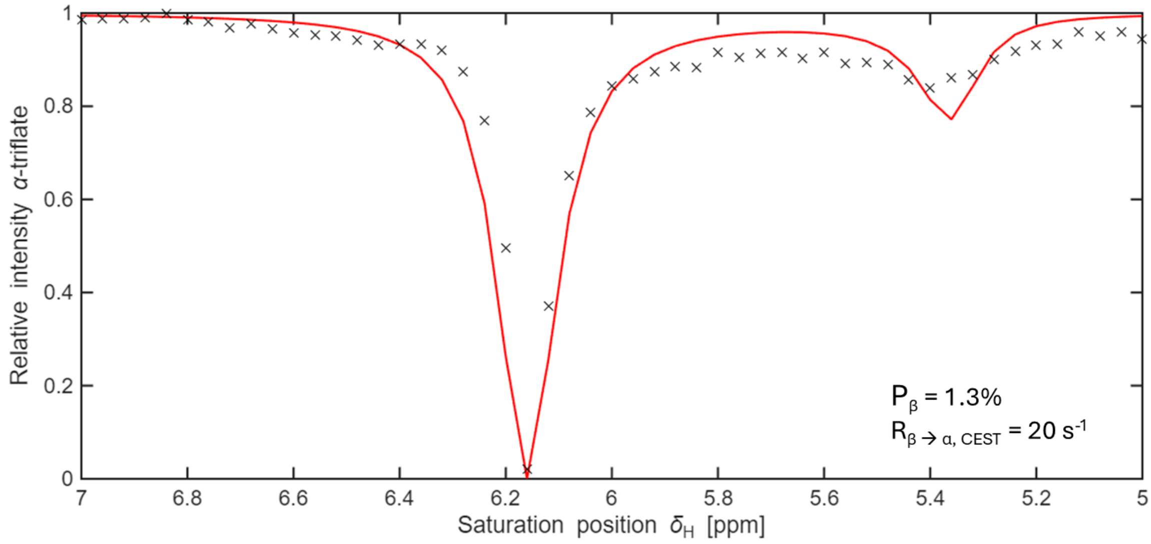

Figure S16: A) Exchange scheme for the initial fitting; B) Fitting results with a β-population of 0.2% and rate of β- to α-triflate exchange of 200 s^{-1} yielding a good fit; C) Fitting results with a β-population of 1.3% and rate of β- to α-triflate exchange of 20 s^{-1} yielding a good fit.

Multiple data set multiparameter fitting results

A) Overall fitting, least squares fit combining the CEST profiles over different frequencies to one set of solutions

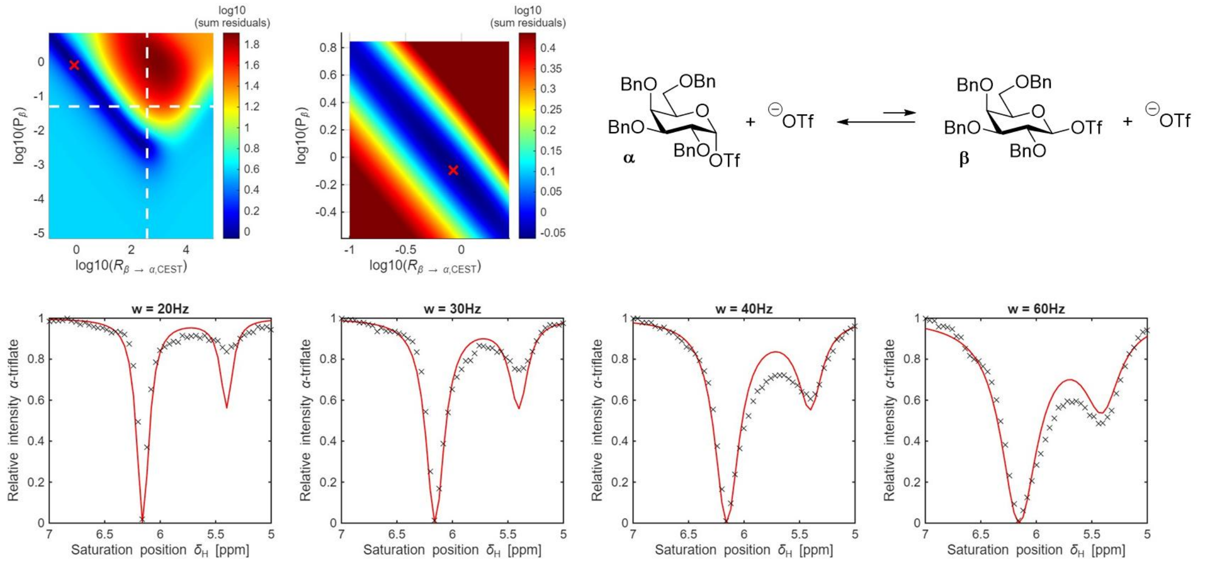

B) Individual fitting, least squares fit taking only one frequency into account per fit

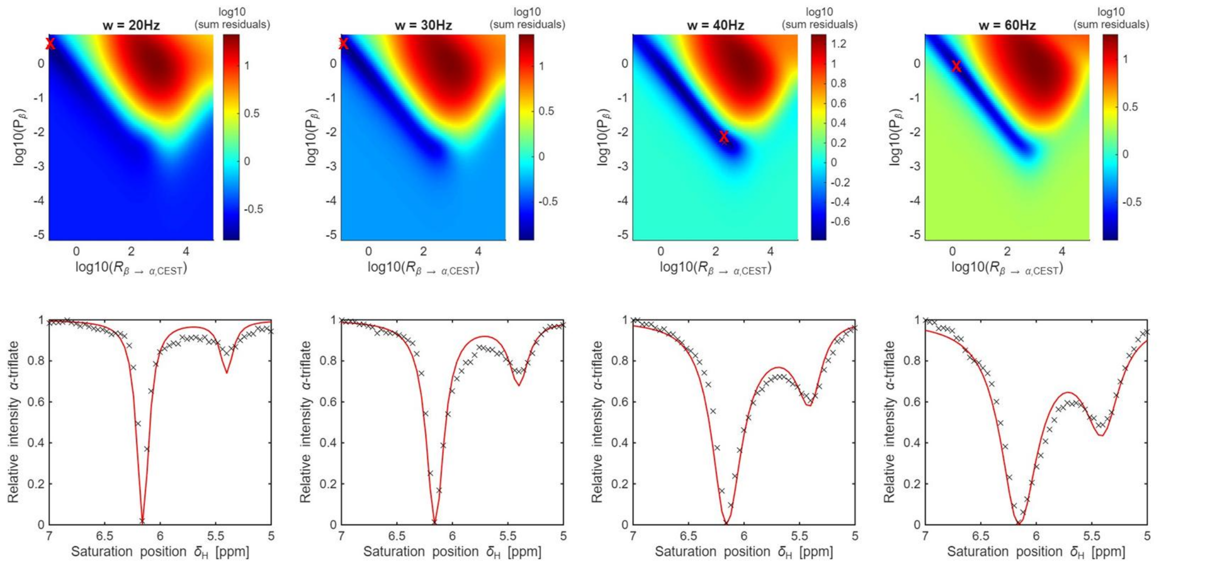

Figure S17: (A) Overall CEST fitting results highlighting a broad area of solutions in order to provide reasonable fits. In overall CEST fitting, four CEST profiles recorded at different saturation field strengths (ϖ = 20, 30, 40, or 60 Hz) were simulated to the values of $R_{\alpha \rightarrow \beta,\ CEST}$ and $R_{\beta \rightarrow \alpha,\ CEST}$ that best fit the experimental data; (B) Individual CEST fitting resulted in a variety of different solutions within the blue area of part A. This highlights where blue area in the heatmap in figure A comes from. Individual fitting, CEST profile fitting at different saturation field strengths to their individual best fits for $R_{\alpha \rightarrow \beta,\ CEST}$ and $R_{\beta \rightarrow \alpha,\ CEST}$.

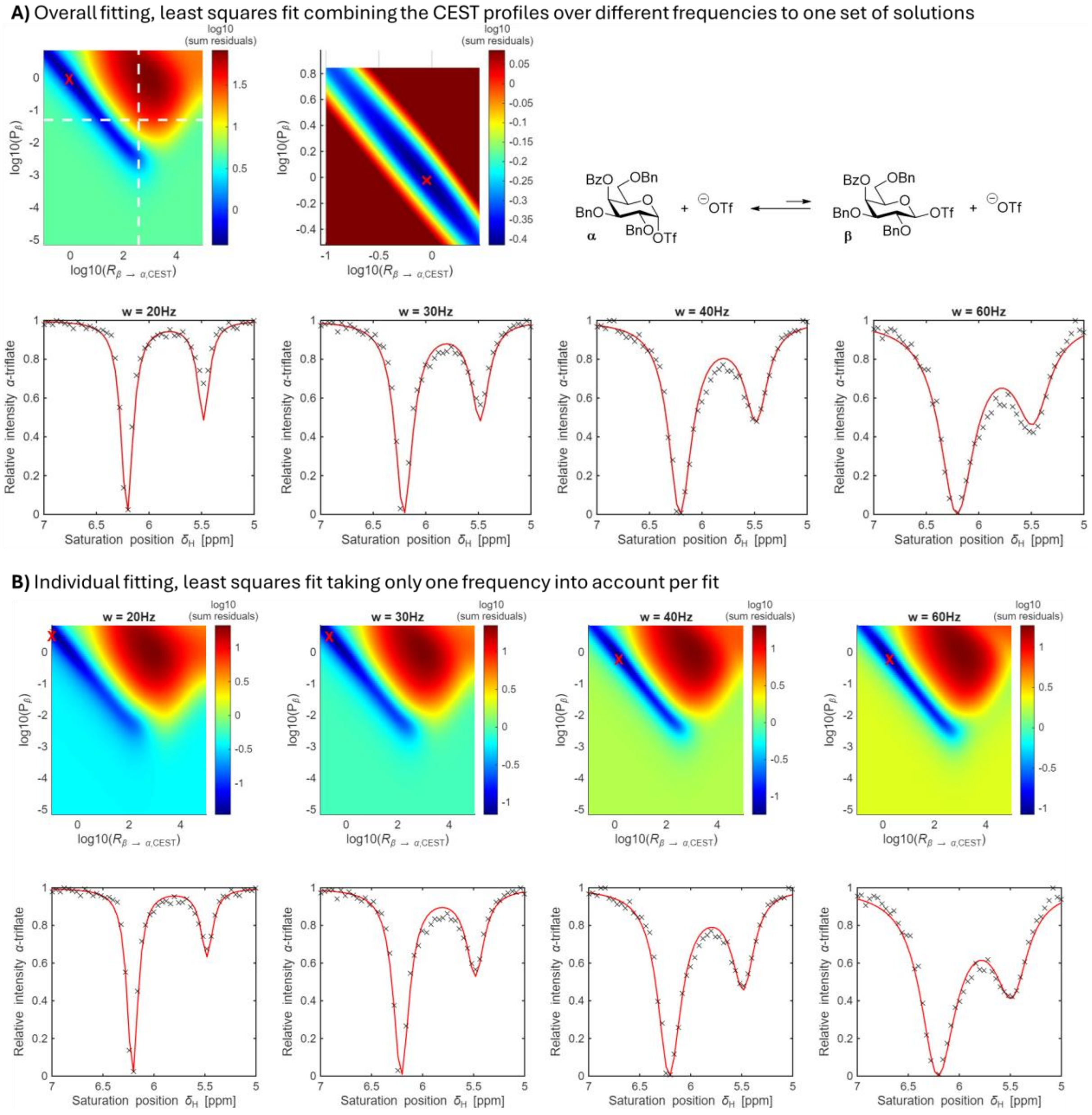

Figure S18: (A) Overall CEST fitting results highlighting a broad area of solutions to provide reasonable fits. In overall CEST fitting, four CEST profiles recorded at different saturation field strengths (ϖ = 20, 30, 40, or 60 Hz) were simulated to the values of $R_{\alpha \rightarrow \beta, \text{CEST}}$ and $R_{\beta \rightarrow \alpha, \text{CEST}}$ that best fit the experimental data (B) Individual CEST fitting results in a variety of different solutions within the blue area of part A. This highlights where blue area in the heatmap in figure A comes from. Individual fitting, CEST profile fitting at different saturation field strengths to their individual best fits for $R_{\alpha \rightarrow \beta, \text{CEST}}$ and $R_{\beta \rightarrow \alpha, \text{CEST}}$.

A) Overall fitting, least squares fit combining the CEST profiles over different frequencies to one set of solutions

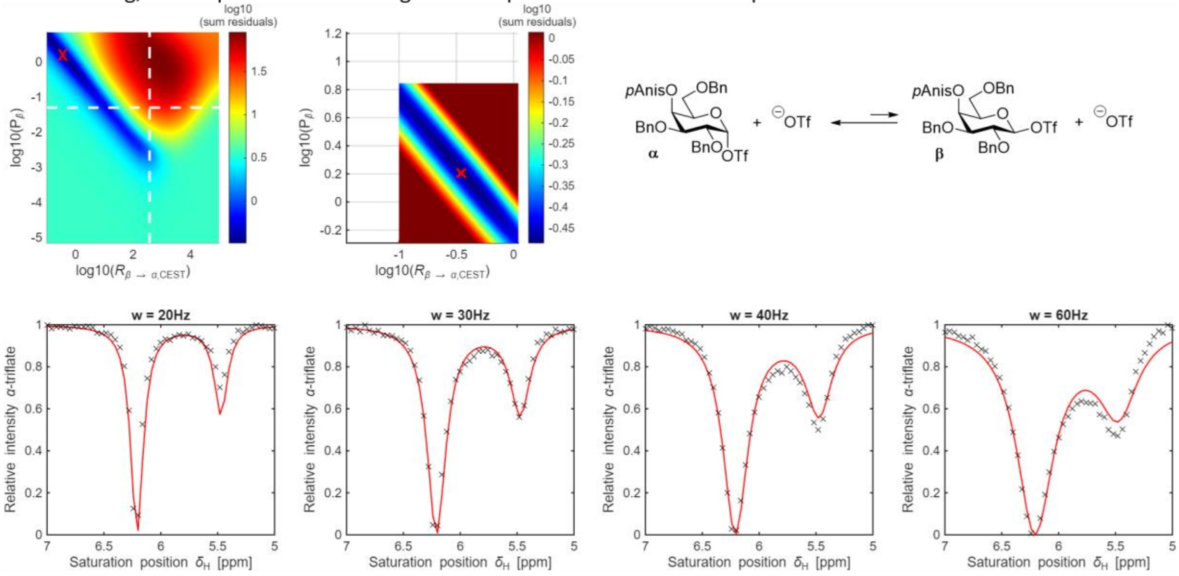

B) Individual fitting, least squares fit taking only one frequency into account per fit

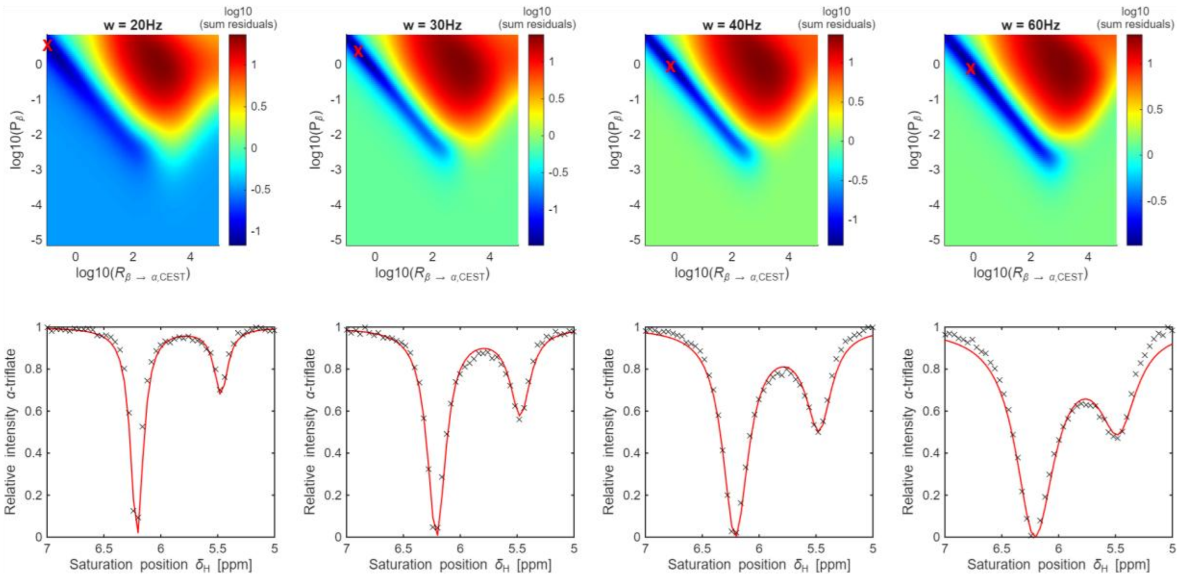

Figure S19: (A) Overall CEST fitting results highlighting a broad area of solutions to provide reasonable fits. In overall CEST fitting, four CEST profiles recorded at different saturation field strengths (ϖ = 20, 30, 40, or 60 Hz) were simulated to the values of $R_{\alpha \rightarrow \beta, CEST}$ and $R_{\beta \rightarrow \alpha, CEST}$ that best fit the experimental data; (B) Individual CEST fitting results in a variety of different solutions within the blue area of part A. This highlights where blue area in the heatmap in figure A comes from. Individual fitting, CEST profile fitting at different saturation field strengths to their individual best fits for $R_{\alpha \rightarrow \beta, CEST}$ and $R_{\beta \rightarrow \alpha, CEST}$.

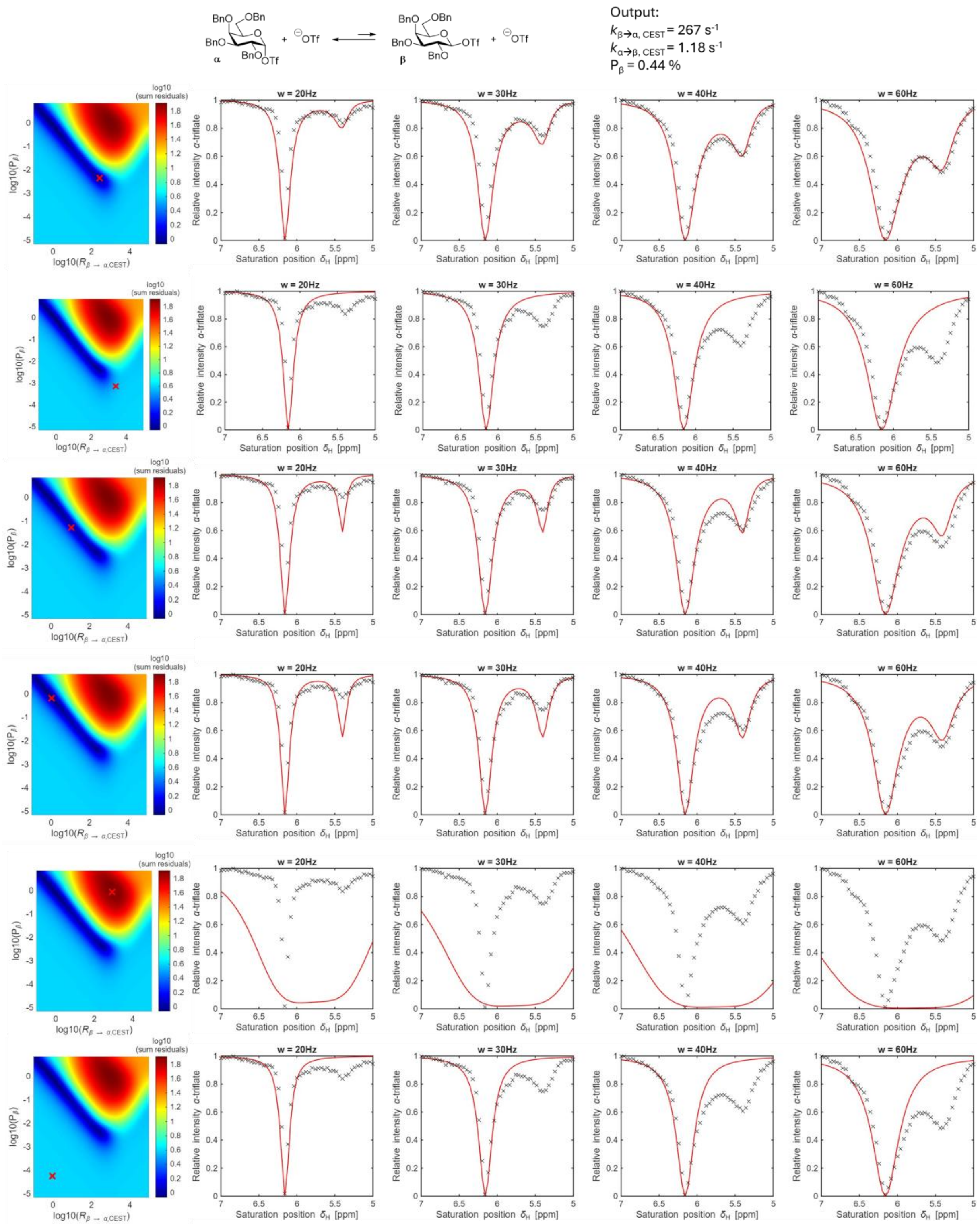

Figure S20: CEST fitting selected highlights (red cross combination in the heat-map). Within the blue area from Figure S11 selected fitting spectra were generated to demonstrate its deviations from the experimental error. Areas in light blue and red clearly indicate poor fitting results. Going from the top left in the blue area to the middle centre demonstrates better peak position and often slightly at the cost of additional errors resulting from broadening peak. Overall the best fit selected for the manuscript is the top line.

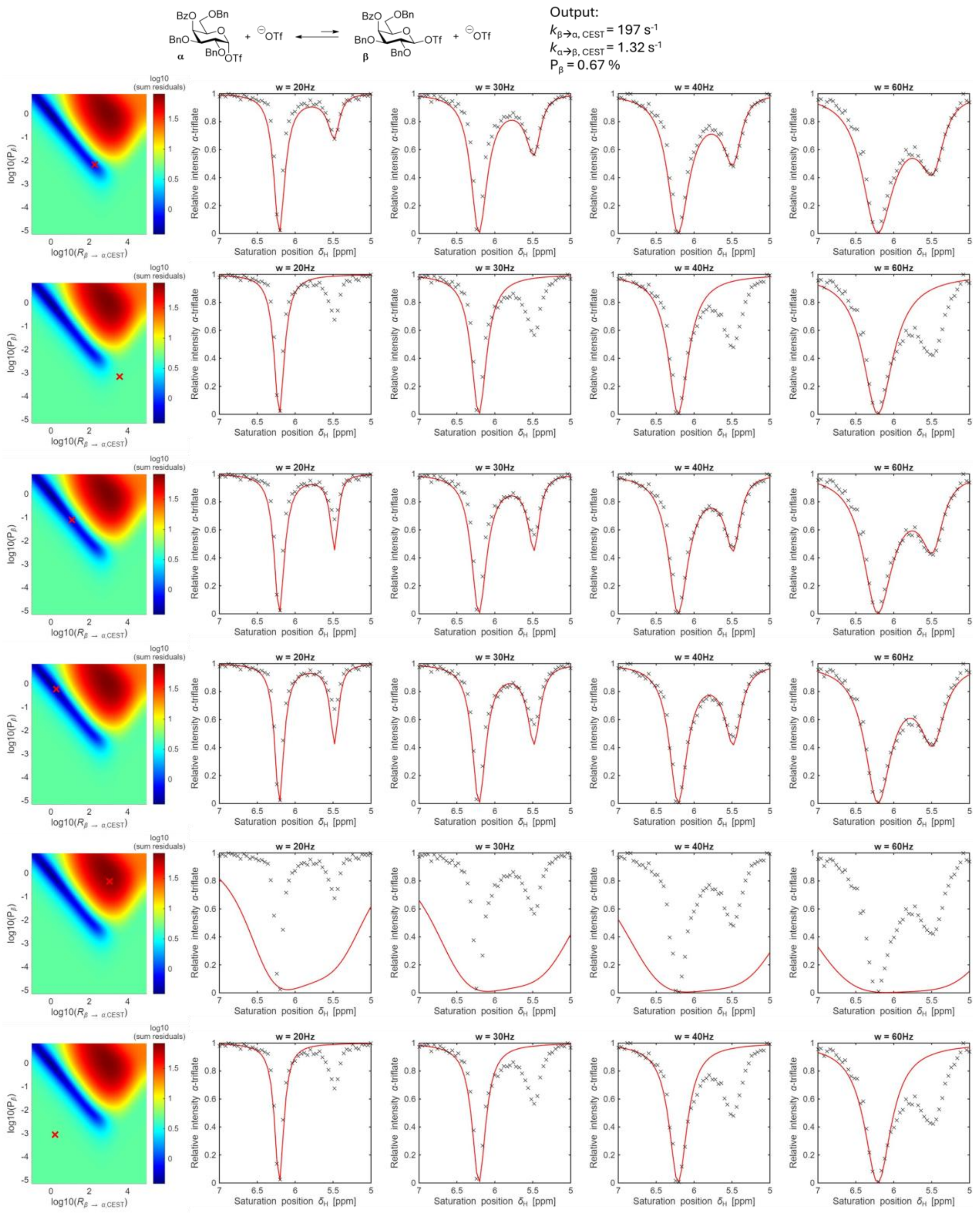

Figure S21: CEST fitting selected highlights (red cross combination in the heat-map). Within the blue area from Figure S12 selected fitting spectra were generated to demonstrate its deviations from the experimental error. Areas in light blue and red clearly indicate poor fitting results. Going from the top left in the blue area to the middle centre demonstrates better peak position and often slightly at the cost of additional errors resulting from broadening peak. Overall the best fit selected for the manuscript is the top line.

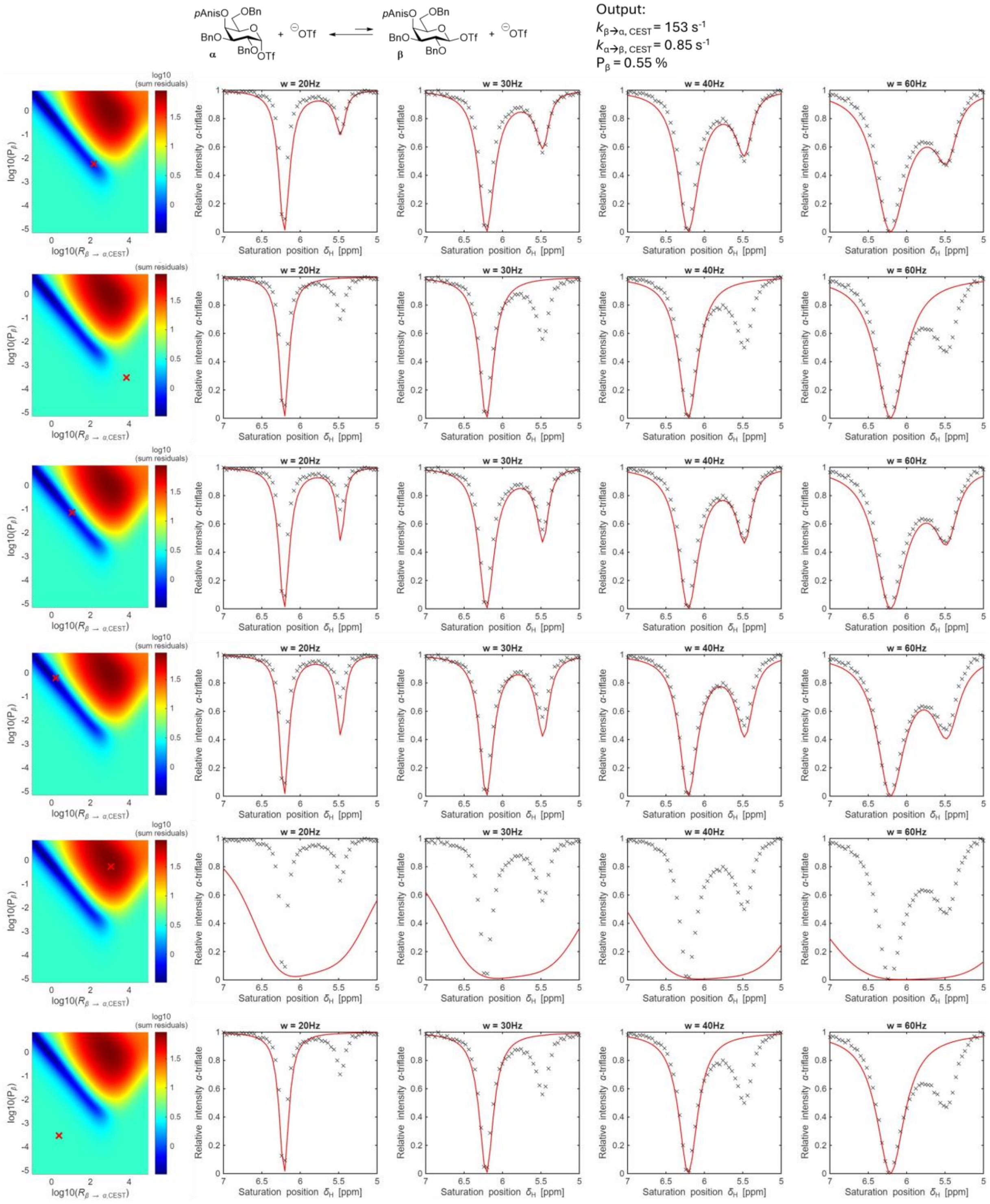

Figure S22: CEST fitting selected highlights (red cross combination in the heat-map). Within the blue area from Figure S13 selected fitting spectra were generated to demonstrate its deviations from the experimental error. Areas in light blue and red clearly indicate poor fitting results. Going from the top left in the blue area to the middle centre demonstrates better peak position and often slightly at the cost of additional errors resulting from broadening peak. Overall the best fit selected for the manuscript is the top line.

Single parameter fitting results

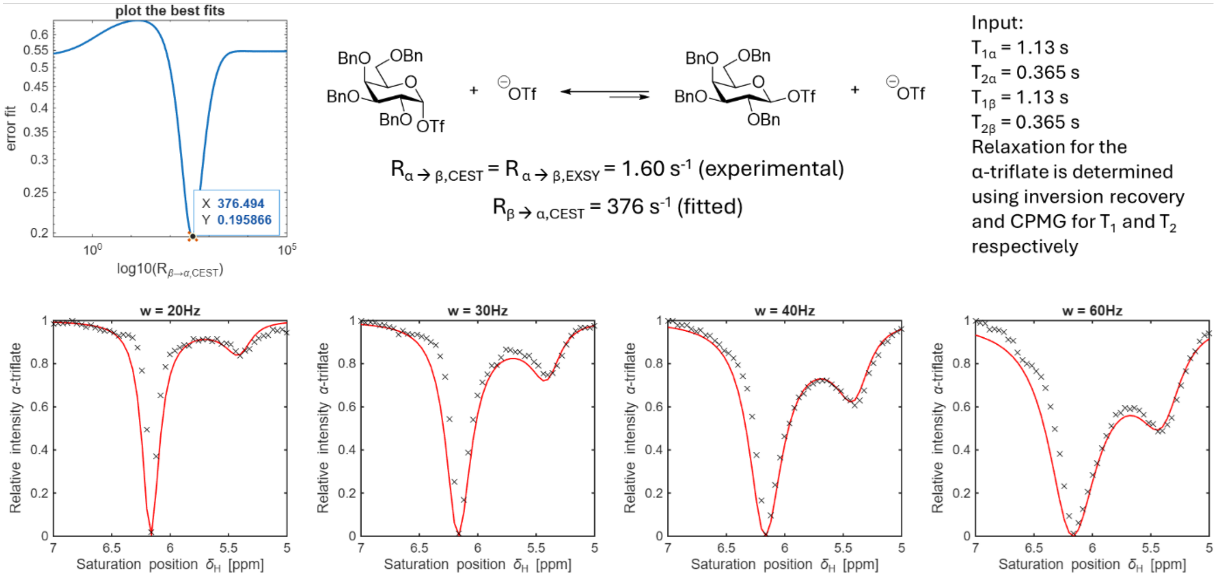

Figure S23: Single parameter fit outcome where the $R_{\alpha\rightarrow\beta,\ CEST}$ was fixed to the value of triflate dissociation found using ^{19}F EXSY ($R_{\alpha\rightarrow OTf,\ EXSY}$) and T_1 and T_2 for the axial α-triflate was measured using inversion recovery and CPMG respectively. The relaxation rates for the equatorial β-triflate were assumed to be the same as the axial α-triflate. The $R_{\beta\rightarrow\alpha,\ CEST}$ was determined by the Bloch-McConnel fitting to the CEST data measured at saturation field strengths of 20, 30, 40, and 60 Hz. The error at each simulation for $R_{\beta\rightarrow\alpha,\ CEST}$ was plotted against the rate which resulted in the best fits at the given $R_{\beta\rightarrow\alpha,\ CEST}$. This was in good agreement with the multi-parameter fit where both $R_{\beta\rightarrow\alpha,\ CEST}$ and $R_{\alpha\rightarrow\beta,\ CEST}$ were determined.

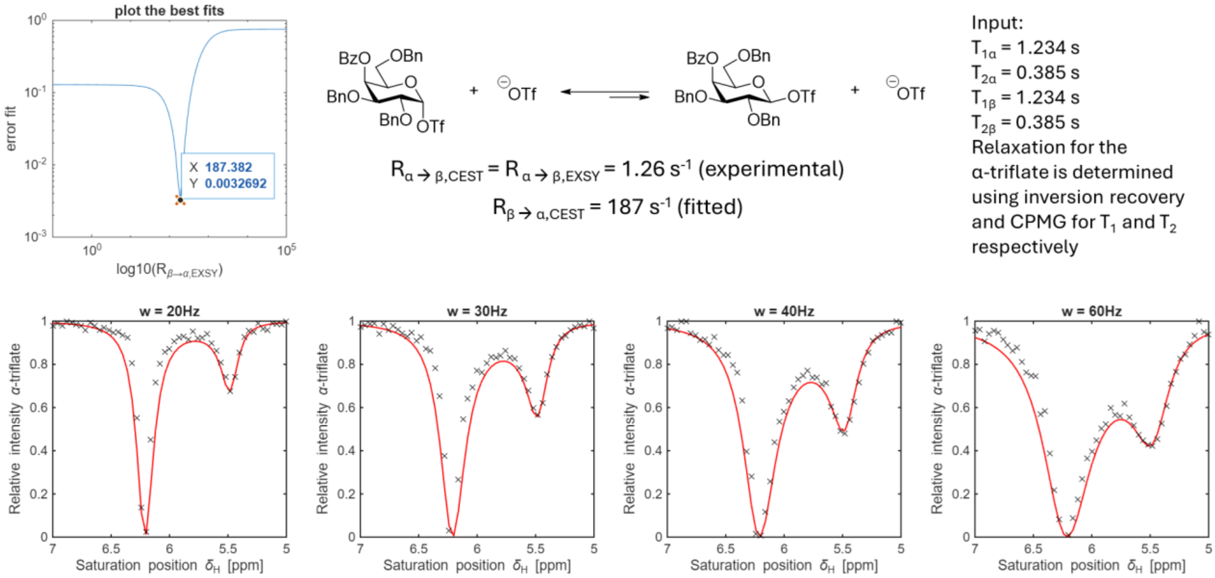

Figure S24: Single parameter fit outcome where the $R_{\alpha\rightarrow\beta,\ CEST}$ was fixed to the value of triflate dissociation found using ^{19}F EXSY ($R_{\alpha\rightarrow OTf,\ EXSY}$) and T_1 and T_2 for the axial α-triflate was measured using inversion recovery and CPMG respectively. The relaxation rates for the equatorial β-triflate were assumed to be the same as the axial α-triflate. The $R_{\beta\rightarrow\alpha,\ CEST}$ was determined by the Bloch-McConnel fitting to the CEST data measured at saturation field strengths of 20, 30, 40, and 60 Hz. The error at each simulation for $R_{\beta\rightarrow\alpha,\ CEST}$ was plotted against the rate which resulted in the best fits at the given $R_{\beta\rightarrow\alpha,\ CEST}$. This was in good agreement with the multi-parameter fit where both $R_{\beta\rightarrow\alpha,\ CEST}$ and $R_{\alpha\rightarrow\beta,\ CEST}$ were determined.

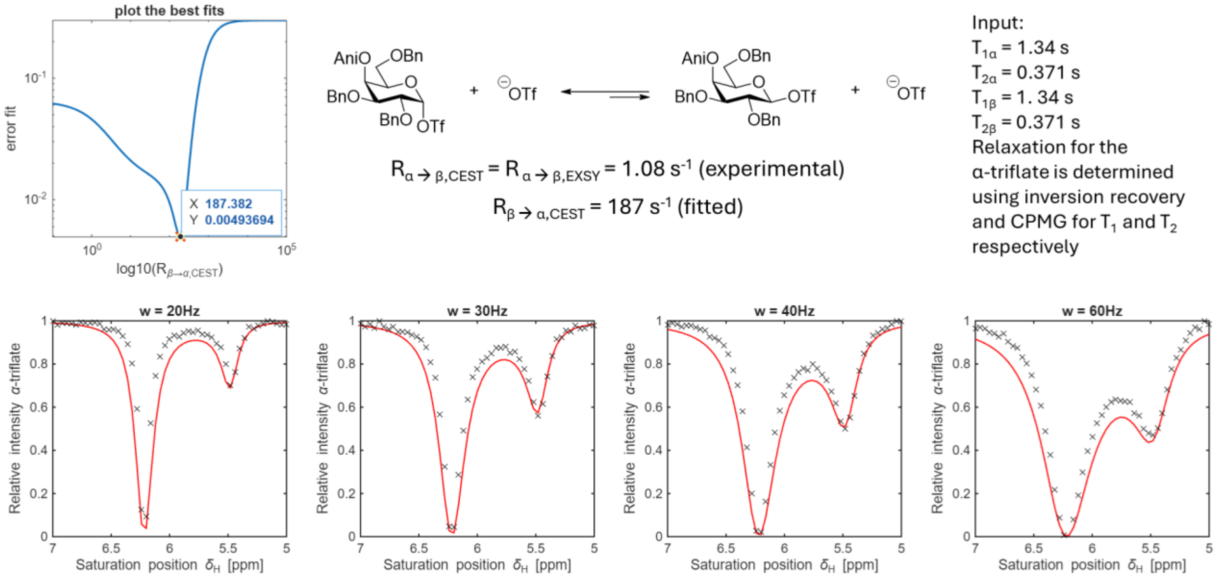

Figure S25: Single parameter fit outcome where the $R_{\alpha\rightarrow\beta,\ CEST}$ was fixed to the value of triflate dissociation found using ^{19}F EXSY ($R_{\alpha\rightarrow OTf,\ EXSY}$) and T_1 and T_2 for the axial α-triflate was measured using inversion recovery and CPMG respectively. The relaxation rates for the equatorial β-triflate were assumed to be the same as the axial α-triflate. The $R_{\beta\rightarrow\alpha,\ CEST}$ was determined by the Bloch-McConnel fitting to the CEST data measured at saturation field strengths of 20, 30, 40, and 60 Hz. The error at each simulation for $R_{\beta\rightarrow\alpha,\ CEST}$ was plotted against the rate which resulted in the best fits at the given $R_{\beta\rightarrow\alpha,\ CEST}$. This was in good agreement with the multi-parameter fit where both $R_{\beta\rightarrow\alpha,\ CEST}$ and $R_{\alpha\rightarrow\beta,\ CEST}$ were determined.

T_1 and T_2 test for fitting

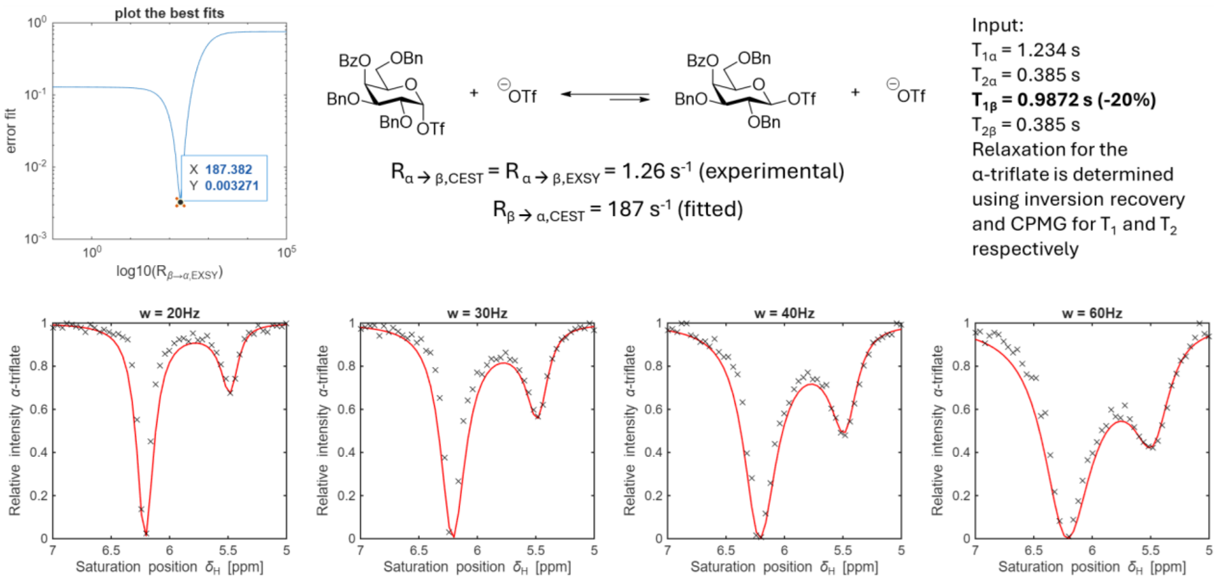

Figure S26: Fitting results for testing the influence of the relaxation input values for the equatorial β-triflate. The T_1 relaxation for the axial α-triflate was set to 1.234 seconds as measured with inversion recovery NMR and the T_2 of the axial α-triflate its H-1 proton was fixed to 0.385 seconds which was measured with CPMG NMR. The T_2 of the equatorial β-triflate was fixed to the experimental value for the axial α-triflate whilst the T_1 was set 20% lower to the measured value for the axial α-triflate demonstrating that a 20% change is not affecting the fitting result much.

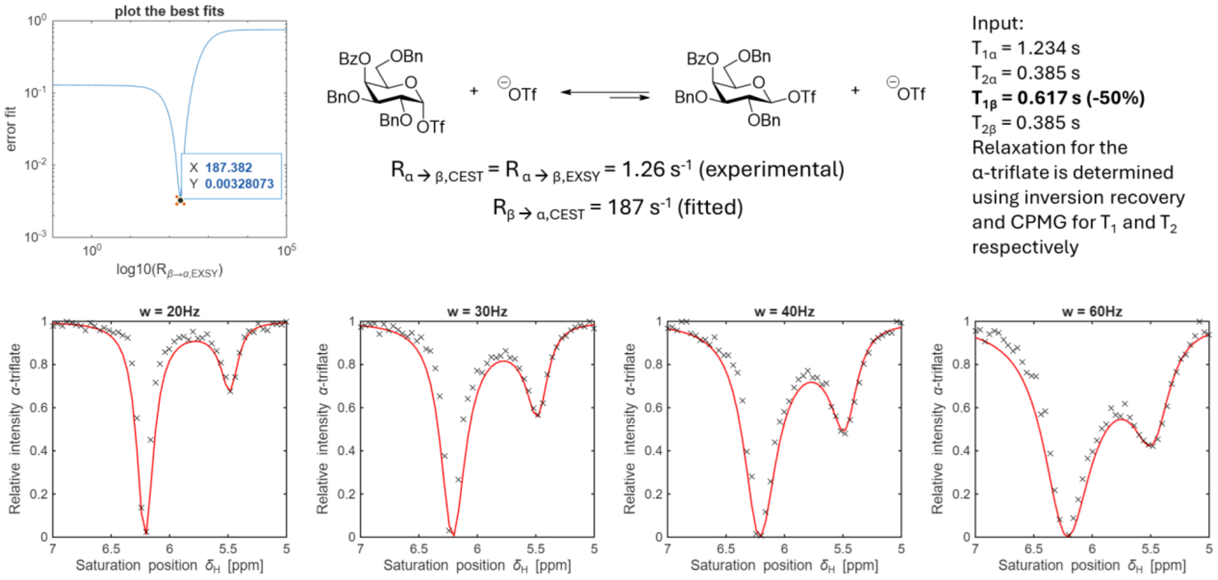

Figure S27: Fitting results for testing the influence of the relaxation input values for the equatorial β-triflate. The T_1 relaxation for the axial α-triflate was set to 1.234 seconds as measured with inversion recovery NMR and the T_2 of the axial α-triflate its H-1 proton was fixed to 0.385 seconds which was measured with CPMG NMR. The T_2 of the equatorial β-triflate was fixed to the experimental value for the axial α-triflate whilst the T_1 was set 50% lower to the measured value for the axial α-triflate demonstrating that a 50% change is not affecting the fitting result much.

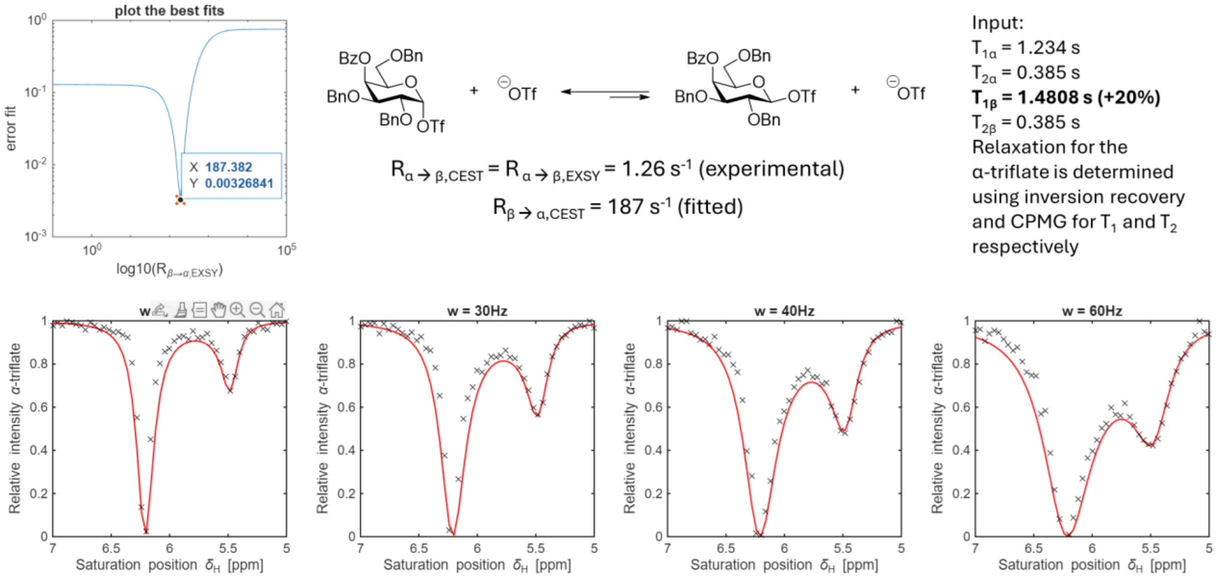

Figure S28: Fitting results for testing the influence of the relaxation input values for the equatorial β-triflate. The T_1 relaxation for the axial α-triflate was set to 1.234 seconds as measured with inversion recovery NMR and the T_2 of the axial α-triflate its H-1 proton was fixed to 0.385 seconds which was measured with CPMG NMR. The T_2 of the equatorial β-triflate was fixed to the experimental value for the axial α-triflate whilst the T_1 was set 20% higher to the measured value for the axial α-triflate demonstrating that a 20% change is not affecting the fitting result much.

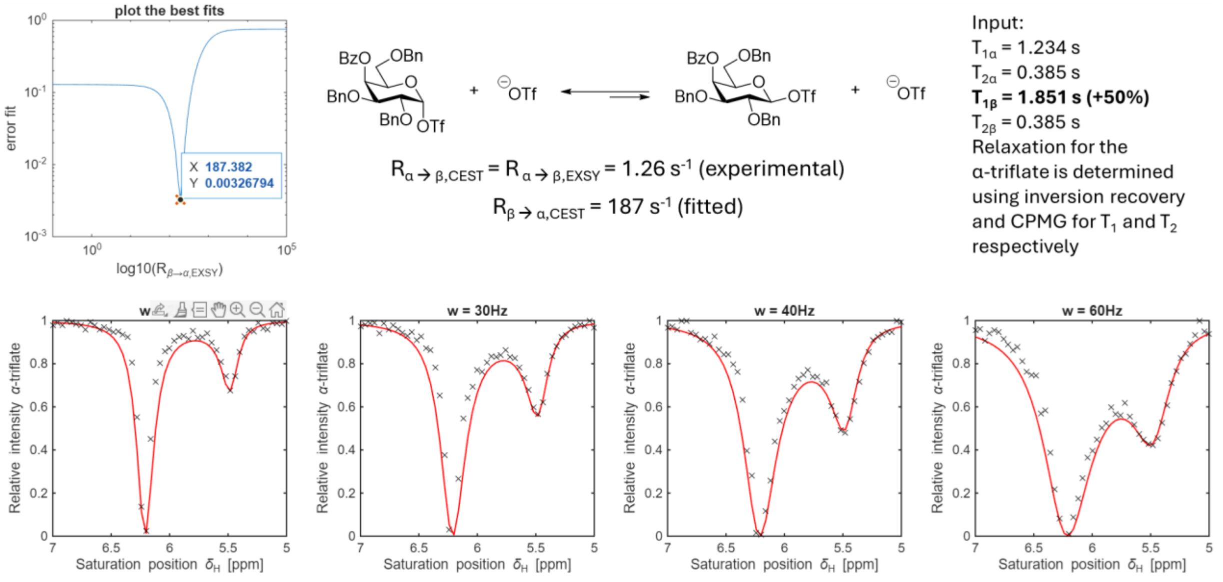

Figure S29: Fitting results for testing the influence of the relaxation input values for the equatorial β-triflate. The T_1 relaxation for the axial α-triflate was set to 1.234 seconds as measured with inversion recovery NMR and the T_2 of the axial α-triflate its H-1 proton was fixed to 0.385 seconds which was measured with CPMG NMR. The T_2 of the equatorial β-triflate was fixed to the experimental value for the axial α-triflate whilst the T_1 was set 50% higher to the measured value for the axial α-triflate demonstrating that a 50% change is not affecting the fitting result much.

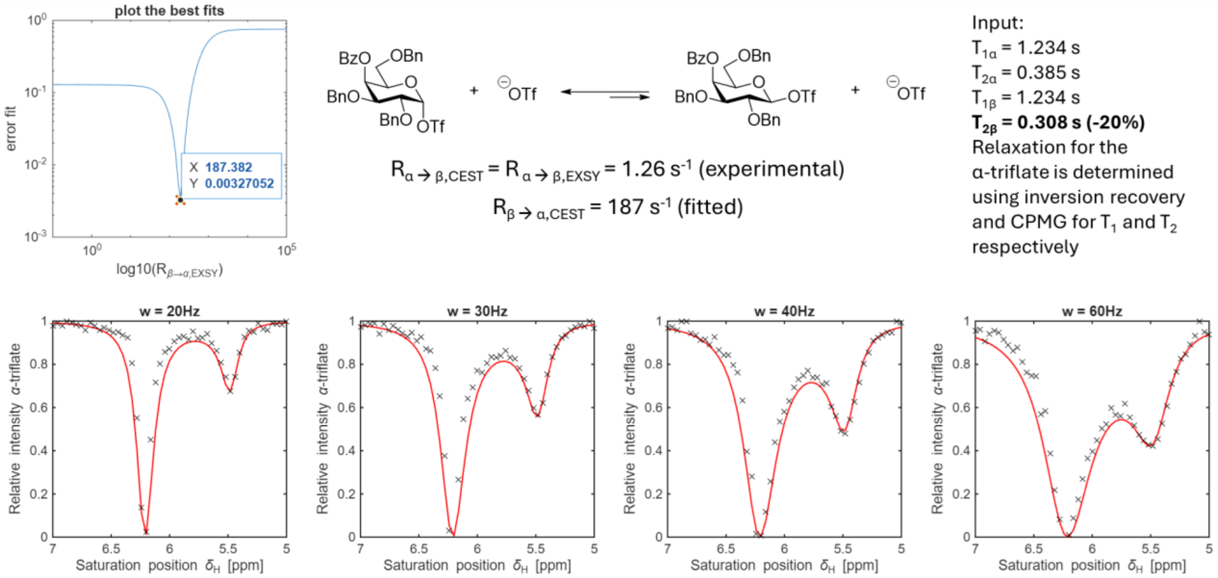

Figure S30: Fitting results for testing the influence of the relaxation input values for the equatorial β-triflate. The T_1 relaxation for the axial α-triflate was set to 1.234 seconds as measured with inversion recovery NMR and the T_2 of the axial α-triflate its H-1 proton was fixed to 0.385 seconds which was measured with CPMG NMR. The T_1 of the equatorial β-triflate was fixed to the experimental value for the axial α-triflate whilst the T_2 was set 20% lower to the measured value for the axial α-triflate demonstrating that a 20% change is not affecting the fitting result much.

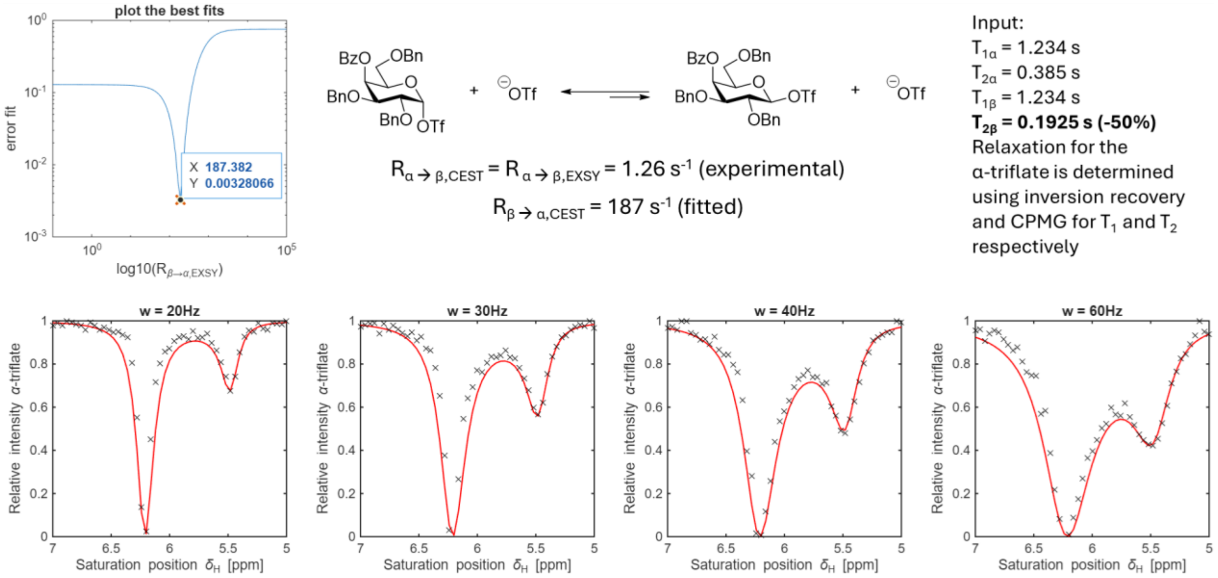

Figure S31: Fitting results for testing the influence of the relaxation input values for the equatorial β-triflate. The T_1 relaxation for the axial α-triflate was set to 1.234 seconds as measured with inversion recovery NMR and the T_2 of the axial α-triflate its H-1 proton was fixed to 0.385 seconds which was measured with CPMG NMR. The T_1 of the equatorial β-triflate was fixed to the experimental value for the axial α-triflate whilst the T_2 was set 50% lower to the measured value for the axial α-triflate demonstrating that a 50% change is not affecting the fitting result much.

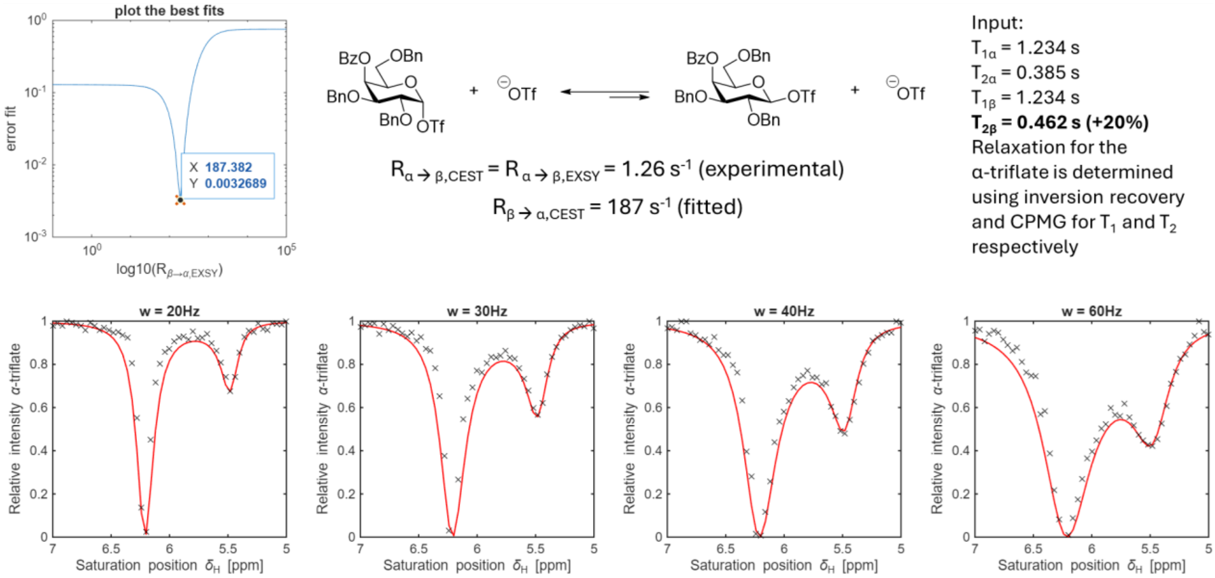

Figure S32: Fitting results for testing the influence of the relaxation input values for the equatorial β-triflate. The T_1 relaxation for the axial α-triflate was set to 1.234 seconds as measured with inversion recovery NMR and the T_2 of the axial α-triflate its H-1 proton was fixed to 0.385 seconds which was measured with CPMG NMR. The T_1 of the equatorial β-triflate was fixed to the experimental value for the axial α-triflate whilst the T_2 was set 20% higher to the measured value for the axial α-triflate demonstrating that a 20% change is not affecting the fitting result much.

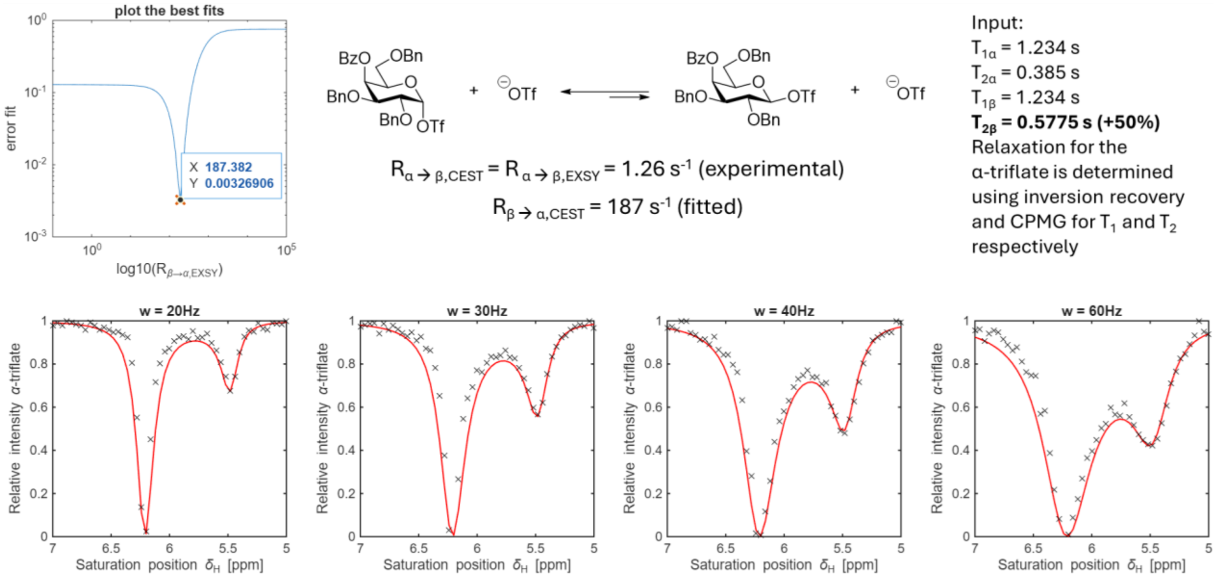

Figure S33: Fitting results for testing the influence of the relaxation input values for the equatorial β-triflate. The T_1 relaxation for the axial α-triflate was set to 1.234 seconds as measured with inversion recovery NMR and the T_2 of the axial α-triflate its H-1 proton was fixed to 0.385 seconds which was measured with CPMG NMR. The T_1 of the equatorial β-triflate was fixed to the experimental value for the axial α-triflate whilst the T_2 was set 50% lower to the measured value for the axial α-triflate demonstrating that a 50% change is not affecting the fitting result much.

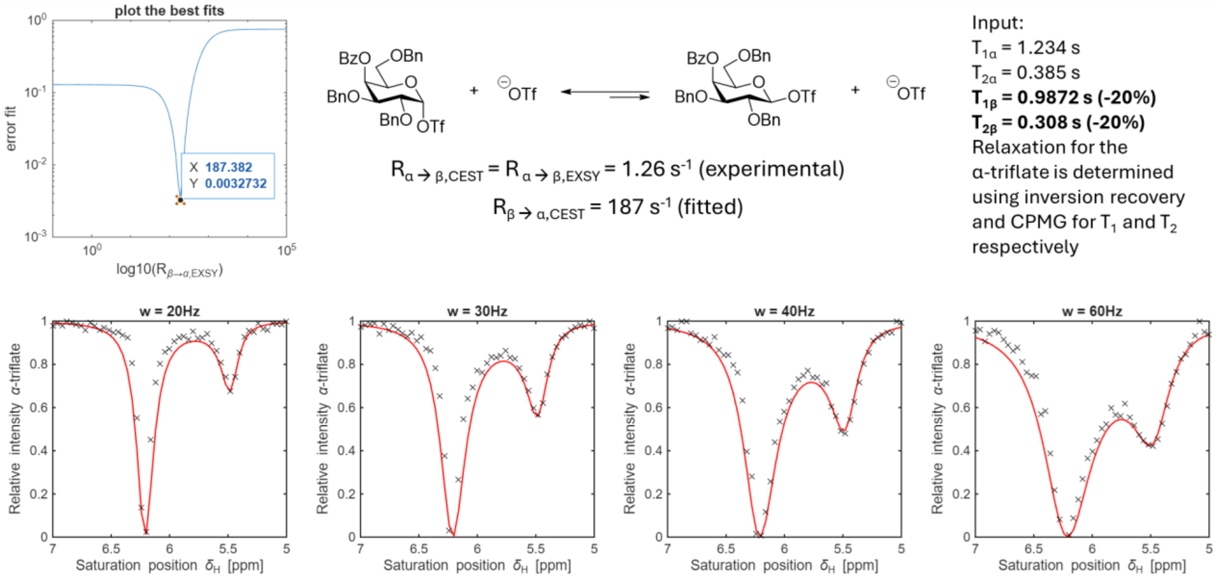

Figure S34: Fitting results for testing the influence of the relaxation input values for the equatorial β-triflate. The T_1 relaxation for the axial α-triflate was set to 1.234 seconds as measured with inversion recovery NMR and the T_2 of the axial α-triflate its H-1 proton was fixed to 0.385 seconds which was measured with CPMG NMR. The T_1 and T_2 of the equatorial β-triflate was set 20% lower to the measured value for the axial α-triflate demonstrating that a 20% change is not affecting the fitting result much.

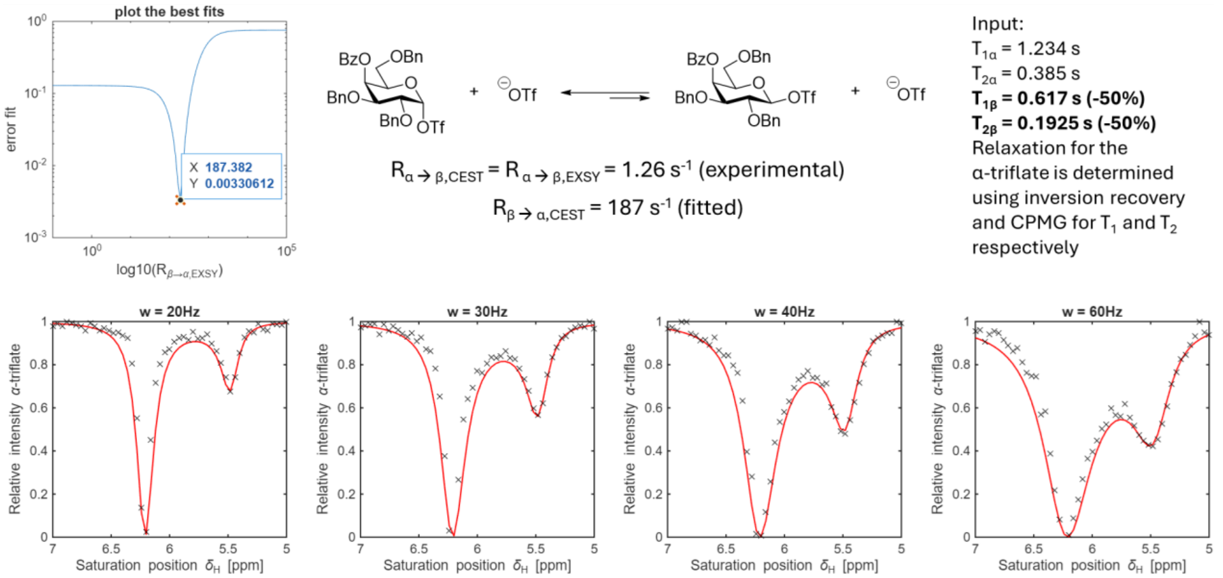

Figure S35: Fitting results for testing the influence of the relaxation input values for the equatorial β-triflate. The T_1 relaxation for the axial α-triflate was set to 1.234 seconds as measured with inversion recovery NMR and the T_2 of the axial α-triflate its H-1 proton was fixed to 0.385 seconds which was measured with CPMG NMR. The T_1 and T_2 of the equatorial β-triflate was set 50% lower to the measured value for the axial α-triflate demonstrating that a 50% change is not affecting the fitting result much.

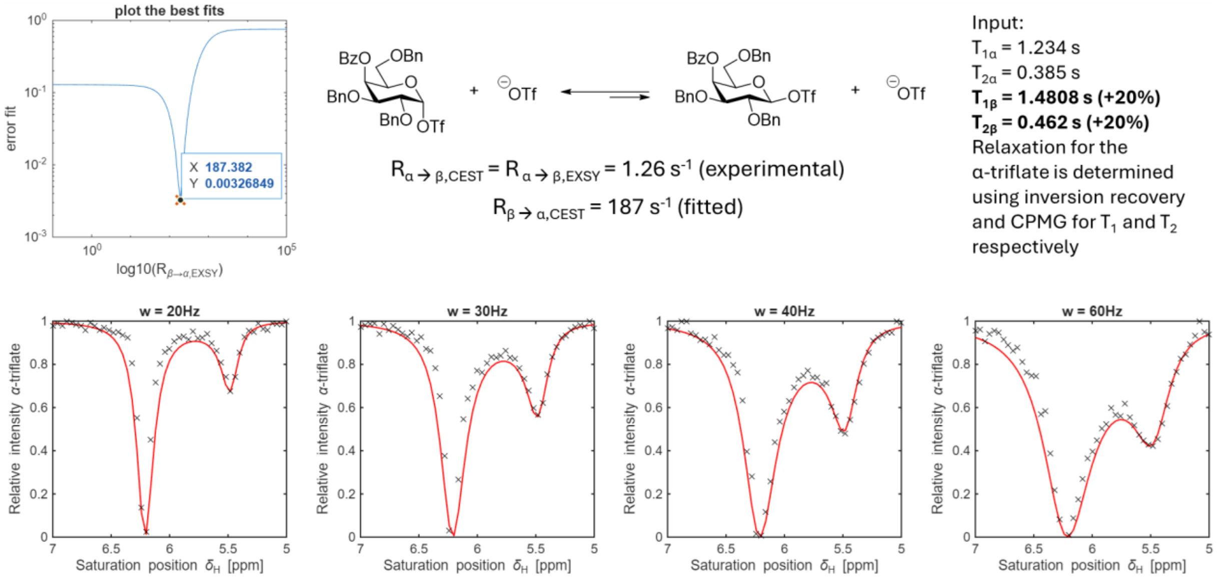

Figure S36: Fitting results for testing the influence of the relaxation input values for the equatorial β-triflate. The T_1 relaxation for the axial α-triflate was set to 1.234 seconds as measured with inversion recovery NMR and the T_2 of the axial α-triflate its H-1 proton was fixed to 0.385 seconds which was measured with CPMG NMR. The T_1 and T_2 of the equatorial β-triflate was set 20% higher to the measured value for the axial α-triflate demonstrating that a 20% change is not affecting the fitting result much.

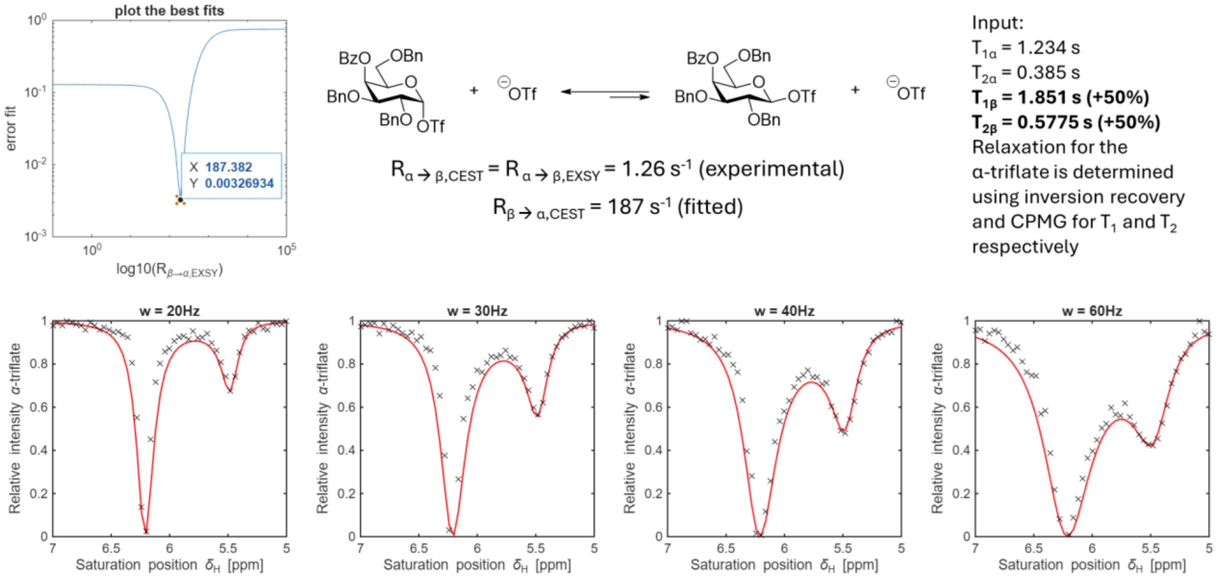

Figure S37: Fitting results for testing the influence of the relaxation input values for the equatorial β-triflate. The T_1 relaxation for the axial α-triflate was set to 1.234 seconds as measured with inversion recovery NMR and the T_2 of the axial α-triflate its H-1 proton was fixed to 0.385 seconds which was measured with CPMG NMR. The T_1 and T_2 of the equatorial β-triflate was set 50% higher to the measured value for the axial α-triflate demonstrating that a 50% change is not affecting the fitting result much.

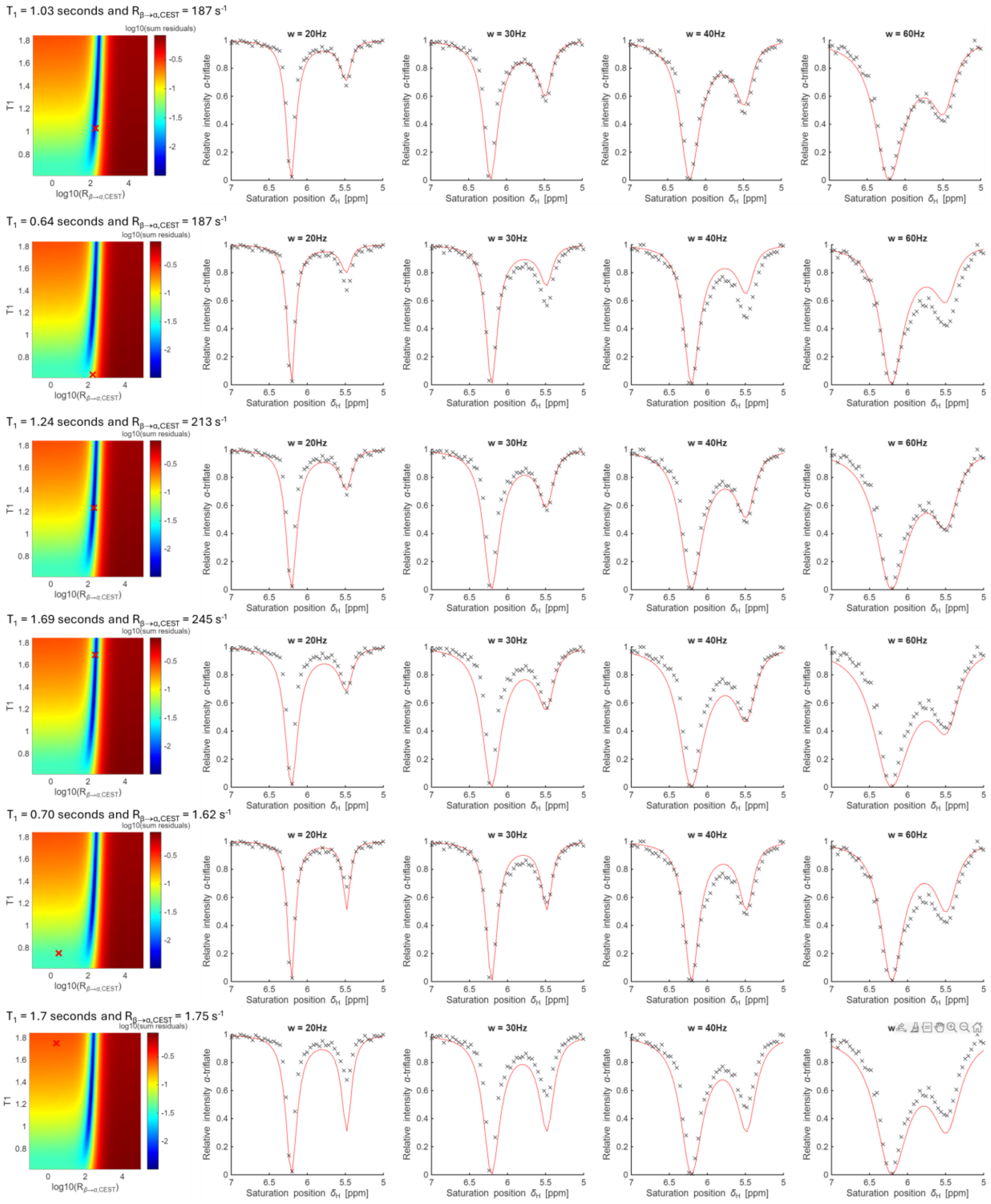

Figure S38: Multiparameter fitting changing both the T_1 of the equatorial β-triflate and rate of equatorial β-triflate to axial α-triflate interconversion rate ($R_{\beta\rightarrow\alpha,\ CEST}$) to study the range of possible combinations of the two for reasonable fitting results. These results highlight that the most reasonable fits are achieved with a $R_{\beta\rightarrow\alpha,\ CEST} \approx 200\ s^{-1}$ with T_1-values spanning from about 0.8 to 1.8 seconds highlighting that assuming a T_1 for the equatorial β-triflate to be about the T_1 for the axial α-triflate is reasonable. Even if there is a slight deviation between the anomeric protons in either the axial α-triflate and equatorial β-triflate then this likely falls within the boundaries (0.8 to 1.8 seconds) due to the structural similarities between the two species.

Supporting transition state calculations

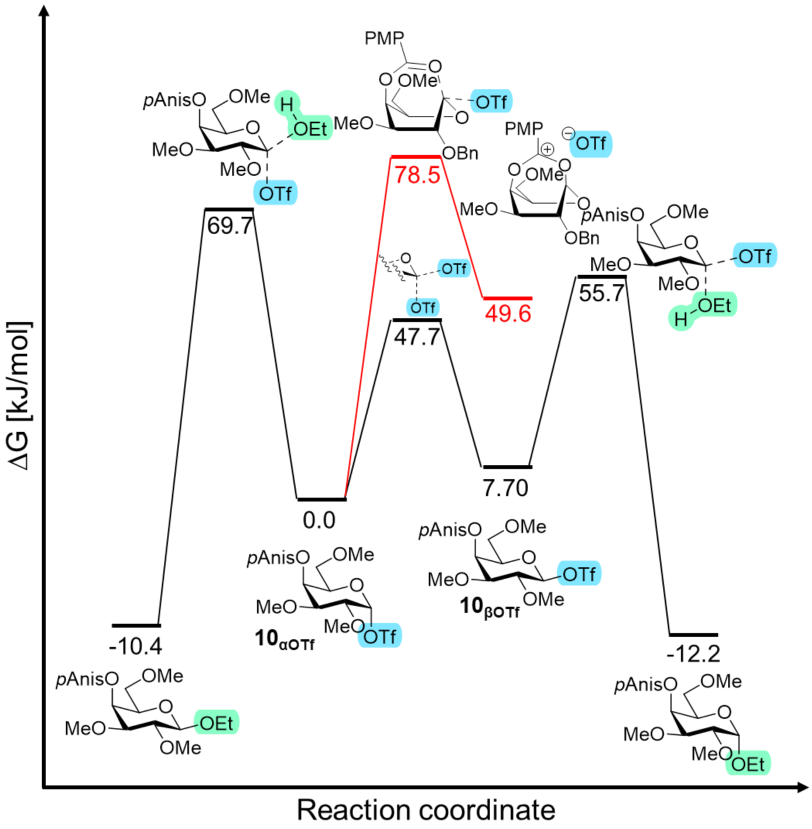

Figure S39: DFT-computed Gibbs free energy surface in DCM (ΔG_{DCM}, kJ/mol) for the methylated analogues $6\alpha_{OTf}$ and $6\beta_{OTf}$, including their interconversion barrier. Transition state energies for their reaction with a primary acceptor (ethanol) were also calculated. Additionally, the transition state from $6\alpha_{OTf}$ towards formation of the 1,4 dioxepanium ion and its relative energy were determined (red line). The energy of $10_{\alpha OTf}$ was set as the reference point (0 kJ/mol).

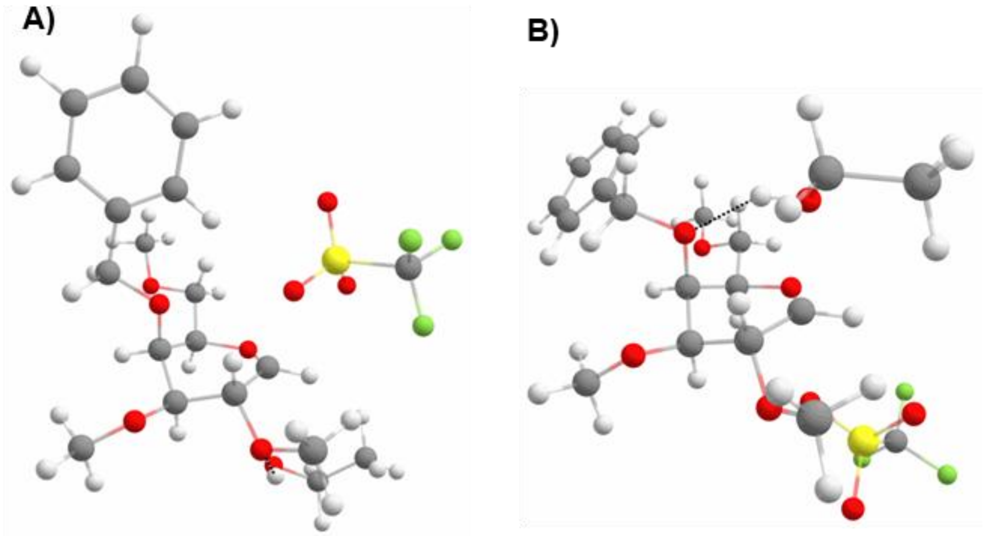

Figure S40: Product forming transitions states of the methylated analogues of perbenzylated donor **4** A) Structure of TS-$8_{\beta OTf}$,OEt. A hydrogen bond is observed between $H_{ethanol}$ nucleophile and the O-2 methyl group (black dashed line, 1.96 Å). Other relevant distances: C_1-$O_{ethanol}$ 2.88 Å and C_1-O_{OTf} 2.19 Å. B) Structure of TS-$8_{\alpha OTf}$,OEt. A hydrogen bond is observed between $H_{ethanol}$ nucleophile and the O-4 of the benzyl group (black dashed line, 1.97 Å). Other relevant distances: C_1-$O_{ethanol}$ bond length of 2.45 Å and C_1-O_{OTf} 2.59 Å.

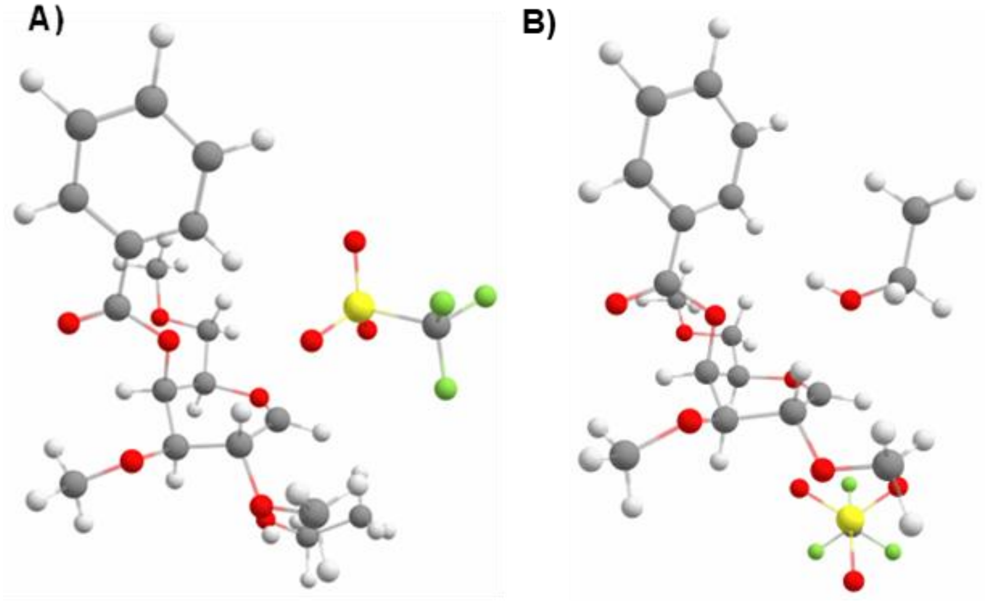

Figure S41: Product forming transitions states of the methylated analogues of C-4 benzoylated donor **5**. A) Structure of TS-$9_{\beta OTf}$,OEt. No hydrogen bond is observed between $H_{ethanol}$ nucleophile and the O-2 methyl group (2.00 Å). Other relevant distances: C_1-$O_{ethanol}$ 2.82 Å and C_1-O_{OTf} 2.20 Å; B) Structure of TS-$9_{\alpha OTf}$,OEt. No hydrogen bond is observed between $H_{ethanol}$ nucleophile and the O-4 of the benzyl group (2.47 Å). Other relevant distances: C_1-$O_{ethanol}$ of 2.40 Å and C_1-O_{OTf} 2.66 Å.

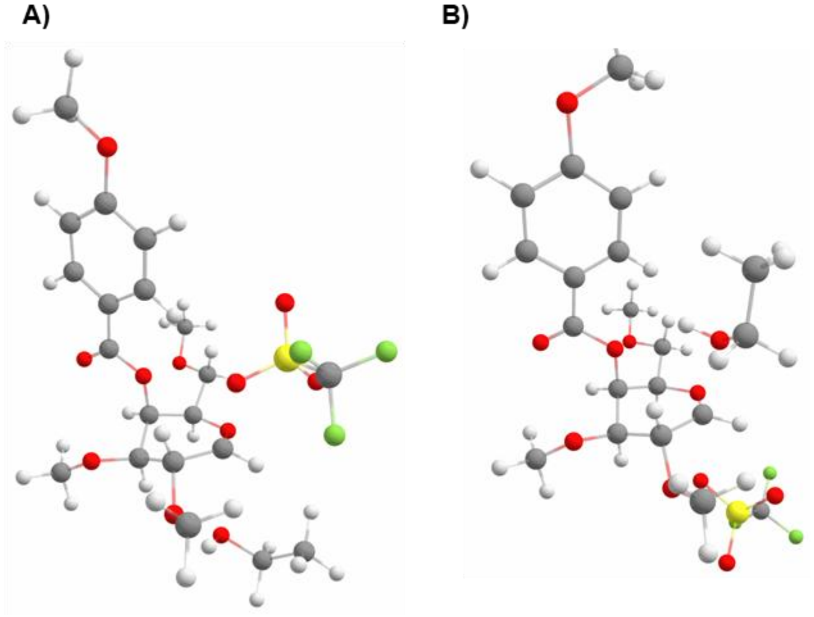

Figure S42: Product forming transitions states of the methylated analogues of C-4 p-anisoylated donor **6**. A) Structure of TS-10βOTf,OEt. No hydrogen bond is observed between Hethanol nucleophile and the O-2 methyl group (2.00 Å). Other relevant distances: C¬1-Oethanol 2.83 Å and C¬1-OOTf 2.19 Å. B) Structure of TS-10αOTf,OEt. No hydrogen bond is observed between Hethanol nucleophile and the O-4 of the benzyl group (2.44 Å). Other relevant distances: C¬1-Oethanol 2.40 Å and C¬1-OOTf 2.66 Å.

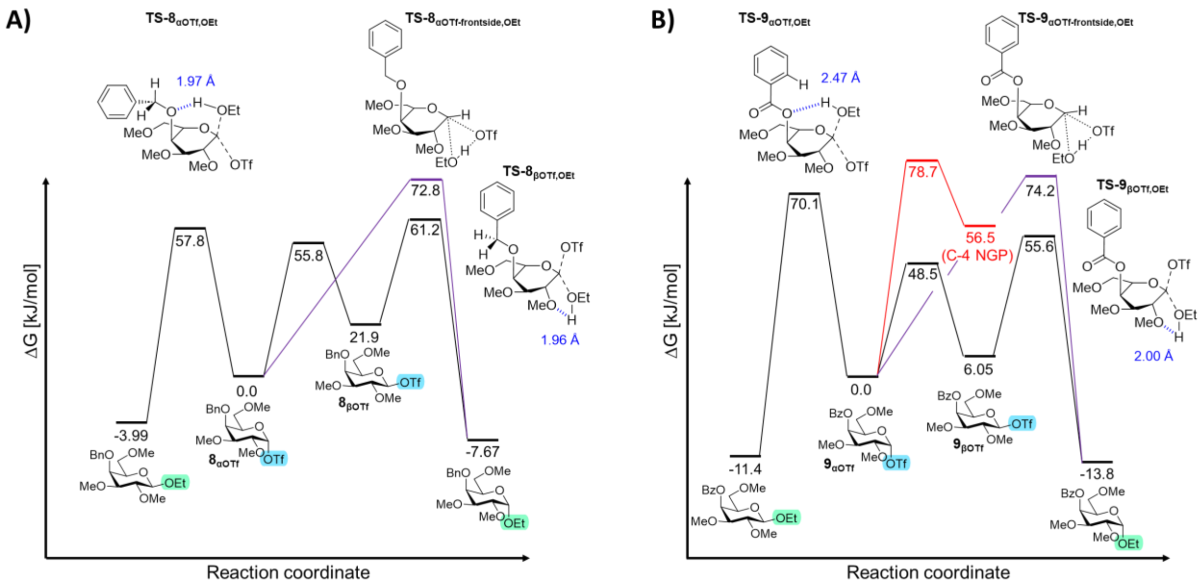

Figure S43: Reaction coordinates comparing a Curtin Hammett mechanism via covalent triflates with a front-side axial α-triflate displacement (A). Reaction coordinates including product forming pathways including axial α-triflates and equatorial β-triflates, C-4 benzoyl NGP, and a front-side S_N2 reaction on the axial α-triflate (B). Both reaction coordinates suggest a Curtin-Hammet mechanism via covalent triflates to yield both axial α- and equatorial β-products. As always the position of the transition state is not on scale and therefore does not resemble the degree of early or late transition states, this is particularly emphasized with the addition of the front-side mechanism which could not be drawn in the middle.

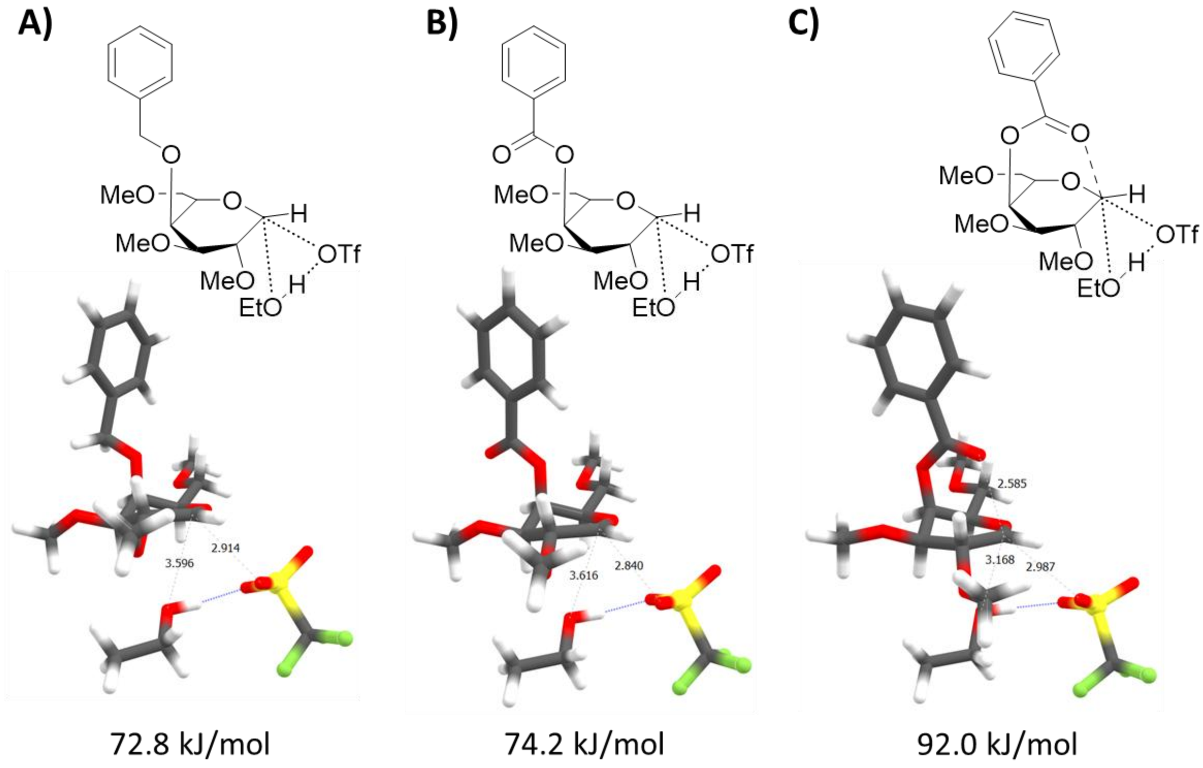

Figure S44: Transition states of the axial α-triflate substitution with ethanol with bond lengths indicated for a front side attack for the 4-*O*-benzyl galactosyl donor (A) and 4-*O*-benzoyl galactosyl donor with and without coordination of the C-4 benzoyl (B and C respectively).

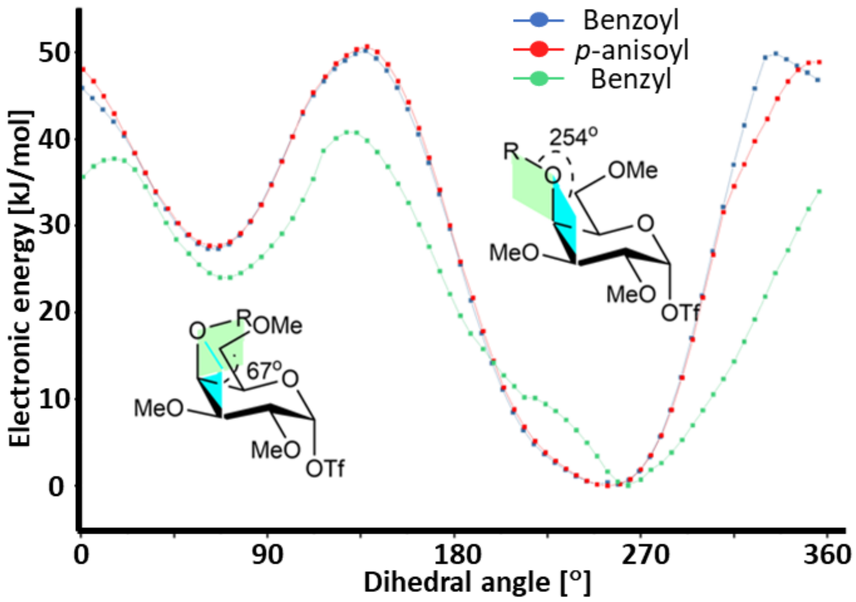

Figure S45: Potential energy surface scan rotating the substituent on position 4.

References

1. de Kleijne, F. F.; Ter Braak, F.; Piperoudis, D.; Moons, P. H.; Moons, S. J.; Elferink, H.; White, P. B.; Boltje, T. J., Detection and Characterization of Rapidly Equilibrating Glycosylation Reaction Intermediates Using Exchange NMR. *Journal of the American Chemical Society* **2023,** *145* (48), 26190-26201.
2. Lokesh, N.; Seegerer, A.; Hioe, J.; Gschwind, R. M., Chemical exchange saturation transfer in chemical reactions: a mechanistic tool for NMR detection and characterization of transient intermediates. *Journal of the American Chemical Society* **2018,** *140* (5), 1855-1862.
3. McMahon, M. T.; Gilad, A. A.; Zhou, J.; Sun, P. Z.; Bulte, J. W.; Van Zijl, P. C., Quantifying exchange rates in chemical exchange saturation transfer agents using the saturation time and saturation power dependencies of the magnetization transfer effect on the magnetic resonance imaging signal (QUEST and QUESP): pH calibration for poly-L-lysine and a starburst dendrimer. *Magnetic Resonance in Medicine: An Official Journal of the International Society for Magnetic Resonance in Medicine* **2006,** *55* (4), 836-847.
4. Zhou, J.; van Zijl, P. C., Chemical exchange saturation transfer imaging and spectroscopy. *Progress in Nuclear Magnetic Resonance Spectroscopy* **2006,** *48* (2-3), 109-136.
5. Woessner, D. E.; Zhang, S.; Merritt, M. E.; Sherry, A. D., Numerical solution of the Bloch equations provides insights into the optimum design of PARACEST agents for MRI. *Magnetic Resonance in Medicine: An Official Journal of the International Society for Magnetic Resonance in Medicine* **2005,** *53* (4), 790-799.
6. Zaiss, M.; Angelovski, G.; Demetriou, E.; McMahon, M. T.; Golay, X.; Scheffler, K., QUESP and QUEST revisited–fast and accurate quantitative CEST experiments. *Magnetic resonance in medicine* **2018,** *79* (3), 1708-1721.
7. Behera, A.; Rai, D.; Kushwaha, D.; Kulkarni, S. S., Total synthesis of trisaccharide repeating unit of O-specific polysaccharide of Pseudomonas fluorescens BIM B-582. *Organic Letters* **2018,** *20* (18), 5956-5959.
8. Dharuman, S.; Amarasekara, H.; Crich, D., Interplay of protecting groups and side chain conformation in glycopyranosides. Modulation of the influence of remote substituents on glycosylation? *The Journal of Organic Chemistry* **2018,** *83* (17), 10334-10351.
9. Hansen, T.; Elferink, H.; van Hengst, J. M.; Houthuijs, K. J.; Remmerswaal, W. A.; Kromm, A.; Berden, G.; van der Vorm, S.; Rijs, A. M.; Overkleeft, H. S., Characterization of glycosyl dioxolenium ions and their role in glycosylation reactions. *Nature Communications* **2020,** *11* (1), 2664.
10. Pracht, P.; Bohle, F.; Grimme, S., Automated exploration of the low-energy chemical space with fast quantum chemical methods. *Physical Chemistry Chemical Physics* **2020,** *22* (14), 7169-7192.
11. Frisch, M.; Clemente, F., MJ Frisch, GW Trucks, HB Schlegel, GE Scuseria, MA Robb, JR Cheeseman, G. *Scalmani, V. Barone, B. Mennucci, GA Petersson, H. Nakatsuji, M. Caricato, X. Li, HP Hratchian, AF Izmaylov, J. Bloino and G. Zhe, Gaussian 9*.
12. Neese, F., Software update: The ORCA program system—Version 5.0. WIREs Computational Molecular Science 12: e1606. 2022.
13. Bock, K.; Pedersen, C., A study of 13 CH coupling constants in hexopyranoses. *Journal of the Chemical Society, Perkin Transactions 2* **1974,** (3), 293-297.
14. Upadhyaya, K.; Subedi, Y. P.; Crich, D., Direct experimental characterization of a bridged bicyclic glycosyl dioxacarbenium ion by 1H and 13C NMR spectroscopy: Importance of conformation on participation by distal esters. *Angewandte Chemie International Edition* **2021,** *60* (48), 25397-25403.
15. de Kleijne, F. F.; Elferink, H.; Moons, S. J.; White, P. B.; Boltje, T. J., Characterization of mannosyl dioxanium ions in solution using chemical exchange saturation transfer NMR spectroscopy. *Angewandte Chemie International Edition* **2022,** *61* (6), e202109874.
16. Frihed, T. G.; Walvoort, M. T.; Codee, J. D.; van der Marel, G. A.; Bols, M.; Pedersen, C. M., Influence of O6 in mannosylations using benzylidene protected donors: stereoelectronic or conformational effects? *The Journal of Organic Chemistry* **2013,** *78* (6), 2191-2205.

17. Ande, C.; Crich, D., Stereodirecting Effect of Esters at the 4-Position of Galacto-and Glucopyranosyl Donors: Effect of 4-C-Methylation on Side-Chain Conformation and Donor Reactivity, and Influence of Concentration and Stoichiometry on Distal Group Participation. *The Journal of Organic Chemistry* **2023,** *88* (19), 13883-13893.

Raw data
EXSY at different temperatures

Selective 1D ^{19}F EXSY data at -80 ^{o}C

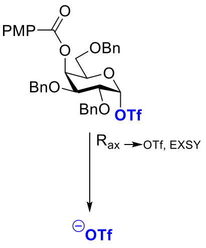

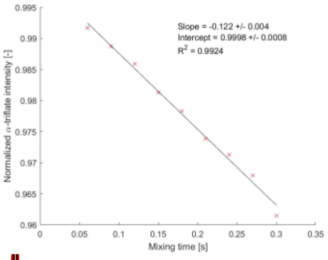

Spectrum number	Mixing Time [s]	α-triflate (-75.86 ppm) resonance intensity	$^{-}$OTf (-78.86 ppm) resonance intensity
1	0.06	4.87254	0.040678
2	0.09	4.42876	0.050603
3	0.12	4.02463	0.057541
4	0.15	3.65603	0.069789
5	0.18	3.34165	0.074155
6	0.21	3.04504	0.081794
7	0.24	2.75078	0.081452
8	0.27	2.49631	0.082655
9	0.3	2.29488	0.091918

Temperature = -80 ^{o}C

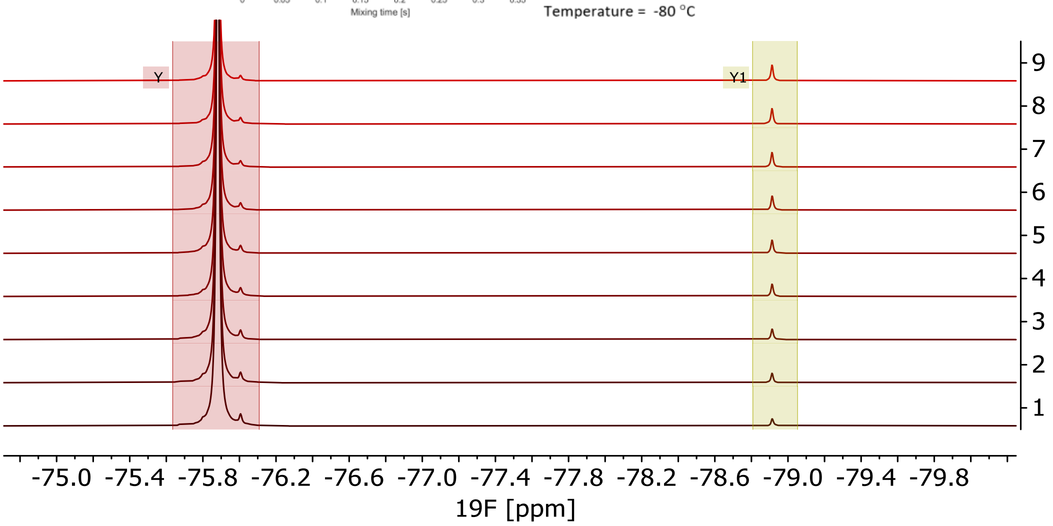

Figure S46: Raw ^{19}F EXSY data for the α-triflate dissociation in the corresponding α-triflate.

Selective 1D ^{19}F EXSY data at -70 ^{o}C

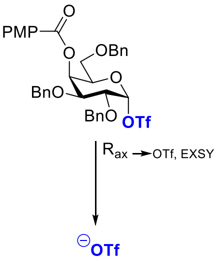

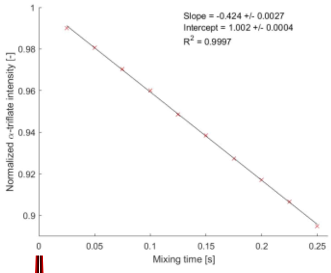

Spectrum number	Mixing Time [s]	α-triflate (-75.86 ppm) resonance intensity	$^{-}$OTf (-78.86 ppm) resonance intensity
1	0.025	6.64178	0.066229
2	0.05	6.22251	0.122401
3	0.075	5.67653	0.17382
4	0.1	5.1412	0.213341
5	0.125	4.67785	0.253652
6	0.15	4.29924	0.281811
7	0.175	3.96402	0.310534
8	0.2	3.62708	0.327625
9	0.225	3.36197	0.345987
10	0.25	3.08545	0.362566

Temperature = -70°C

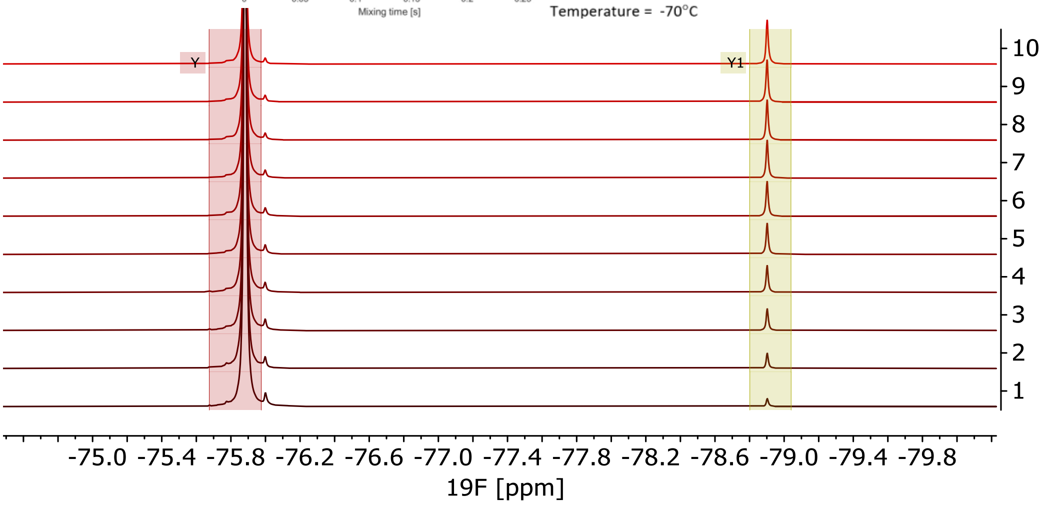

Figure S47: Raw ^{19}F EXSY data for the α-triflate dissociation in the corresponding α-triflate.

Selective 1D ^{19}F EXSY data at -60 ^{o}C

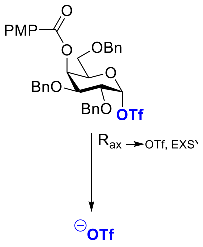

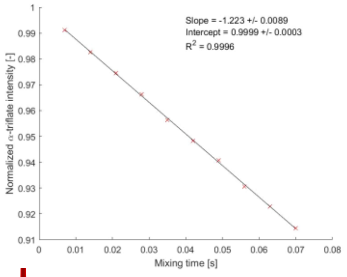

Spectrum number	Mixing Time [s]	α-triflate (-75.86 ppm) resonance intensity	$^{-}$OTf (-78.86 ppm) resonance intensity
1	0.007	6.38582	0.056691
2	0.014	6.03625	0.106801
3	0.021	5.88881	0.153653
4	0.028	5.86285	0.204932
5	0.035	5.51677	0.252313
6	0.042	5.42239	0.296153
7	0.049	5.37558	0.338429
8	0.056	5.07826	0.378605
9	0.063	4.99811	0.417512
10	0.07	4.91558	0.459805

Temperature = -60 ^{o}C

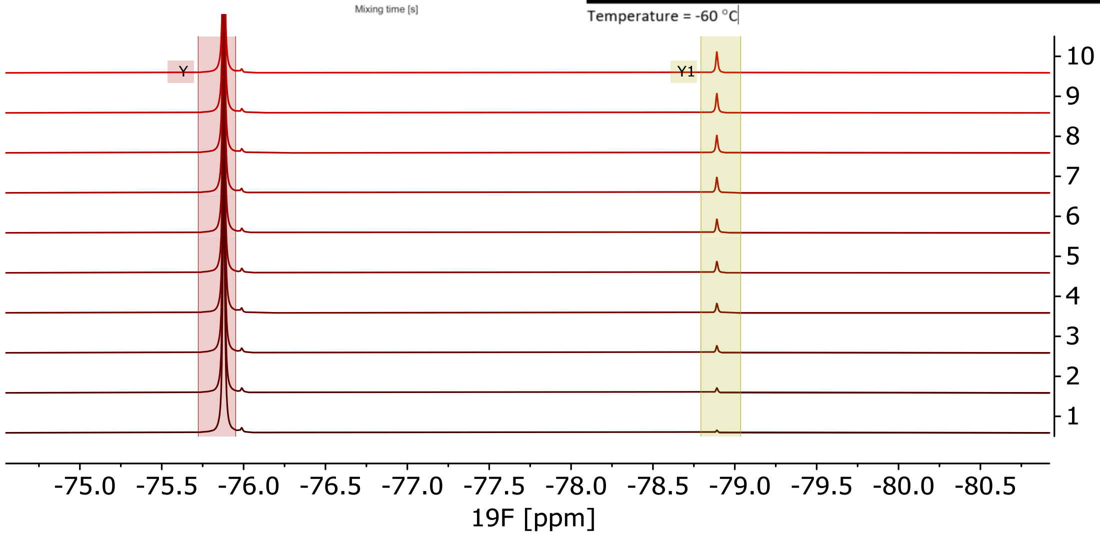

Figure S48: Raw ^{19}F EXSY data for the α-triflate dissociation in the corresponding α-triflate.

Selective 1D ^{19}F EXSY data at -50 ^{o}C

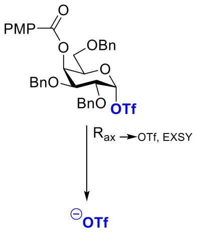

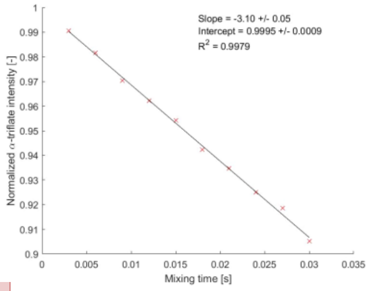

Spectrum number	Mixing Time [s]	α-triflate (-75.86 ppm) resonance intensity	$^{-}$OTf (-78.86 ppm) resonance intensity
1	0.003	6.53084	0.061613
2	0.006	6.47877	0.121891
3	0.009	6.07212	0.185474
4	0.012	6.09645	0.23978
5	0.015	6.1481	0.295393
6	0.018	5.77832	0.353377
7	0.021	5.70029	0.398577
8	0.024	5.69693	0.461755
9	0.027	5.61333	0.497752
10	0.03	5.28986	0.554873

Temperature = -50 ^{o}C

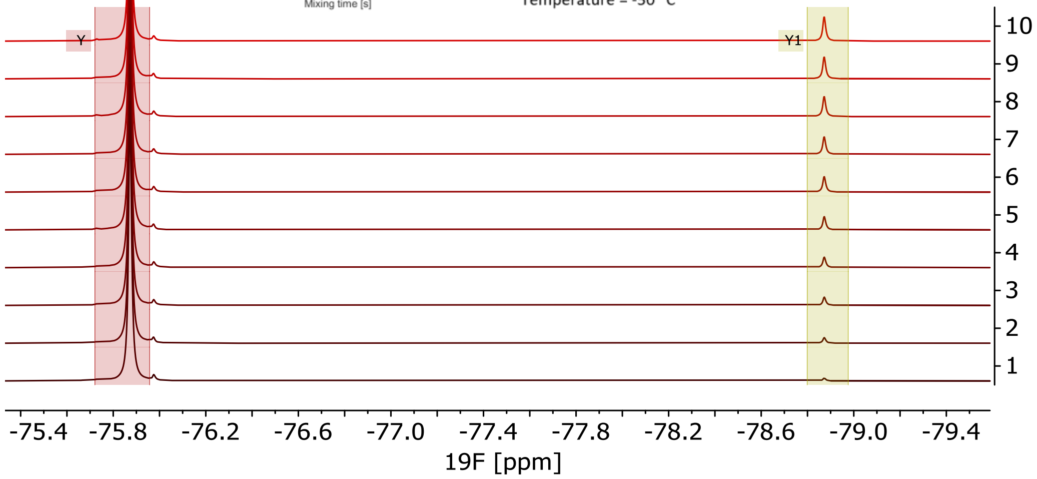

Figure S49: Raw ^{19}F EXSY data for the α-triflate dissociation in the corresponding α-triflate.

Selective 1D ^{19}F EXSY data at -40 ^{o}C

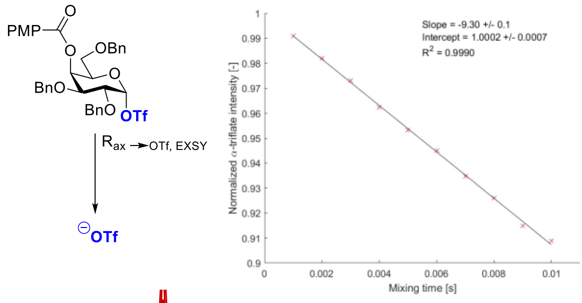

Spectrum number	Mixing Time [s]	α-triflate (-75.86 ppm) resonance intensity	¯OTf (-78.86 ppm) resonance intensity
1	0.001	4.15088	0.038157
2	0.002	4.22296	0.077463
3	0.003	4.01197	0.111483
4	0.004	3.85082	0.150858
5	0.005	3.72837	0.182649
6	0.006	3.7885	0.22017
7	0.007	3.63982	0.254107
8	0.008	3.4888	0.279286
9	0.009	3.3465	0.311244
10	0.01	3.42057	0.342842

Temperature = -40 ^{o}C

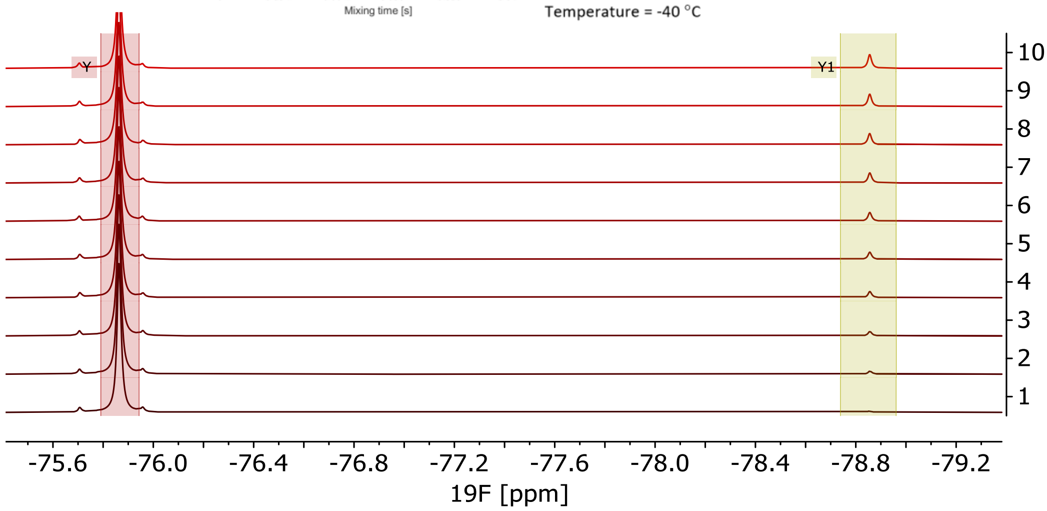

Figure S50: Raw ^{19}F EXSY data for the α-triflate dissociation in the corresponding α-triflate.

Selective 1D ^{19}F EXSY data at -70 ^{o}C

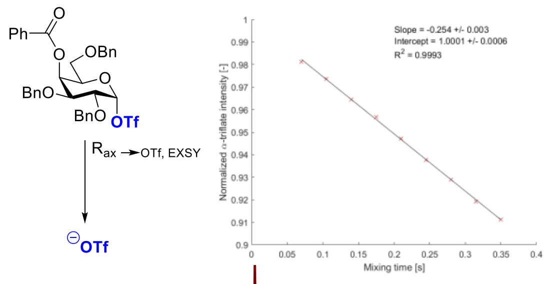

Spectrum number	Mixing Time [s]	α-triflate (-75.87 ppm) resonance intensity	¯OTf (-78.91 ppm) resonance intensity
1	0.07	6.27864	0.119609
2	0.105	5.53051	0.150218
3	0.14	4.87103	0.178826
4	0.175	4.30734	0.19489
5	0.21	3.78774	0.211211
6	0.245	3.35707	0.22355
7	0.28	2.97965	0.22824
8	0.315	2.61864	0.230217
9	0.35	2.3615	0.230034

Temperature = -70 ^{o}C

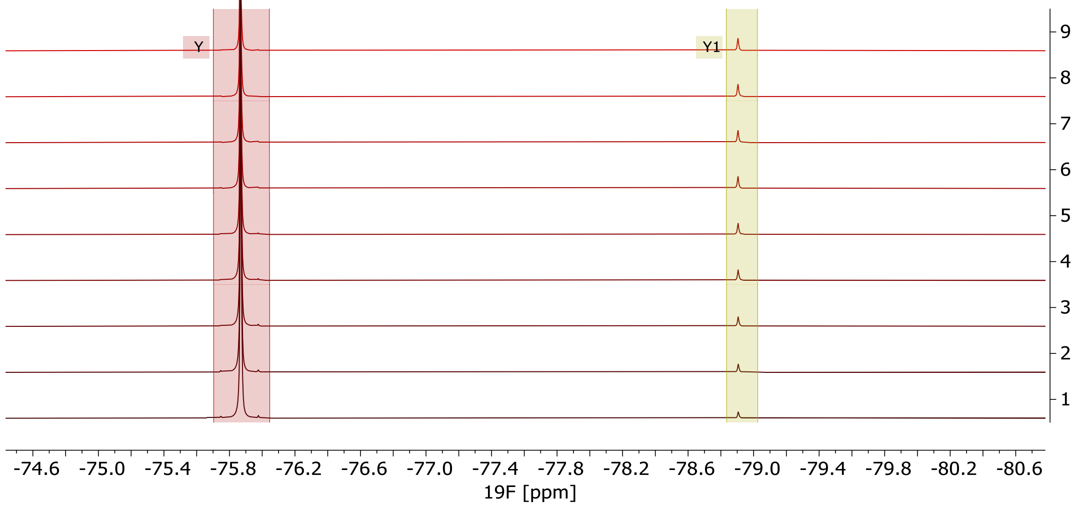

Figure S51: Raw ^{19}F EXSY data for the α-triflate dissociation in the corresponding α-triflate.

Selective 1D ^{19}F EXSY data at -60 °C

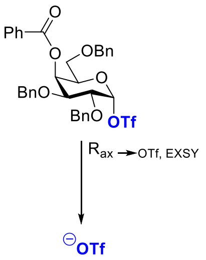

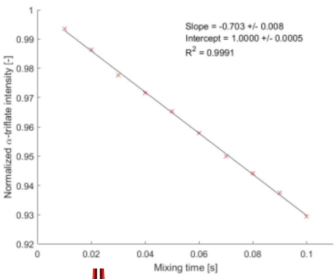

Spectrum number	Mixing Time [s]	α-triflate (-75.87 ppm) resonance intensity	-OTf (-78.91 ppm) resonance intensity
1	0.01	8.70537	0.055928
2	0.02	8.31719	0.115279
3	0.03	7.86743	0.179125
4	0.04	7.83246	0.22916
5	0.05	7.37347	0.265007
6	0.06	7.24282	0.318358
7	0.07	6.93653	0.365338
8	0.08	6.63679	0.392697
9	0.09	6.53751	0.435739
10	0.1	6.19324	0.470188

Temperature = -60 °C

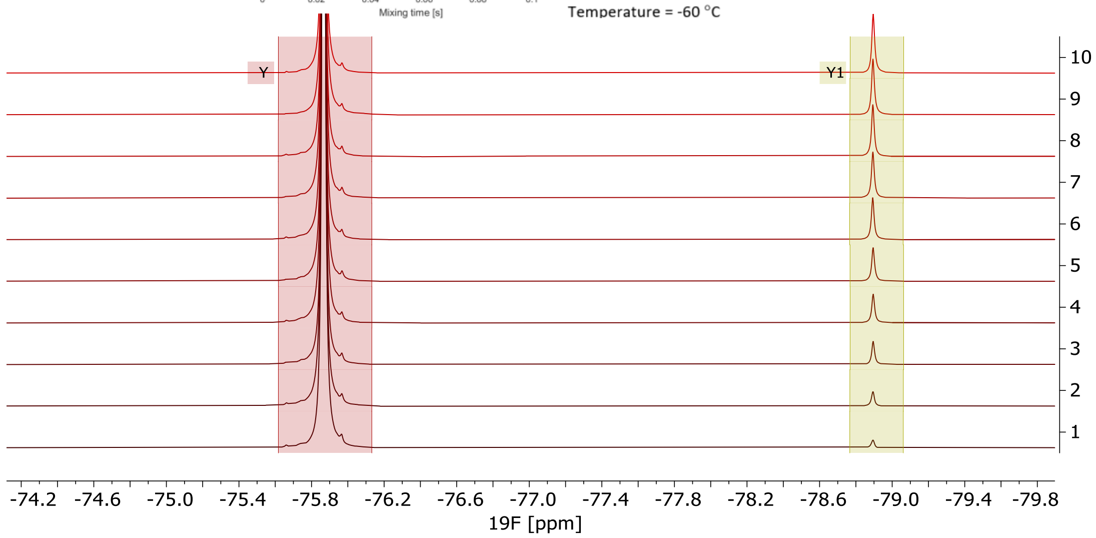

Figure S52: Raw ^{19}F EXSY data for the α-triflate dissociation in the corresponding α-triflate.

Selective 1D ^{19}F EXSY data at -50 °C

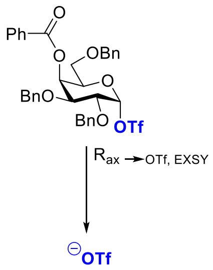

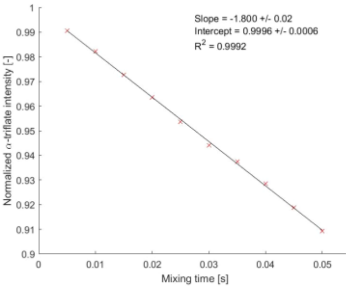

Spectrum number	Mixing Time [s]	α-triflate (-75.87 ppm) resonance intensity	-OTf (-78.91 ppm) resonance intensity
1	0.005	8.58156	0.082022
2	0.01	8.69267	0.157624
3	0.015	8.47548	0.238058
4	0.02	8.11834	0.306601
5	0.025	7.73554	0.375577
6	0.03	7.55071	0.446807
7	0.035	7.62792	0.508486
8	0.04	7.35019	0.565197
9	0.045	7.09125	0.627431
10	0.05	6.79438	0.678714

Temperature = -50 °C

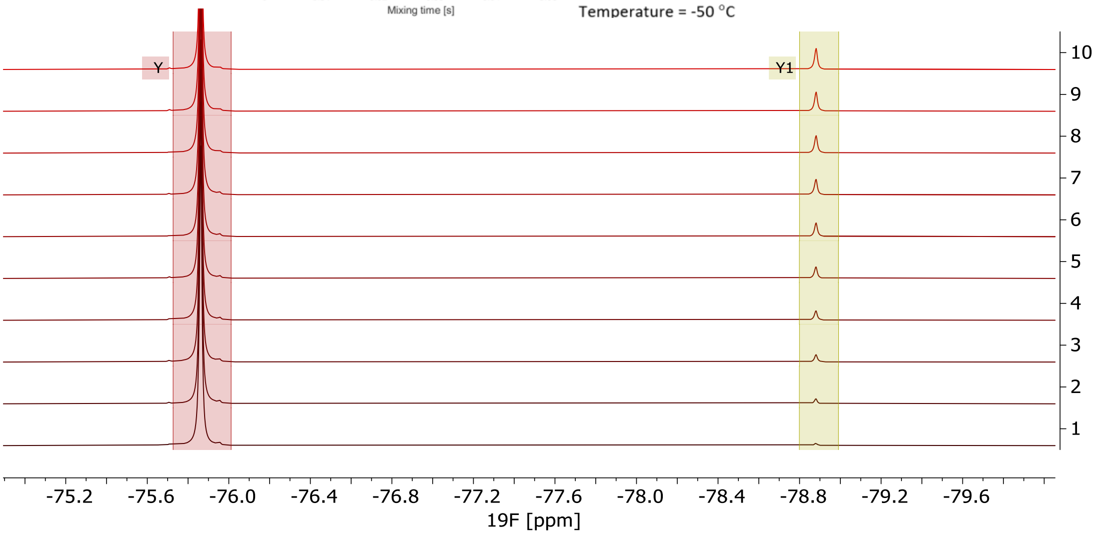

Figure S53: Raw ^{19}F EXSY data for the α-triflate dissociation in the corresponding α-triflate.

Selective 1D ^{19}F EXSY data at -40 °C

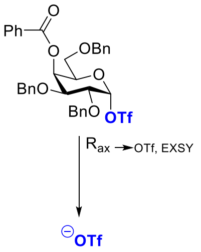

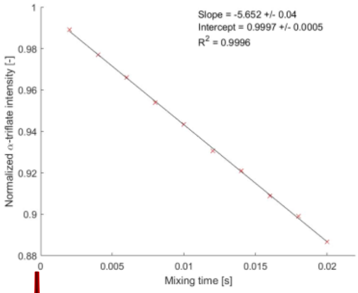

Spectrum number	Mixing Time [s]	α-triflate (-75.87 ppm) resonance intensity	$^{-}$OTf (-78.91 ppm) resonance intensity
1	0.002	5.51661	0.061016
2	0.004	5.18666	0.121569
3	0.006	5.19715	0.182898
4	0.008	4.89655	0.236826
5	0.01	4.92243	0.295696
6	0.012	4.62974	0.346161
7	0.014	4.64245	0.398452
8	0.016	4.38863	0.44021
9	0.018	4.37285	0.490856
10	0.02	4.17221	0.533235

Temperature = -40 °C

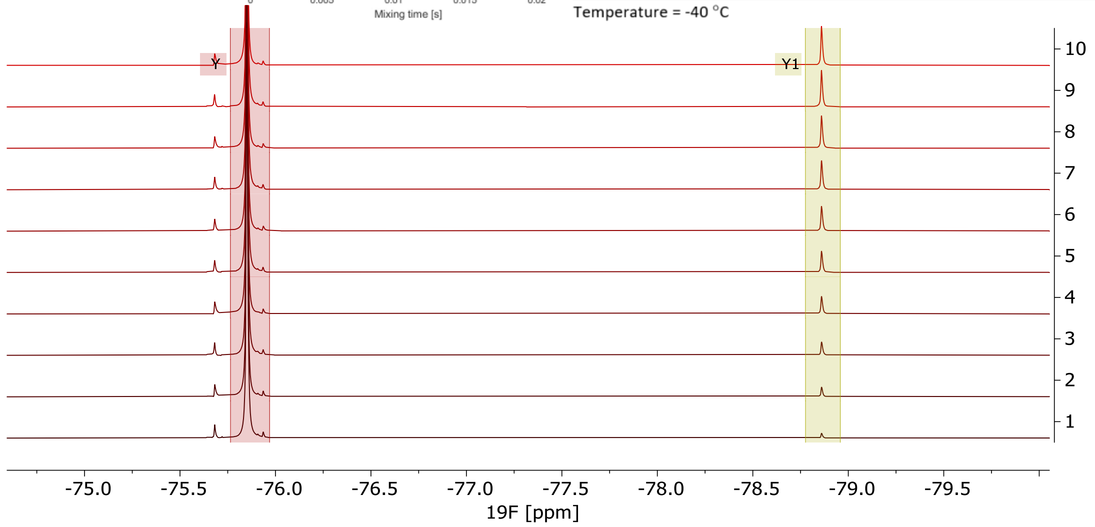

Figure S54: Raw ^{19}F EXSY data for the α-triflate dissociation in the corresponding α-triflate.

Selective 1D ^{19}F EXSY data at -80 °C

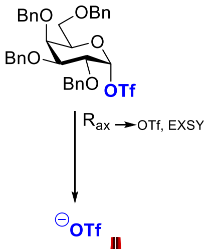

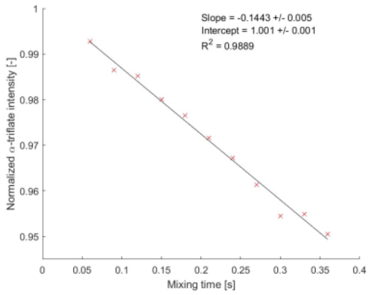

Spectrum number	Mixing Time [s]	α-triflate (-76.06 ppm) resonance intensity	$^{-}$OTf (-78.91 ppm) resonance intensity
1	0.06	2.83882	0.020795
2	0.09	2.56476	0.035103
3	0.12	2.30596	0.034681
4	0.15	2.09154	0.042678
5	0.18	1.91244	0.045956
6	0.21	1.6943	0.049605
7	0.24	1.5442	0.05238
8	0.27	1.41202	0.056841
9	0.3	1.28196	0.061117
10	0.33	1.16019	0.054777
11	0.36	1.05509	0.054928

Temperature = -80 °C

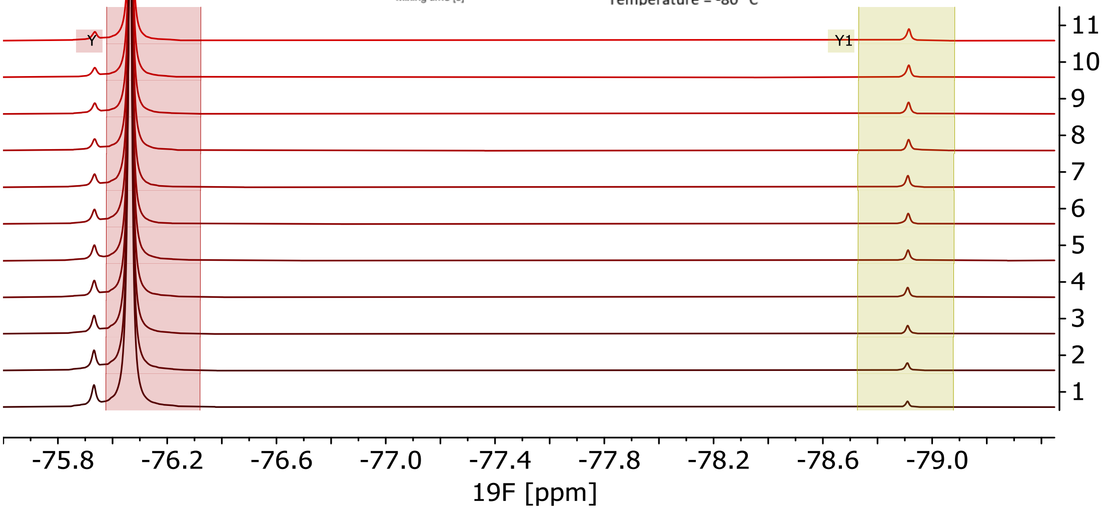

Figure S55: Raw ^{19}F EXSY data for the α-triflate dissociation in the corresponding α-triflate.

Selective 1D ^{19}F EXSY data at -70 ^{o}C

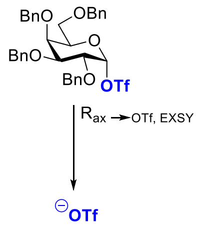

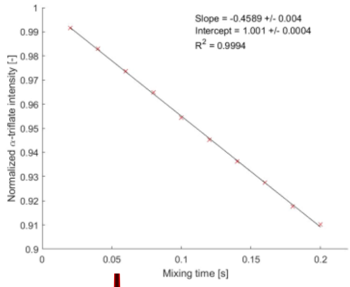

Spectrum number	Mixing Time [s]	α-triflate (-76.06 ppm) resonance intensity	$^{-}$OTf (-78.91 ppm) resonance intensity
1	0.02	3.93208	0.033701
2	0.04	3.67069	0.063292
3	0.06	3.38765	0.091873
4	0.08	3.15869	0.114673
5	0.1	2.94197	0.140916
6	0.12	2.73885	0.158914
7	0.14	2.54511	0.173335
8	0.16	2.36374	0.184908
9	0.18	2.21066	0.198325
10	0.2	2.06764	0.203719

Temperature = -70 ^{o}C

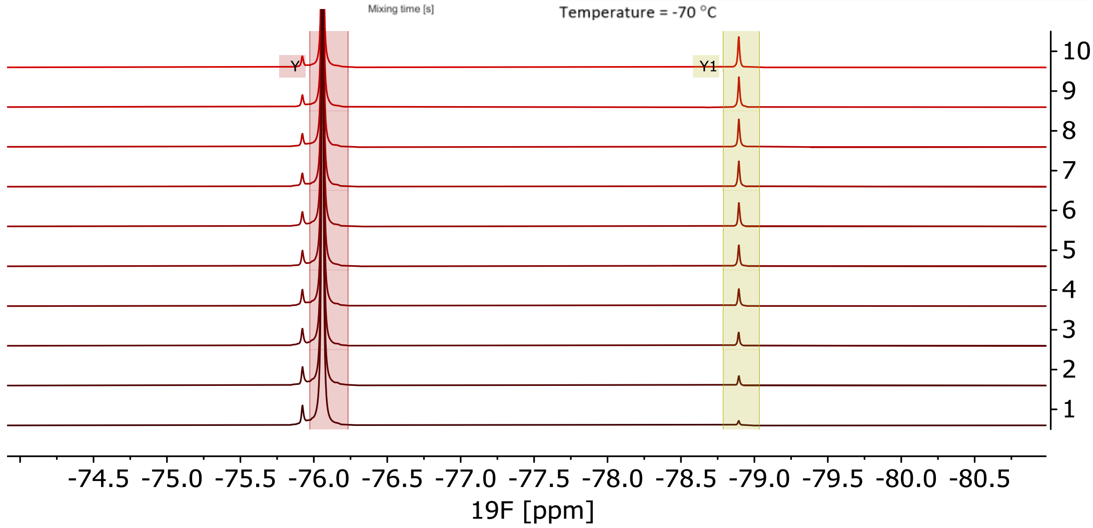

Figure S56: Raw ^{19}F EXSY data for the α-triflate dissociation in the corresponding α-triflate.

Selective 1D ^{19}F EXSY data at -60 ^{o}C

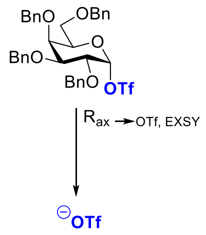

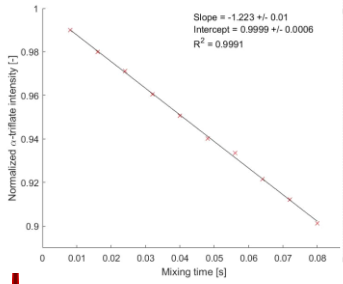

Spectrum number	Mixing Time [s]	α-triflate (-76.06 ppm) resonance intensity	$^{-}$OTf (-78.91 ppm) resonance intensity
1	0.008	4.98841	0.050246
2	0.016	4.97245	0.101147
3	0.024	4.77469	0.141832
4	0.032	4.51457	0.185103
5	0.04	4.29775	0.22297
6	0.048	4.18277	0.265919
7	0.056	4.12178	0.293188
8	0.064	3.9291	0.334025
9	0.072	3.75374	0.361589
10	0.08	3.59112	0.393699

Temperature = -60 ^{o}C

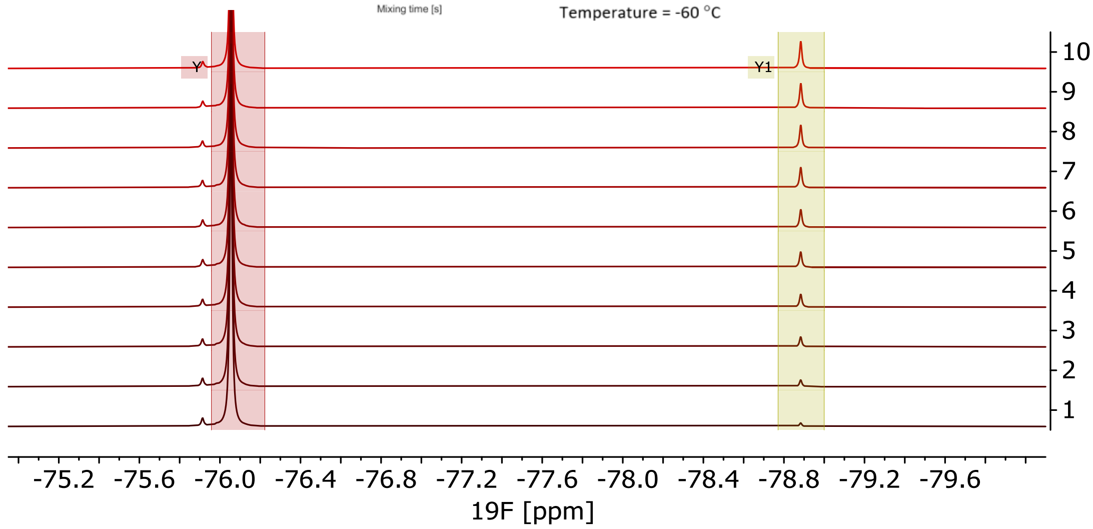

Figure S57: Raw ^{19}F EXSY data for the α-triflate dissociation in the corresponding α-triflate.

Selective 1D ^{19}F EXSY data at -50 ^{o}C

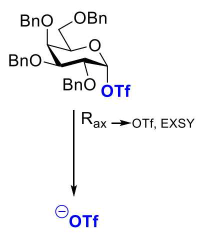

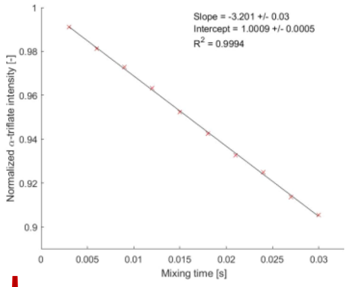

Spectrum number	Mixing Time [s]	α-triflate (-76.06 ppm) resonance intensity	$^{-}$OTf (-78.91 ppm) resonance intensity
1	0.003	5.75385	0.051398
2	0.006	5.44007	0.103769
3	0.009	5.3631	0.149373
4	0.012	5.13334	0.196076
5	0.015	5.02356	0.250777
6	0.018	4.84732	0.295079
7	0.021	4.67754	0.3375
8	0.024	4.58939	0.371851
9	0.027	4.37145	0.4129
10	0.03	4.32992	0.451727

Temperature = -50 ^{o}C

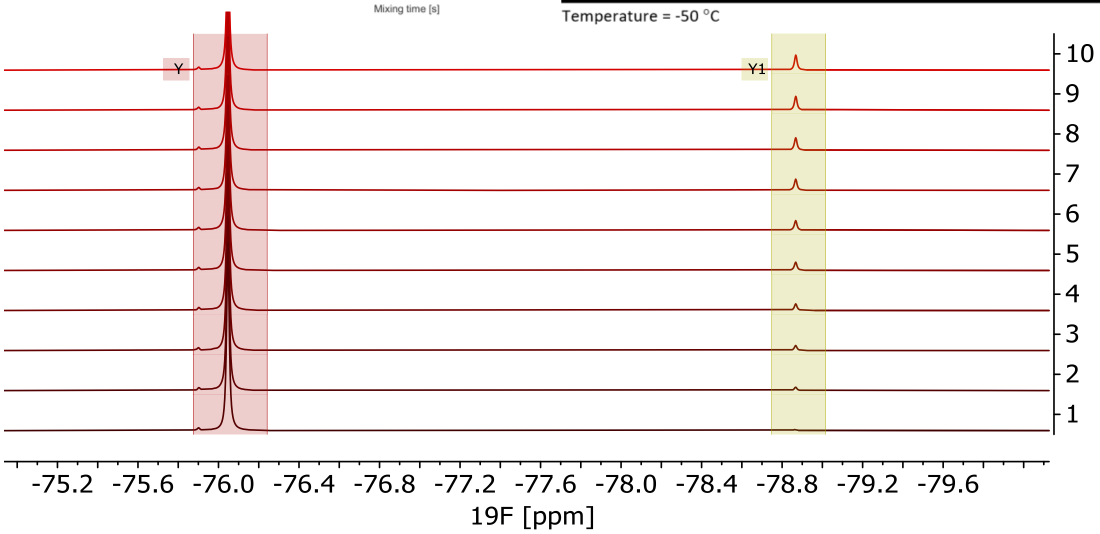

Figure S58: Raw ^{19}F EXSY data for the α-triflate dissociation in the corresponding α-triflate.

EXSY at different concentrations ⁻OTf

Selective 1D ^{19}F EXSY data at [OTf] = 0.032 M

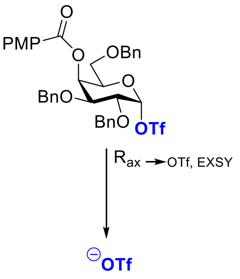

Spectrum number	Mixing Time [s]	α-triflate (-76.06 ppm) resonance intensity	⁻OTf (-78.91 ppm) resonance intensity
1	0.007	29001	247.618
2	0.014	28573.4	460.589
3	0.021	28202.7	758.251
4	0.028	27553.3	952.389
5	0.035	26878.3	1142.41
6	0.042	26074.3	1407.06
7	0.049	25332.2	1578.15
8	0.056	24499.4	1764.94
9	0.063	23830.7	1943.88
10	0.07	23206.9	2124.48

[⁻OTf] = 0.034 M

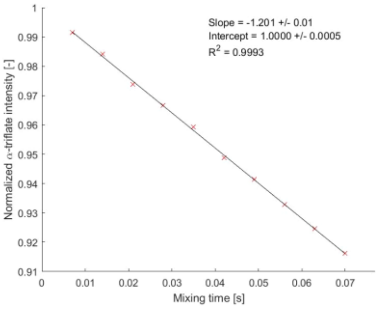

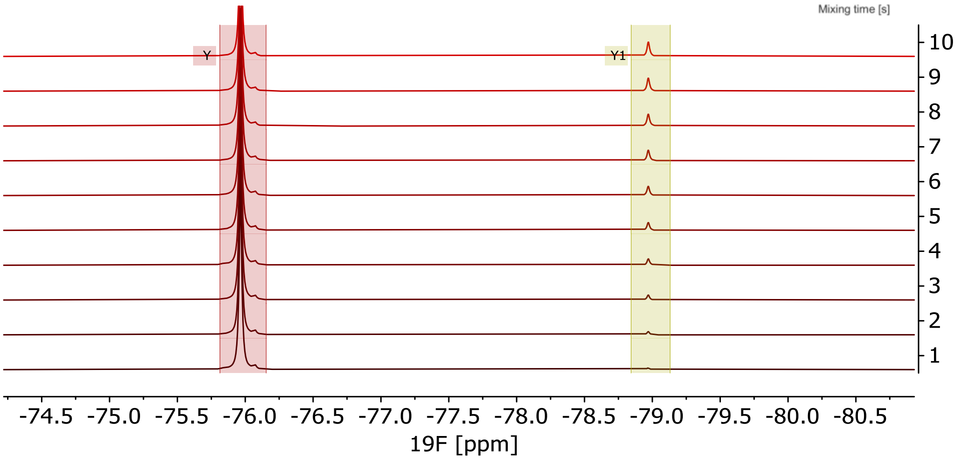

Figure S59: Raw ^{19}F EXSY data for the α-triflate dissociation in the corresponding α-triflate.

Selective 1D ^{19}F EXSY data at [OTf] = 0.060 M

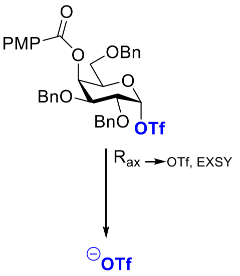

Spectrum number	Mixing Time [s]	α-triflate (-76.06 ppm) resonance intensity	⁻OTf (-78.91 ppm) resonance intensity
1	0.0035	24731.6	293.82
2	0.007	24352.8	529.349
3	0.0105	23946.3	772.104
4	0.014	23566.7	1120.52
5	0.0175	23174.8	1316.28
6	0.021	22854.4	1609.8
7	0.0245	22308.2	1774.67
8	0.028	22053.4	2041.98
9	0.0315	21570.2	2269.15
10	0.035	21192.4	2550.59

[⁻OTf] = 0.060 M

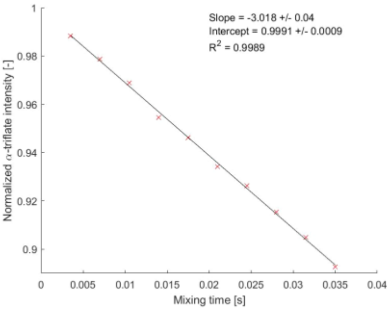

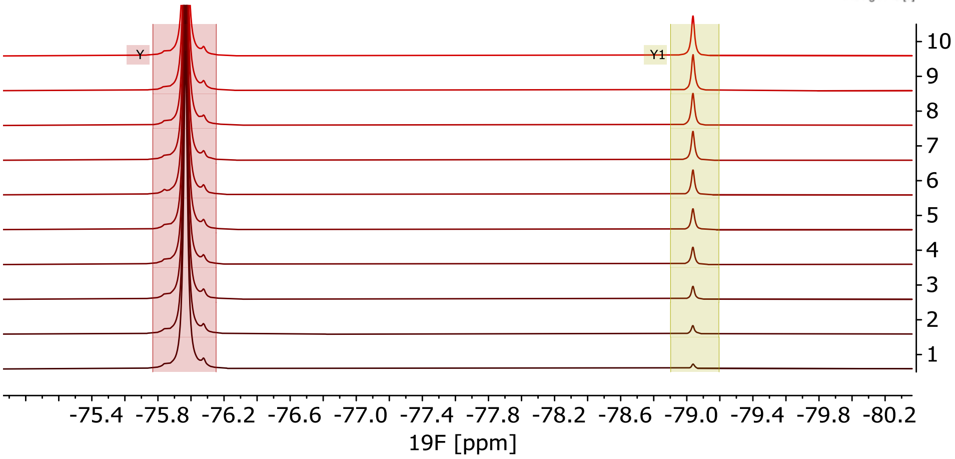

Figure S60: Raw ^{19}F EXSY data for the α-triflate dissociation in the corresponding α-triflate.

Selective 1D ^{19}F EXSY data at [OTf] = 0.10 M

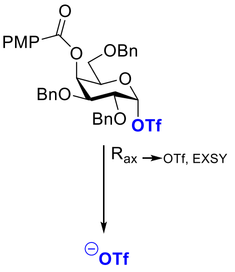

Spectrum number	Mixing Time [s]	α-triflate (-76.06 ppm) resonance intensity	$^{-}$OTf (-78.91 ppm) resonance intensity
1	0.0018	19165.9	239.861
2	0.0036	17891.6	370.685
3	0.0054	18526.6	637.302
4	0.0072	17410.2	852.828
5	0.009	17826.3	1035.36
6	0.0108	16915.7	1249.67
7	0.0126	17213.4	1485.45
8	0.0144	16122.6	1612.32
9	0.0162	16476.5	1811.71
10	0.018	15672.1	1958.32

[$^{-}$OTf] = 0.10 M

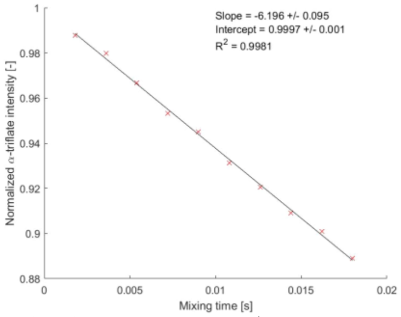

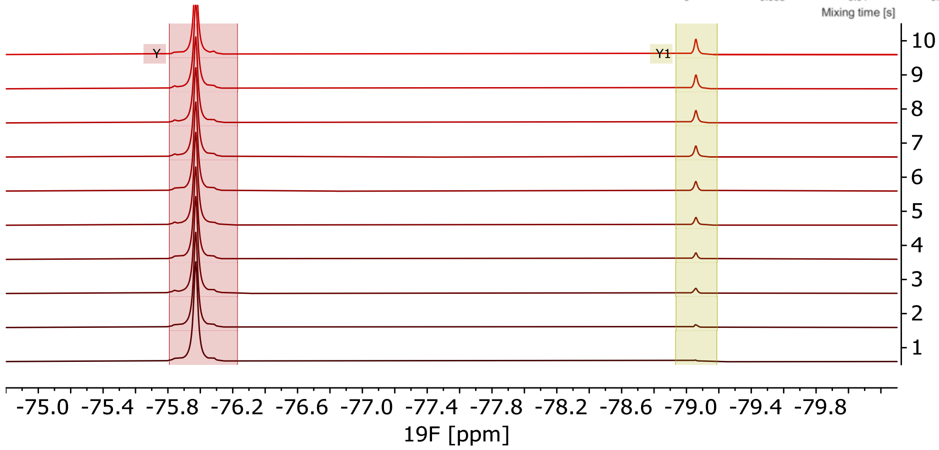

Figure S61: Raw ^{19}F EXSY data for the α-triflate dissociation in the corresponding α-triflate.

Selective 1D ^{19}F EXSY data at [OTf] = 0.16 M

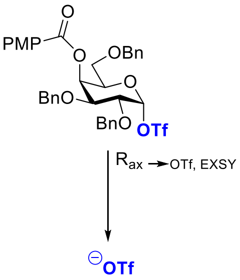

Spectrum number	Mixing Time [s]	α-triflate (-76.06 ppm) resonance intensity	$^{-}$OTf (-78.91 ppm) resonance intensity
1	0.001	13920.3	146.705
2	0.002	13939.9	220.79
3	0.003	13442.6	441.237
4	0.004	13174.8	539.117
5	0.005	13212.5	617.239
6	0.006	13070.3	727.517
7	0.007	12619.4	831.804
8	0.008	12601.5	992.911
9	0.009	12686.4	1129.89
10	0.01	12233.1	1176.91

[$^{-}$OTf] = 0.16 M

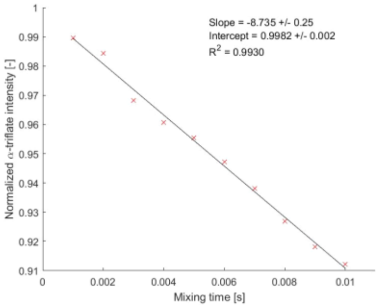

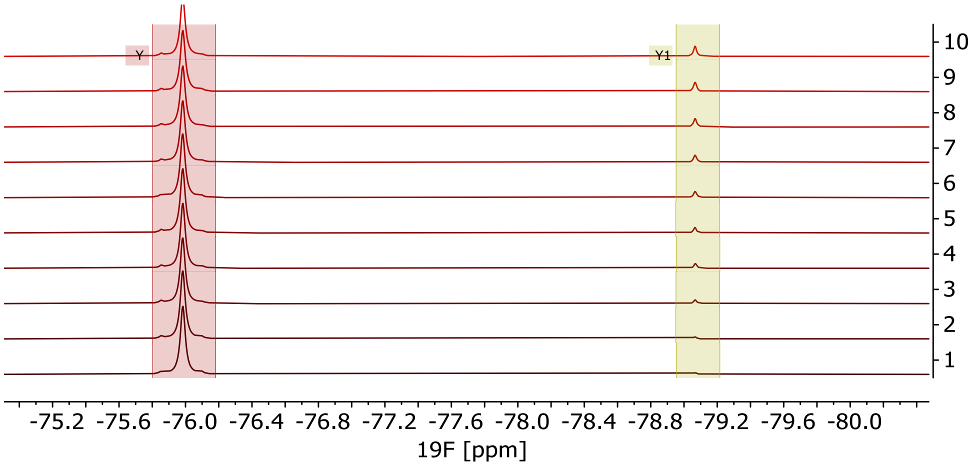

Figure S62: Raw ^{19}F EXSY data for the α-triflate dissociation in the corresponding α-triflate.

Selective 1D ^{19}F EXSY data at [OTf] = 0.026 M

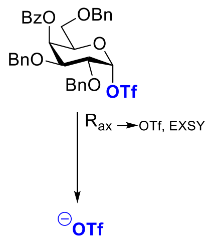

Spectrum number	Mixing Time [s]	α-triflate (-75.95 ppm) resonance intensity	-OTf (-79.06 ppm) resonance intensity
1	0.014	33595.1	303.207
2	0.028	32505.8	621.112
3	0.042	30709	917.686
4	0.056	29148.6	1213.77
5	0.07	27851.7	1440.55
6	0.084	26664	1749.71
7	0.098	25464.8	1906.59
8	0.112	24339.9	2089.56
9	0.126	23104.9	2280.67
10	0.14	22232.2	2496.14

[-OTf] = 0.026 M

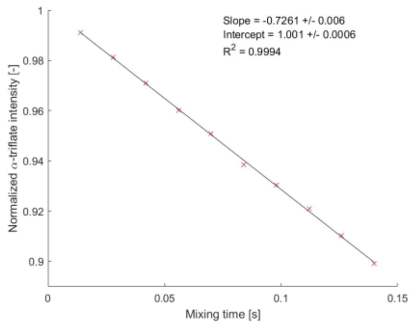

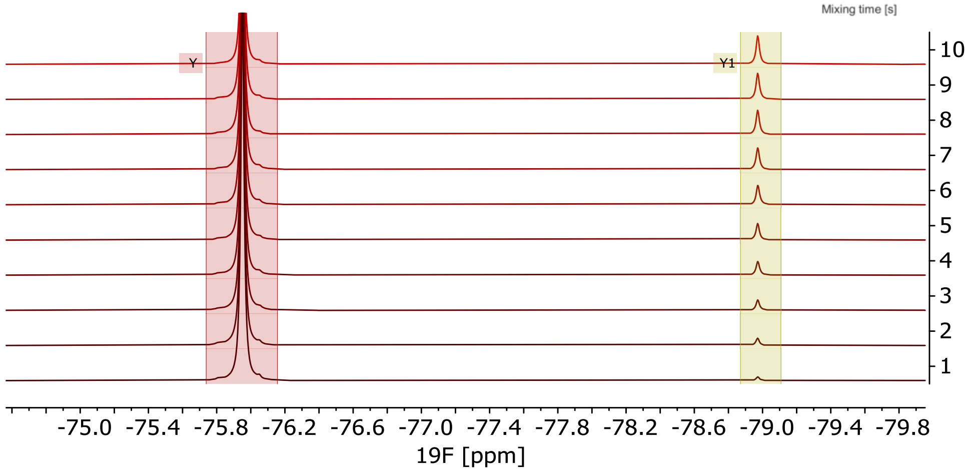

Figure S63: Raw ^{19}F EXSY data for the α-triflate dissociation in the corresponding α-triflate.

Selective 1D ^{19}F EXSY data at [OTf] = 0.061 M

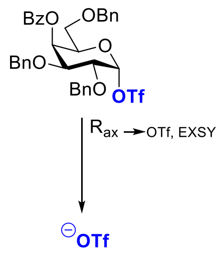

Spectrum number	Mixing Time [s]	α-triflate (-75.95 ppm) resonance intensity	-OTf (-79.06 ppm) resonance intensity
1	0.004	27641.4	305.906
2	0.008	27278.2	577.179
3	0.012	26882.5	887.875
4	0.016	26488.5	1162.51
5	0.02	25964.6	1384.2
6	0.024	25598.3	1643.36
7	0.028	24900.3	1911.68
8	0.032	24247.3	2192.88
9	0.036	23569.6	2384.2
10	0.04	23042.7	2632.93

[-OTf] = 0.061 M

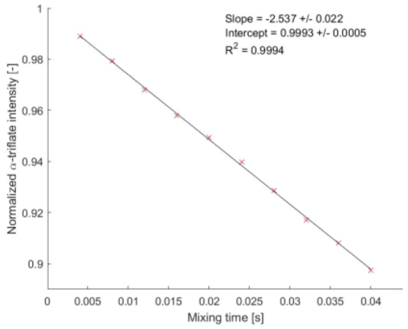

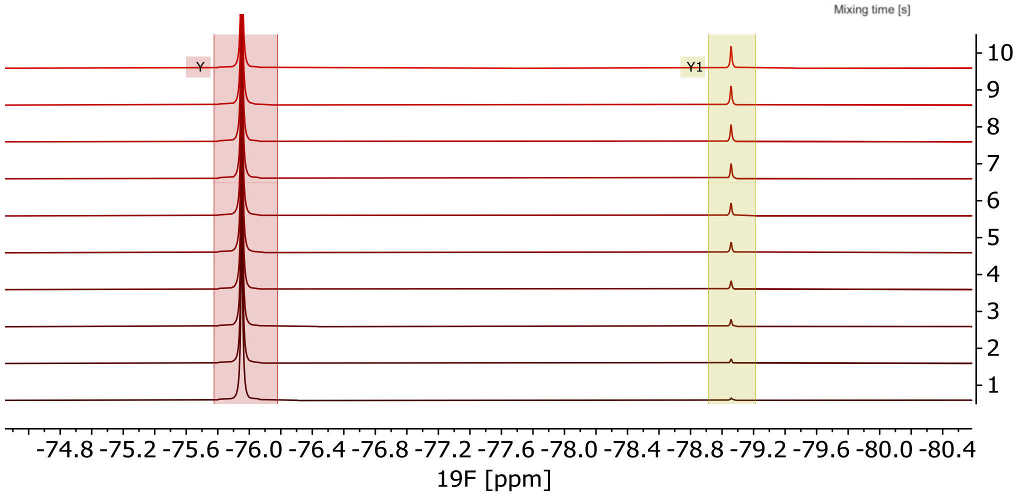

Figure S64: Raw ^{19}F EXSY data for the α-triflate dissociation in the corresponding α-triflate.

Selective 1D ^{19}F EXSY data at [OTf] = 0.11 M

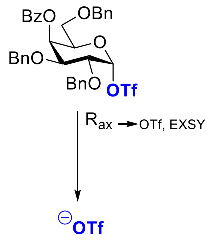

Spectrum number	Mixing Time [s]	α-triflate (-75.95 ppm) resonance intensity	$^{-}$OTf (-79.06 ppm) resonance intensity
1	0.002	22010.1	232.235
2	0.004	20741.8	413.295
3	0.006	21152.1	680.02
4	0.008	20228.1	796.522
5	0.01	19972.2	1088.91
6	0.012	19728.3	1318.78
7	0.014	19251.1	1578.35
8	0.016	19398.6	1729.84
9	0.018	18315.3	1934.67
10	0.02	18617.8	2147.53

[$^{-}$OTf] = 0.11 M

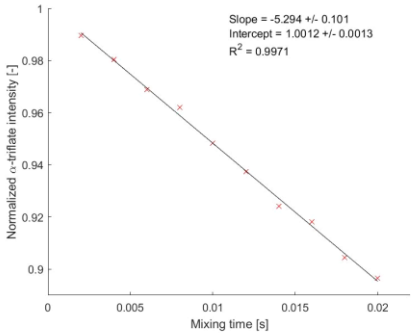

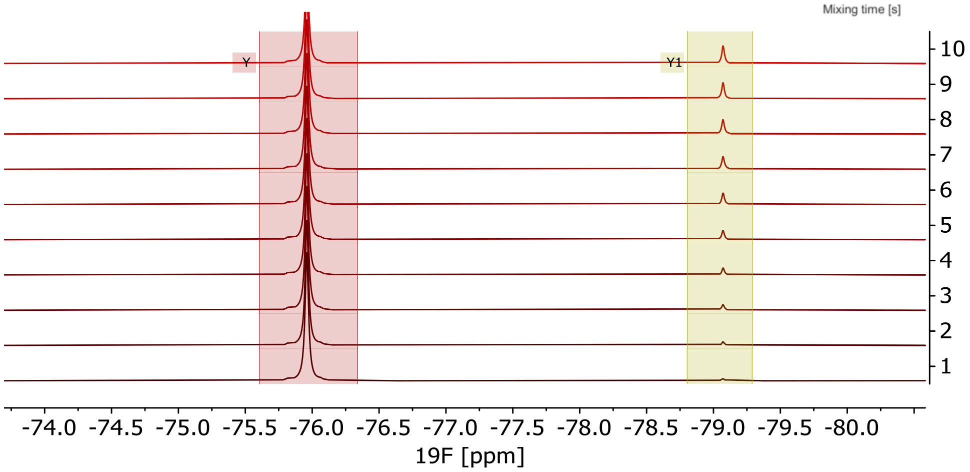

Figure S65: Raw ^{19}F EXSY data for the α-triflate dissociation in the corresponding α-triflate.

Selective 1D ^{19}F EXSY data at [OTf] = 0.18 M

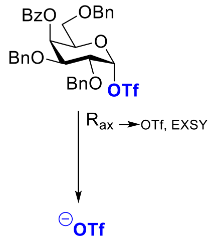

Spectrum number	Mixing Time [s]	α-triflate (-75.95 ppm) resonance intensity	$^{-}$OTf (-79.06 ppm) resonance intensity
1	0.001	15108.3	192.718
2	0.002	14964	254.193
3	0.003	14124.3	481.897
4	0.004	14221.5	652.311
5	0.005	14483.3	710.002
6	0.006	13917.5	857.993
7	0.007	13467.7	1024.76
8	0.008	13646.1	1099.76
9	0.009	13352.2	1360.45
10	0.01	12747.1	1401.35

[$^{-}$OTf] = 0.18 M

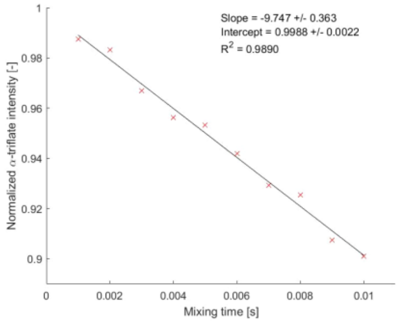

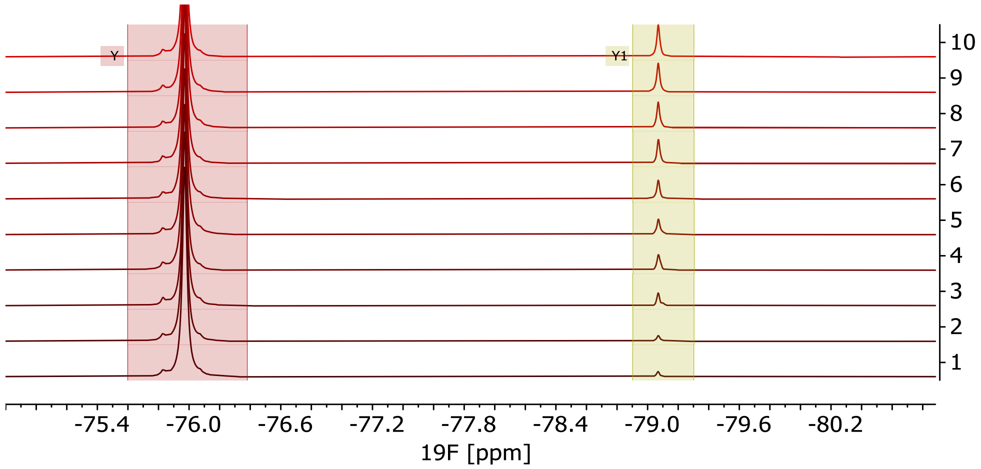

Figure S66: Raw ^{19}F EXSY data for the α-triflate dissociation in the corresponding α-triflate.

Selective 1D ^{19}F EXSY data at [OTf] = 0.026 M

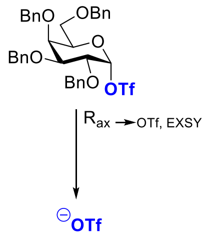

Spectrum number	Mixing Time [s]	α-triflate (-76.06 ppm) resonance intensity	⁻OTf (-78.91 ppm) resonance intensity
1	0.008	32303.5	288.752
2	0.016	30683.4	558.604
3	0.024	30410.2	827.235
4	0.032	29493.6	1100.52
5	0.04	28700.6	1327.41
6	0.048	28035.1	1540.7
7	0.056	27326.2	1731.89
8	0.064	26678.9	1947.96
9	0.072	25937	2145.84
10	0.08	25332.6	2360.7

[⁻OTf] = 0.026M

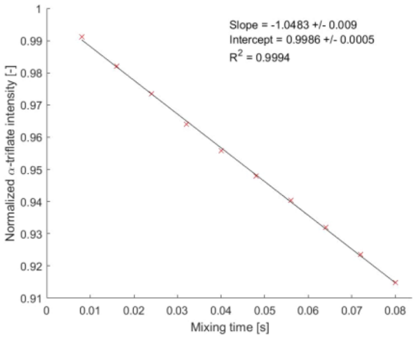

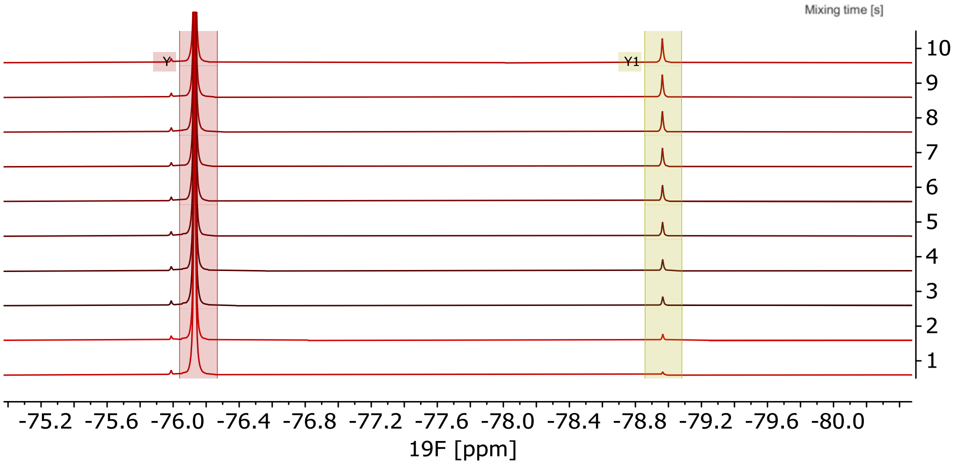

Figure S67: Raw ^{19}F EXSY data for the α-triflate dissociation in the corresponding α-triflate.

Selective 1D ^{19}F EXSY data at [OTf] = 0.059 M

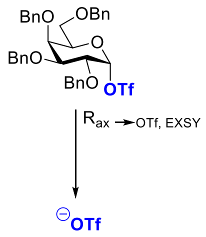

Spectrum number	Mixing Time [s]	α-triflate (-76.06 ppm) resonance intensity	⁻OTf (-78.91 ppm) resonance intensity
1	0.003	23579.2	280.305
2	0.006	23098.2	537.187
3	0.009	22603	745.126
4	0.015	21805.8	1256.25
5	0.018	21517.2	1511.96
6	0.021	21138.6	1748.86
7	0.024	20771.4	1964.59
8	0.027	20325.5	2226.63
9	0.03	20034.8	2479.46
10	0.012	21914.1	1021.09

[⁻OTf] = 0.059 M

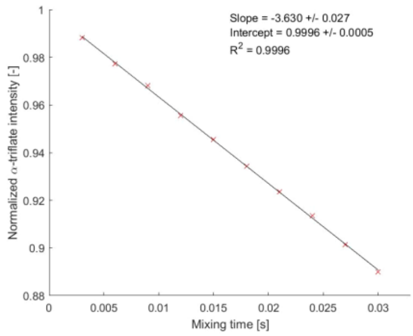

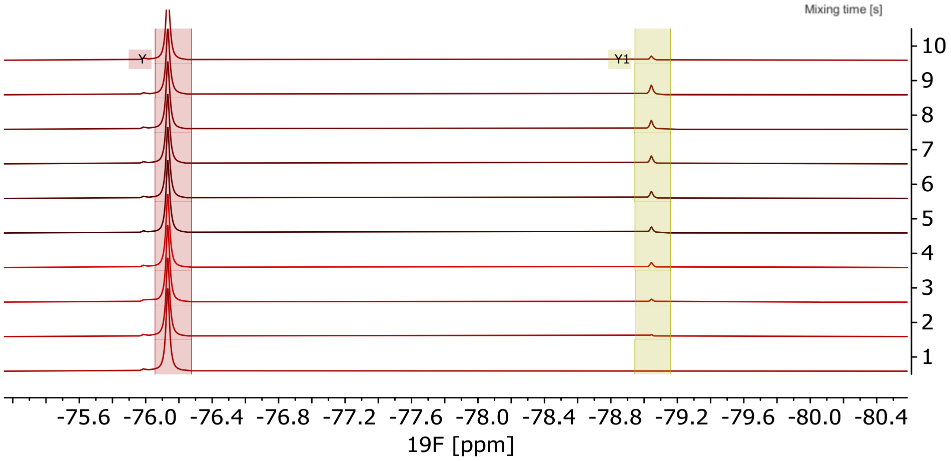

Figure S68: Raw ^{19}F EXSY data for the α-triflate dissociation in the corresponding α-triflate.

Selective 1D ^{19}F EXSY data at [OTf] = 0.11 M

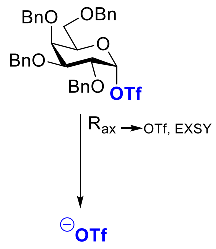

Spectrum number	Mixing Time [s]	α-triflate (-76.06 ppm) resonance intensity	$^{-}$OTf (-78.91 ppm) resonance intensity
1	0.0016	17994.4	217.684
2	0.0032	16900.2	381.544
3	0.0048	17284.3	575.727
4	0.0064	16296	715.23
5	0.008	16579	861.002
6	0.0096	15769.7	1072.84
7	0.0112	15865.1	1273.8
8	0.0128	15268.3	1455.89
9	0.0144	15316.2	1618.98
10	0.016	14702.5	1751.12

[$^{-}$OTf] = 0.11 M

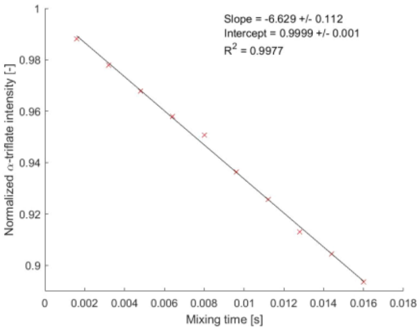

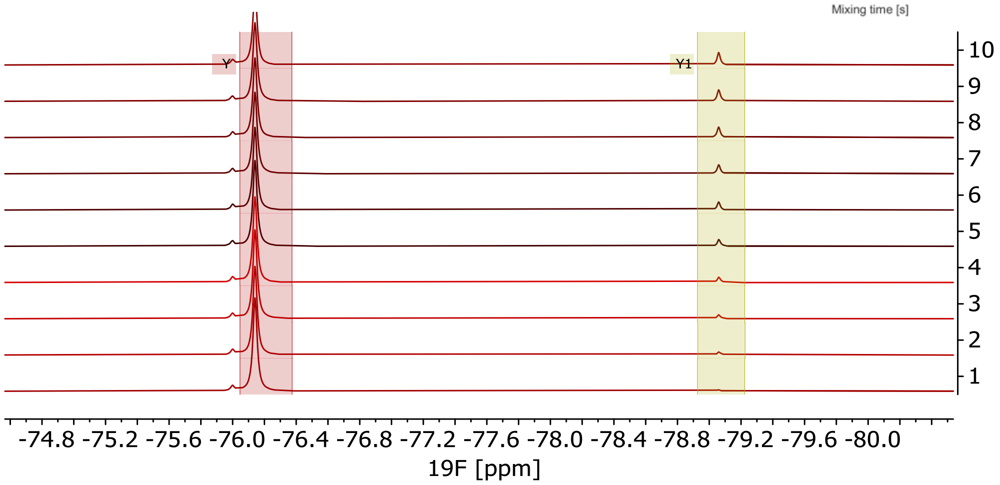

Figure S69: Raw ^{19}F EXSY data for the α-triflate dissociation in the corresponding α-triflate.

Selective 1D ^{19}F EXSY data at [OTf] = 0.17 M

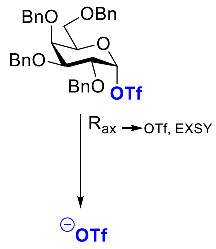

Spectrum number	Mixing Time [s]	α-triflate (-76.06 ppm) resonance intensity	$^{-}$OTf (-78.91 ppm) resonance intensity
1	0.001	10598.8	168.174
2	0.002	10332.7	296.712
3	0.003	9916.05	404.281
4	0.004	10085.3	549.515
5	0.005	9758.8	541.972
6	0.006	9504.71	776.46
7	0.007	9609.55	818.894
8	0.008	9403.2	995.855
9	0.009	8999.89	1077.76
10	0.01	9069.96	1172.1

[$^{-}$OTf] = 0.17 M

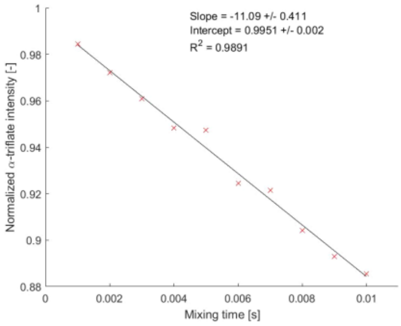

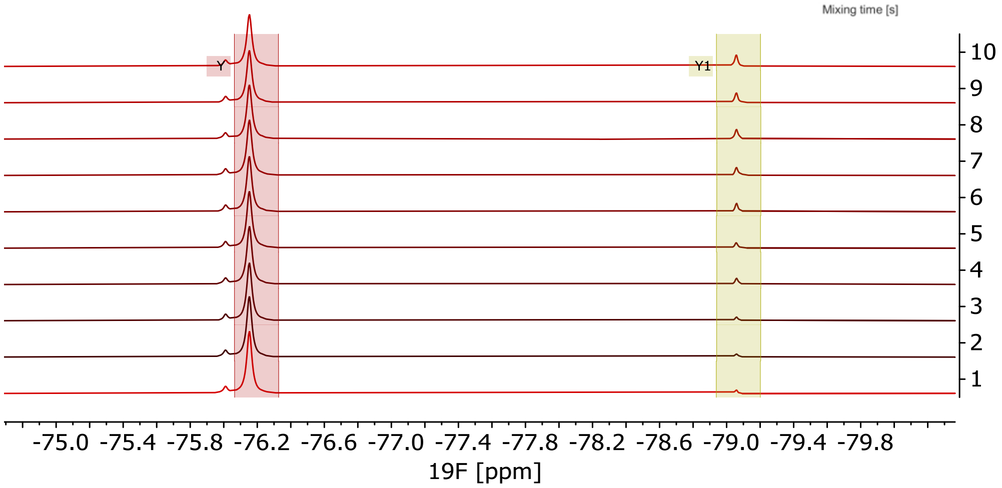

Figure S70: Raw ^{19}F EXSY data for the α-triflate dissociation in the corresponding α-triflate.

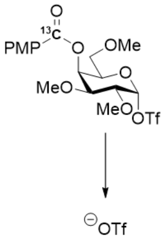

Spectrum number	Mixing Time [s]	α-triflate (-76 ppm) resonance intensity	⁻OTf (-78 ppm) resonance intensity
1	0.012	9.45227	0.088989
2	0.024	9.15599	0.168496
3	0.036	8.72292	0.236756
4	0.048	8.11433	0.310354
5	0.06	7.66259	0.366399
6	0.072	7.27664	0.418404
7	0.084	7.00061	0.474754
8	0.096	6.73236	0.520388
9	0.108	6.3496	0.574996
10	0.12	6.03828	0.638415

[⁻OTf] = 0.060M

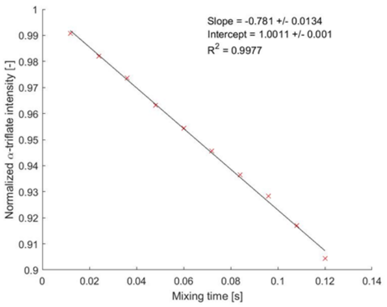

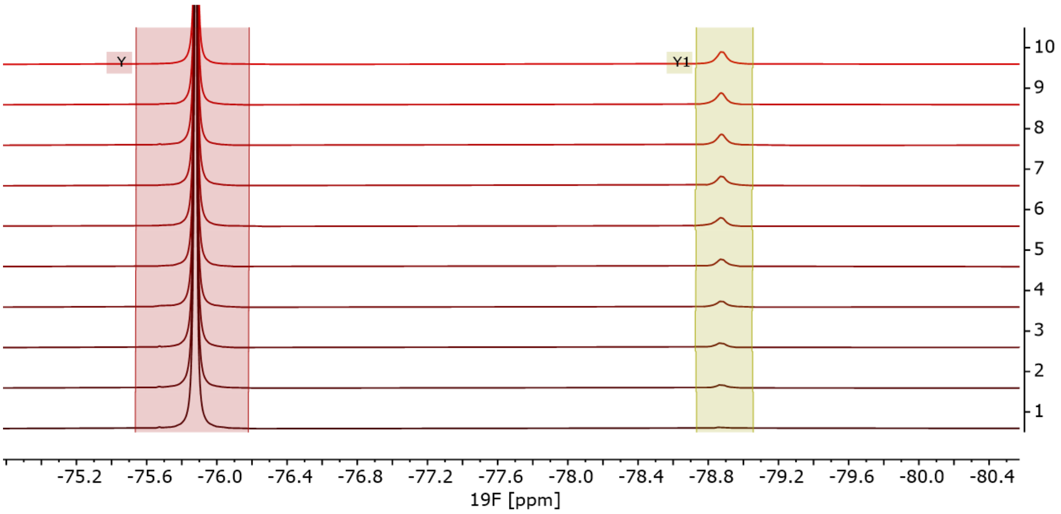

Figure S71: Raw ^{19}F EXSY data for the α-triflate dissociation in the corresponding α-triflate.

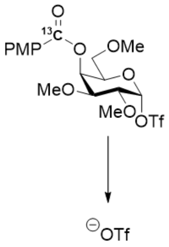

Spectrum number	Mixing Time [s]	α-triflate (-76 ppm) resonance intensity	⁻OTf (-78 ppm) resonance intensity
1	0.004	8.49683	0.061388
2	0.008	8.28889	0.174842
3	0.012	8.10756	0.233911
4	0.016	8.00733	0.319069
5	0.02	7.85806	0.386341
6	0.024	7.75043	0.456452
7	0.028	7.5273	0.498899
8	0.032	7.23728	0.613056
9	0.036	7.00039	0.682057
10	0.04	6.80346	0.6884

[⁻OTf] = 0.11 M

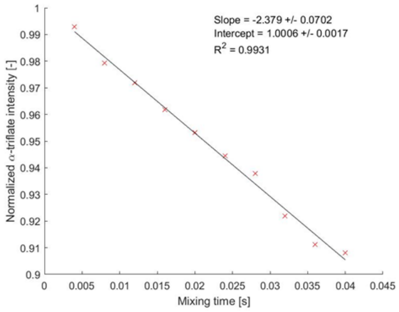

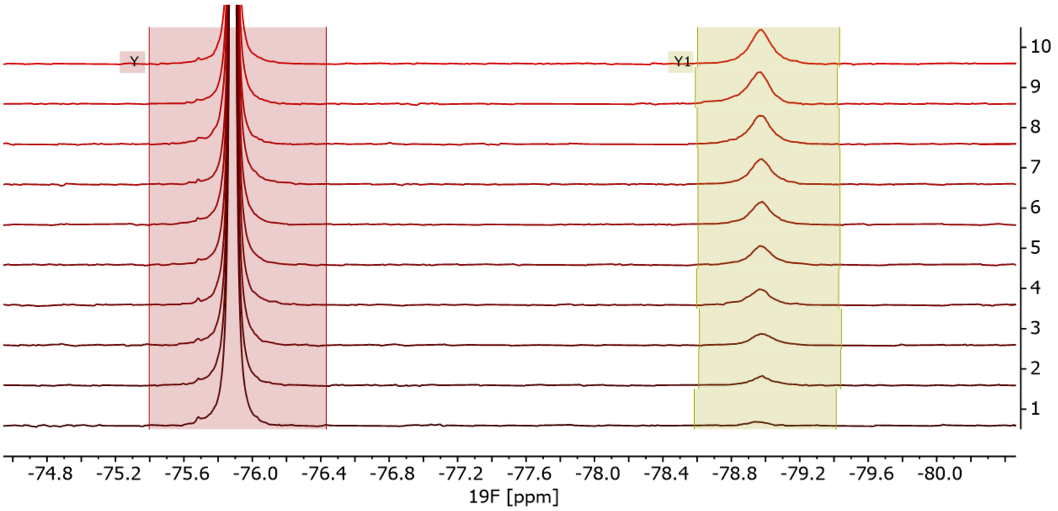

Figure S72: Raw ^{19}F EXSY data for the α-triflate dissociation in the corresponding α-triflate.

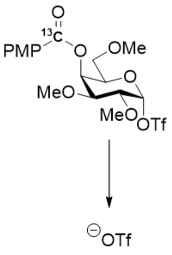

Spectrum number	Mixing Time [s]	α-triflate (-76 ppm) resonance intensity	$^{-}$OTf (-78 ppm) resonance intensity
1	0.0025	1.45886	0.016146
2	0.005	1.40953	0.038935
3	0.0075	1.37597	0.041667
4	0.01	1.36704	0.062863
5	0.0125	1.35364	0.081654
6	0.015	1.34012	0.092329
7	0.0175	1.32656	0.104577
8	0.02	1.29488	0.125817
9	0.0225	1.27231	0.140409
10	0.025	1.23242	0.144934

[$^{-}$OTf] = 0.16 M

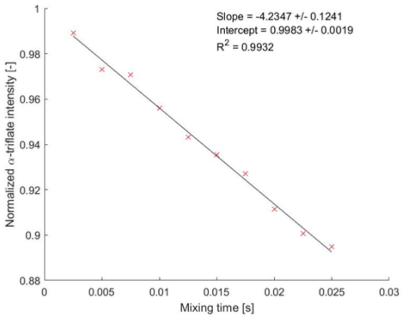

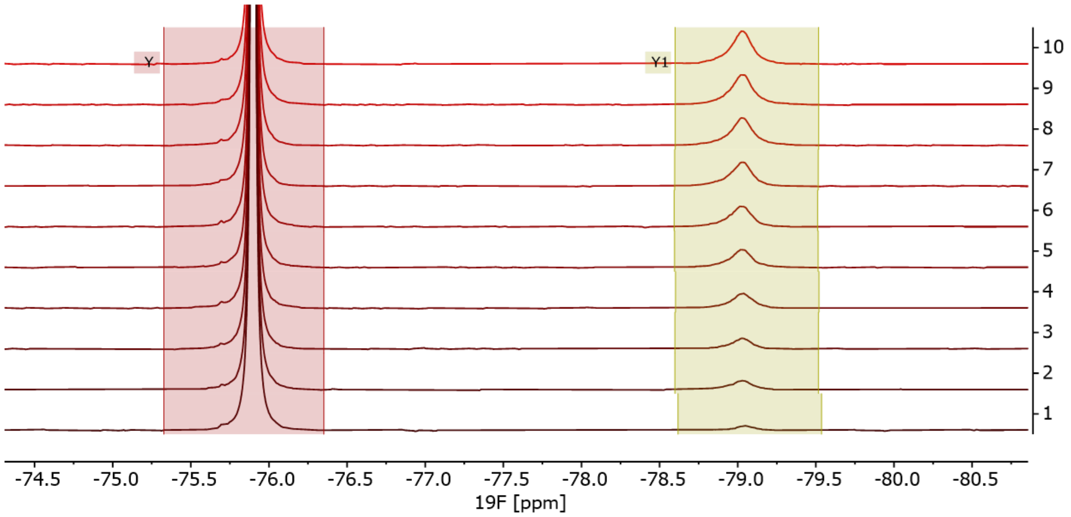

Figure S73: Raw ^{19}F EXSY data for the α-triflate dissociation in the corresponding α-triflate.

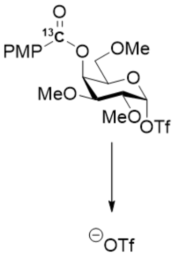

Spectrum number	Mixing Time [s]	α-triflate (-76 ppm) resonance intensity	$^{-}$OTf (-78 ppm) resonance intensity
1	0.004	9.55403	0.102676
2	0.008	9.33307	0.200219
3	0.012	9.11629	0.297143
4	0.016	8.9367	0.391337
5	0.02	8.78305	0.48534
6	0.024	8.59269	0.580212
7	0.028	8.3254	0.65272
8	0.032	8.07956	0.74382
9	0.036	7.7353	0.840771
10	0.04	7.49551	0.884198

Temperature = -70 °C

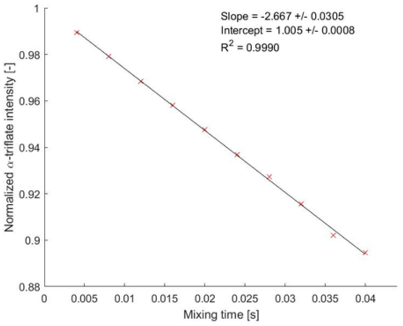

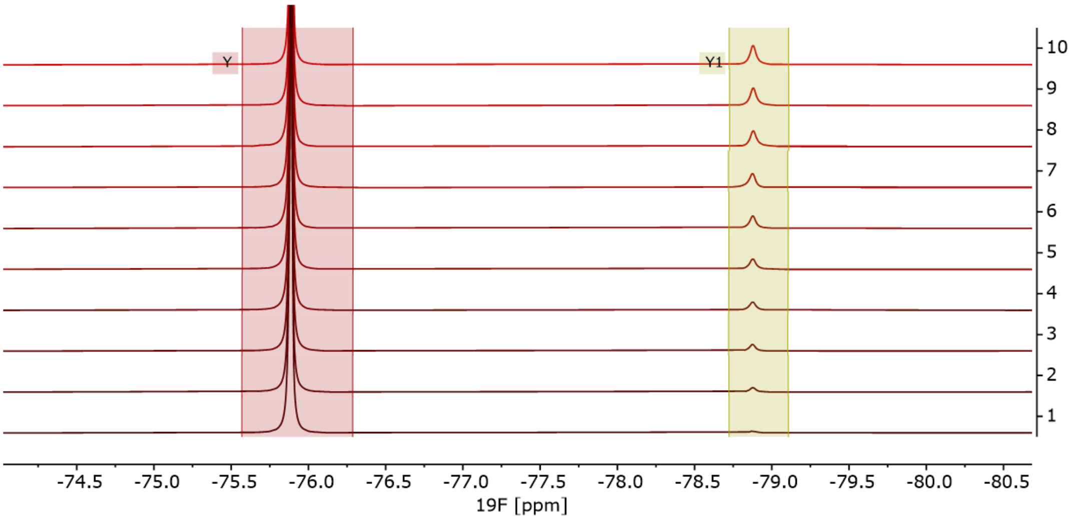

Figure S74: Raw ^{19}F EXSY data for the α-triflate dissociation in the corresponding α-triflate.

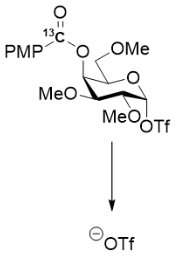

Spectrum number	Mixing Time [s]	α-triflate (-76 ppm) resonance intensity	⁻OTf (-78 ppm) resonance intensity
1	0.0014	8.70366	0.097215
2	0.0028	8.5595	0.21217
3	0.0042	8.57899	0.301247
4	0.0056	8.58367	0.4104
5	0.007	8.36608	0.49199
6	0.0084	8.08936	0.599108
7	0.0098	7.86769	0.697234
8	0.0112	7.67739	0.774483
9	0.0126	7.51629	0.860876
10	0.014	7.48537	0.93772

Temperature = -60 °C

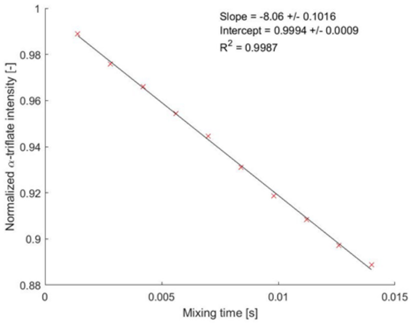

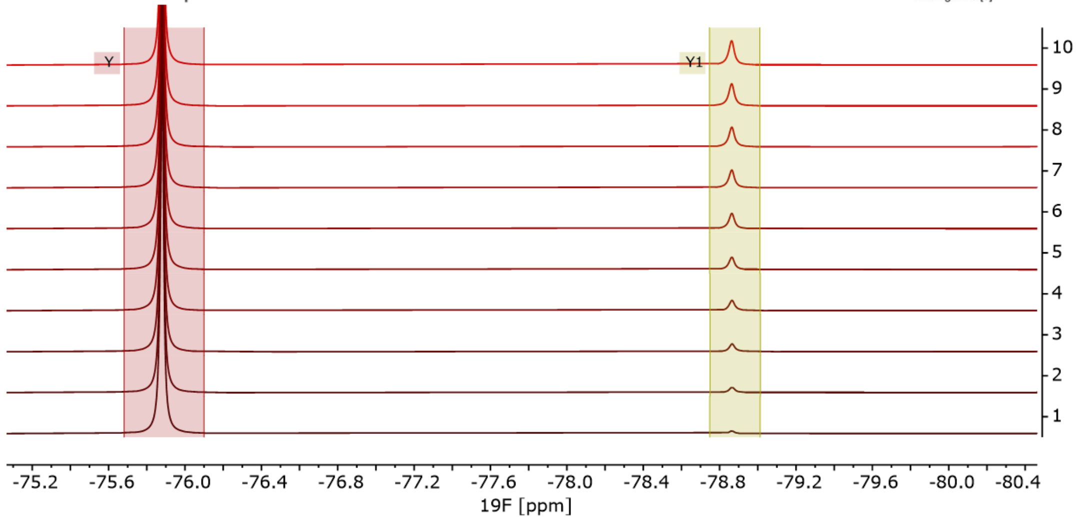

Figure S75: Raw ^{19}F EXSY data for the α-triflate dissociation in the corresponding α-triflate.

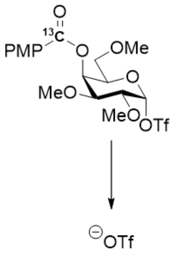

Spectrum number	Mixing Time [s]	α-triflate (-76 ppm) resonance intensity	⁻OTf (-78 ppm) resonance intensity
1	0.0005	5.87591	0.059891
2	0.001	5.60173	0.126728
3	0.0015	5.59492	0.183351
4	0.002	5.73754	0.256901
5	0.0025	5.44352	0.317154
6	0.003	5.56704	0.381362
7	0.0035	5.45069	0.448898
8	0.004	5.39362	0.512761
9	0.0045	5.53897	0.578557
10	0.005	5.22265	0.618862

Temperature = -50 °C

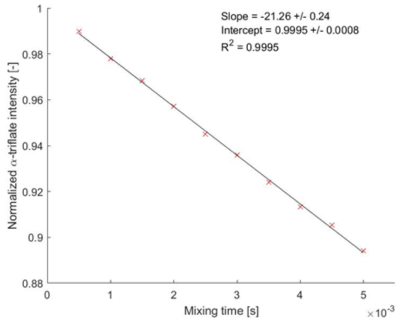

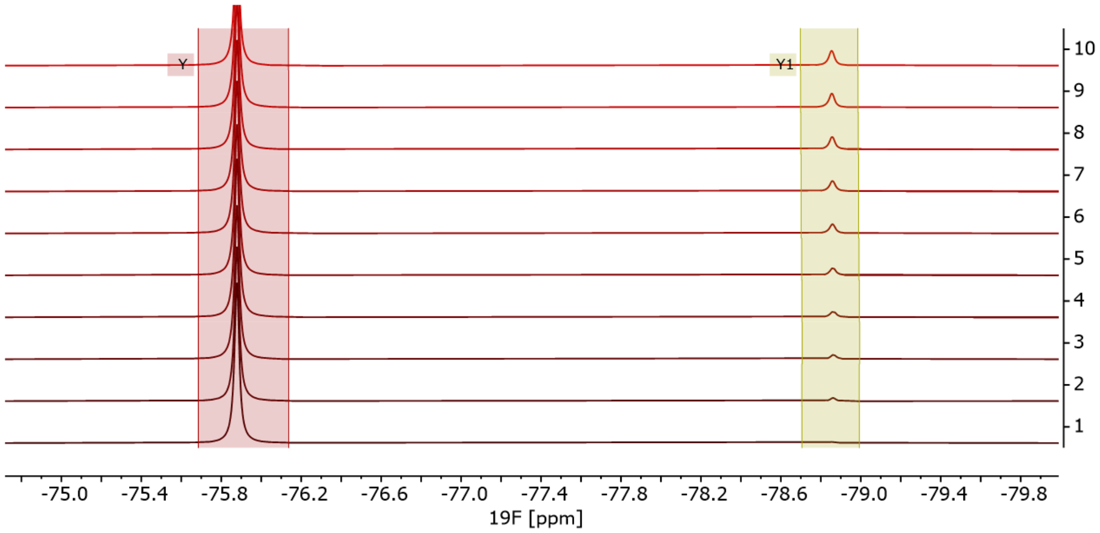

Figure S76: Raw ^{19}F EXSY data for the α-triflate dissociation in the corresponding α-triflate.

Synthesis spectra

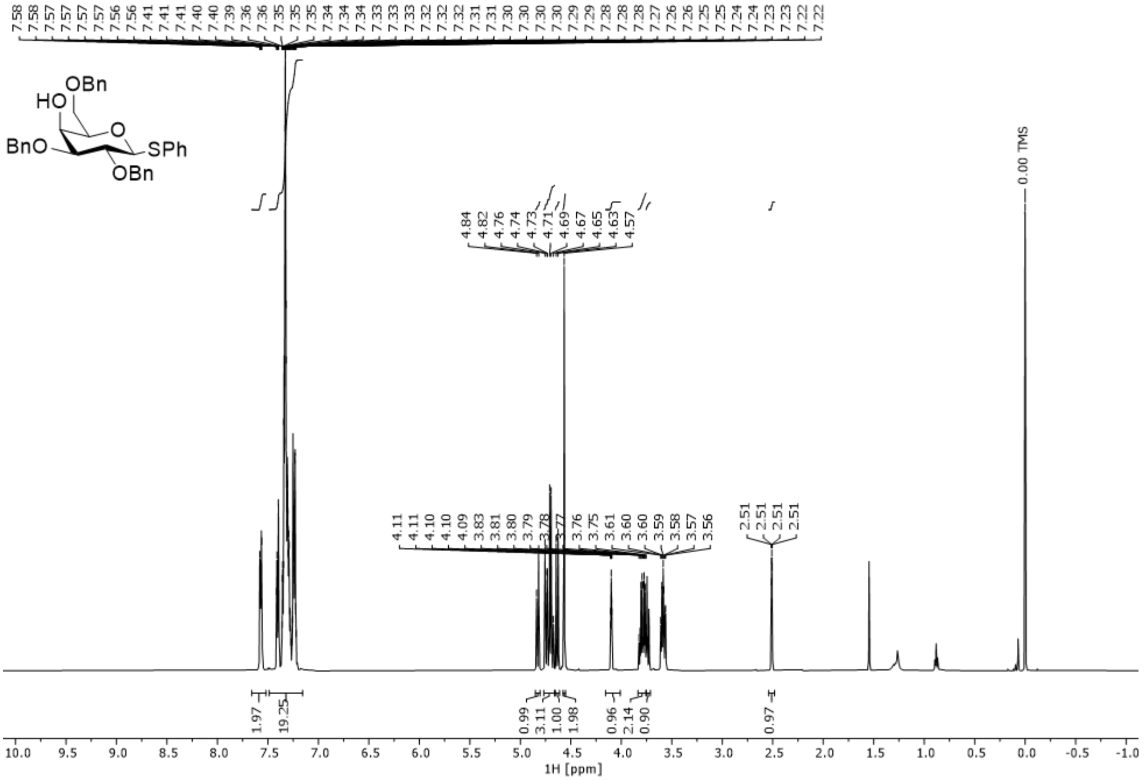

Figure S77: ^{1}H spectrum of the corresponding compound.

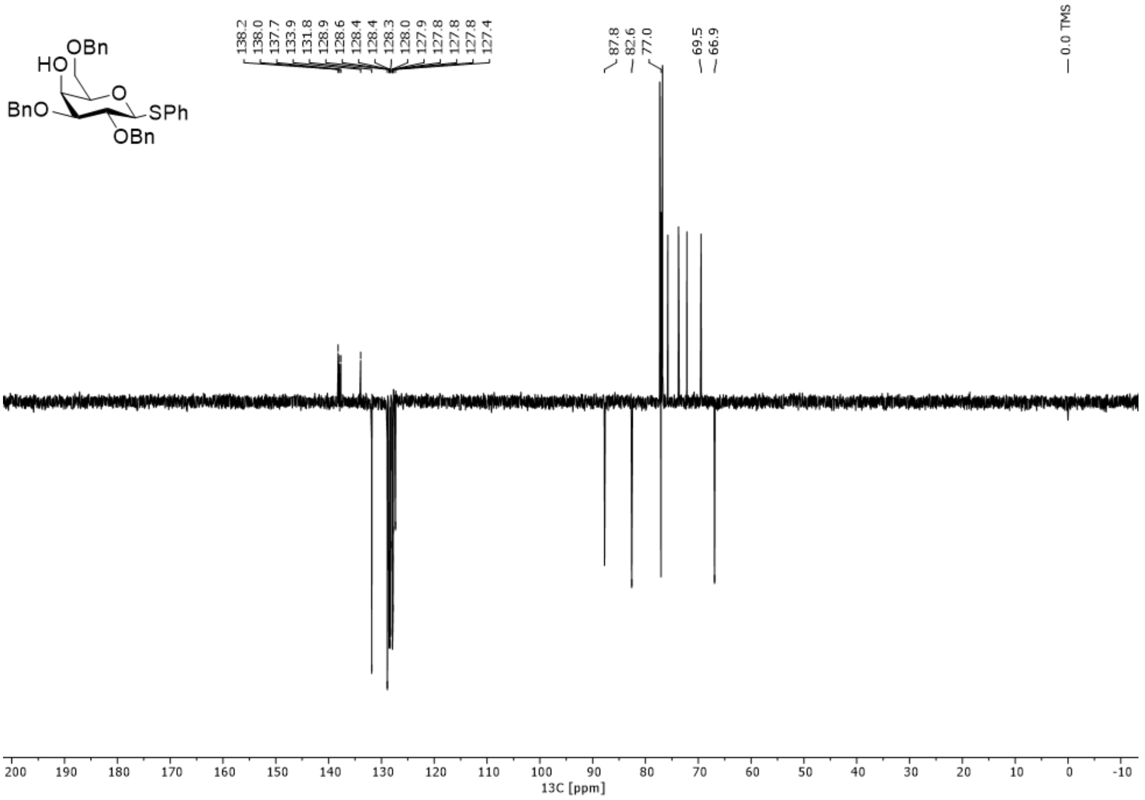

Figure S78: ^{13}C spectrum of the corresponding compound.

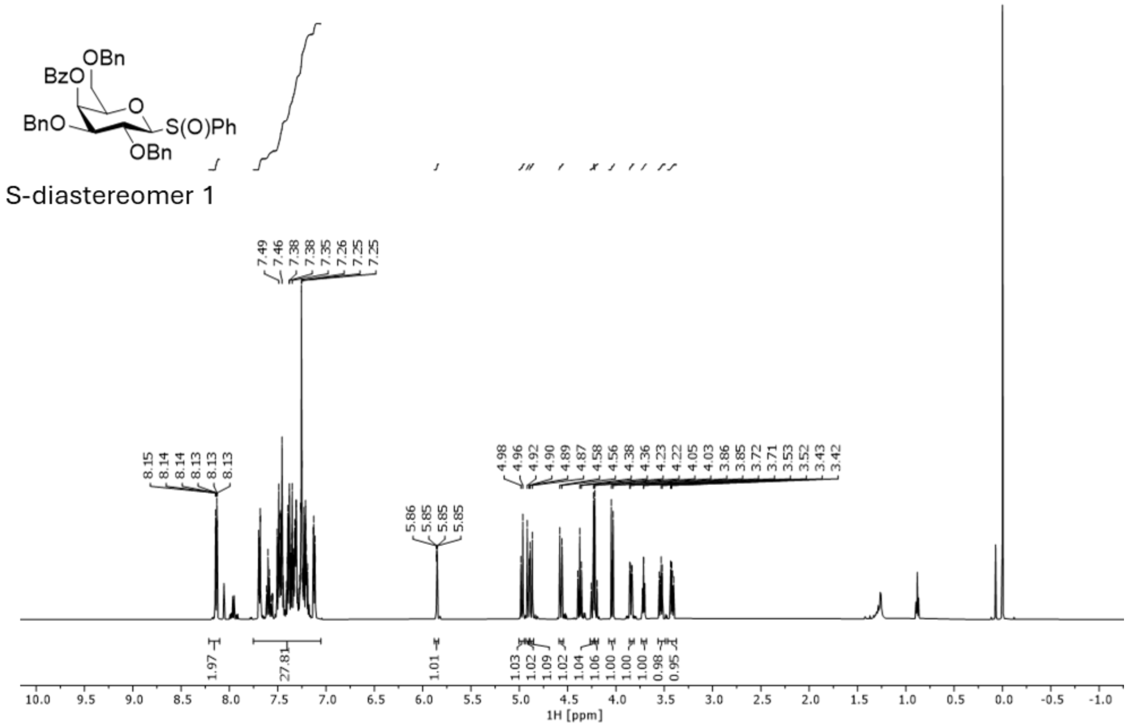

Figure S79: ^{1}H spectrum of the corresponding compound.

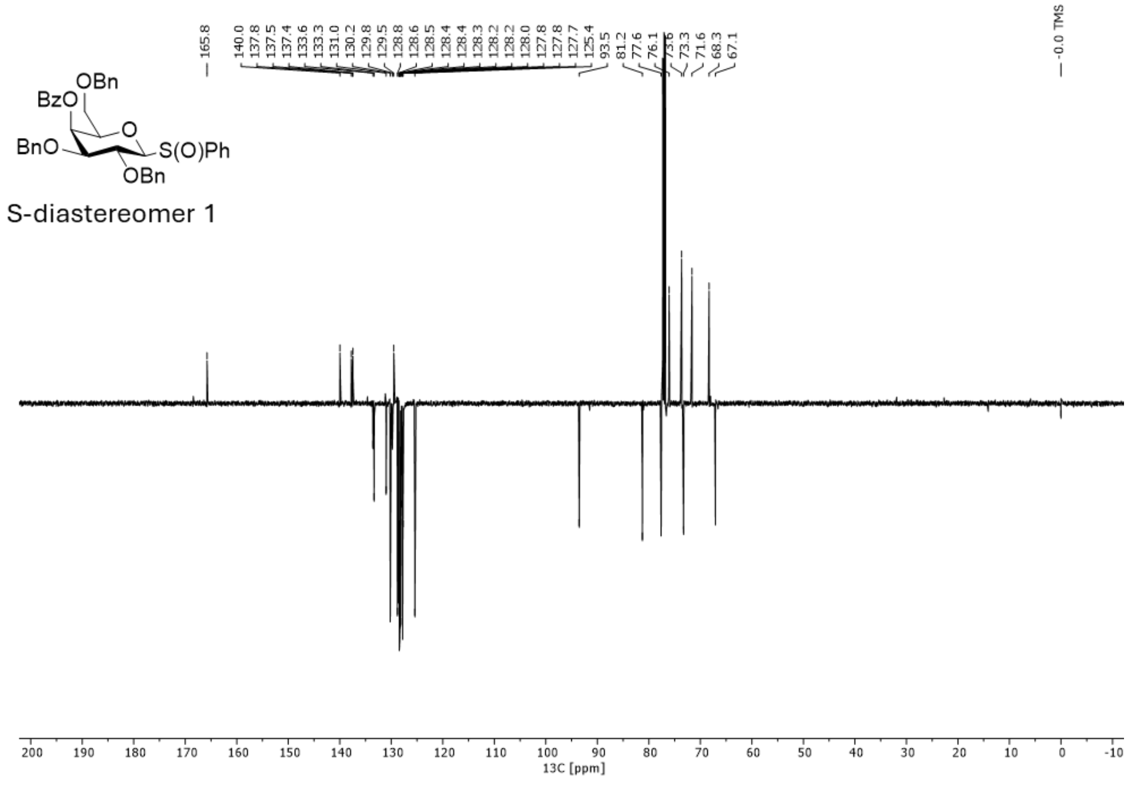

Figure S80: ^{13}C spectrum of the corresponding compound.

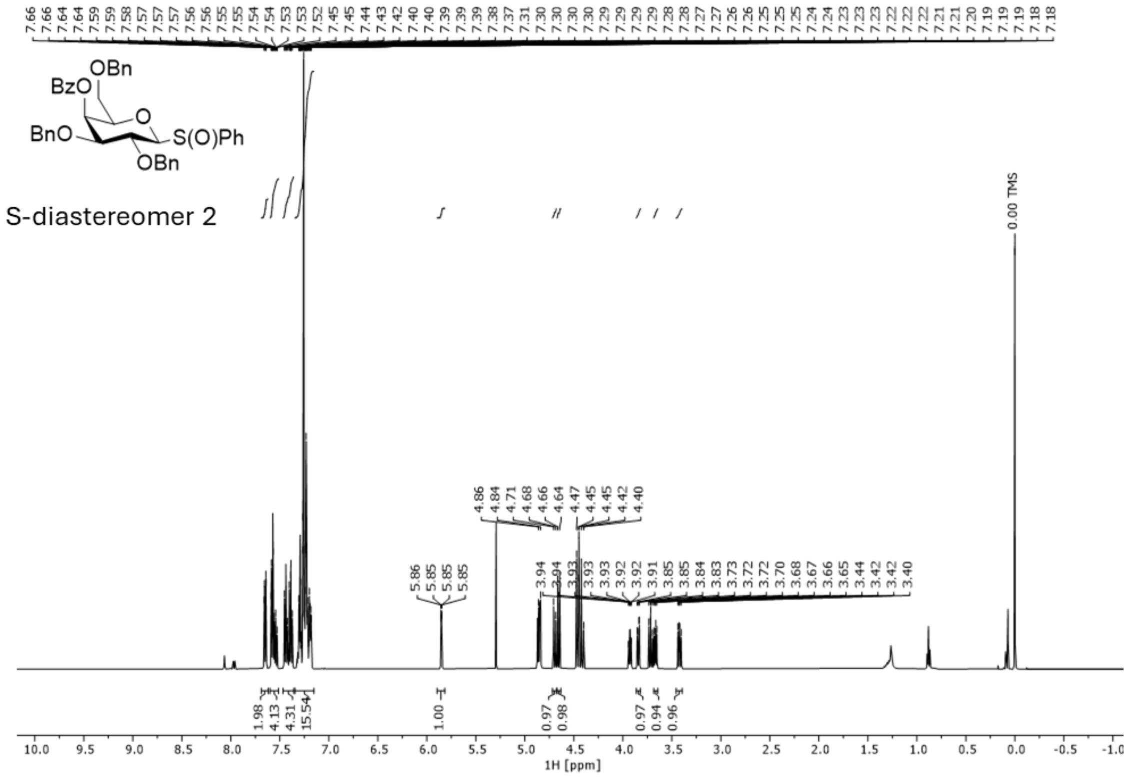

Figure S81: ^{1}H spectrum of the corresponding compound.

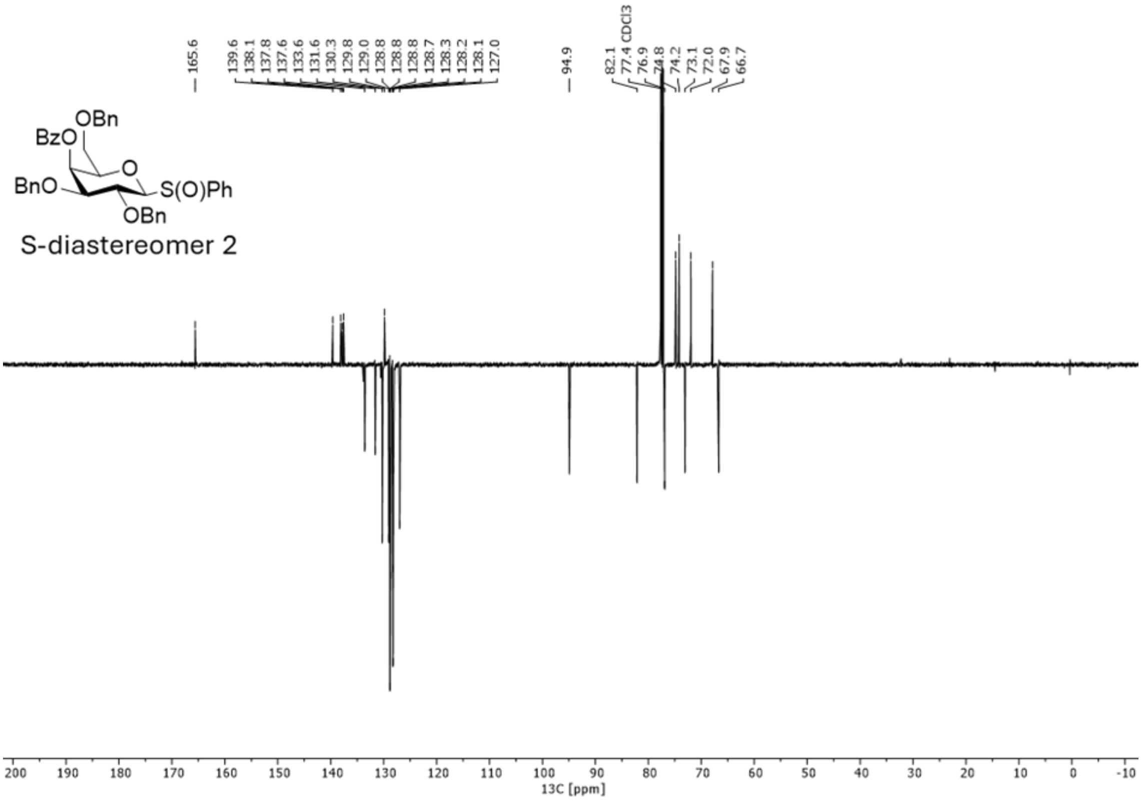

Figure S82: ^{13}C spectrum of the corresponding compound.

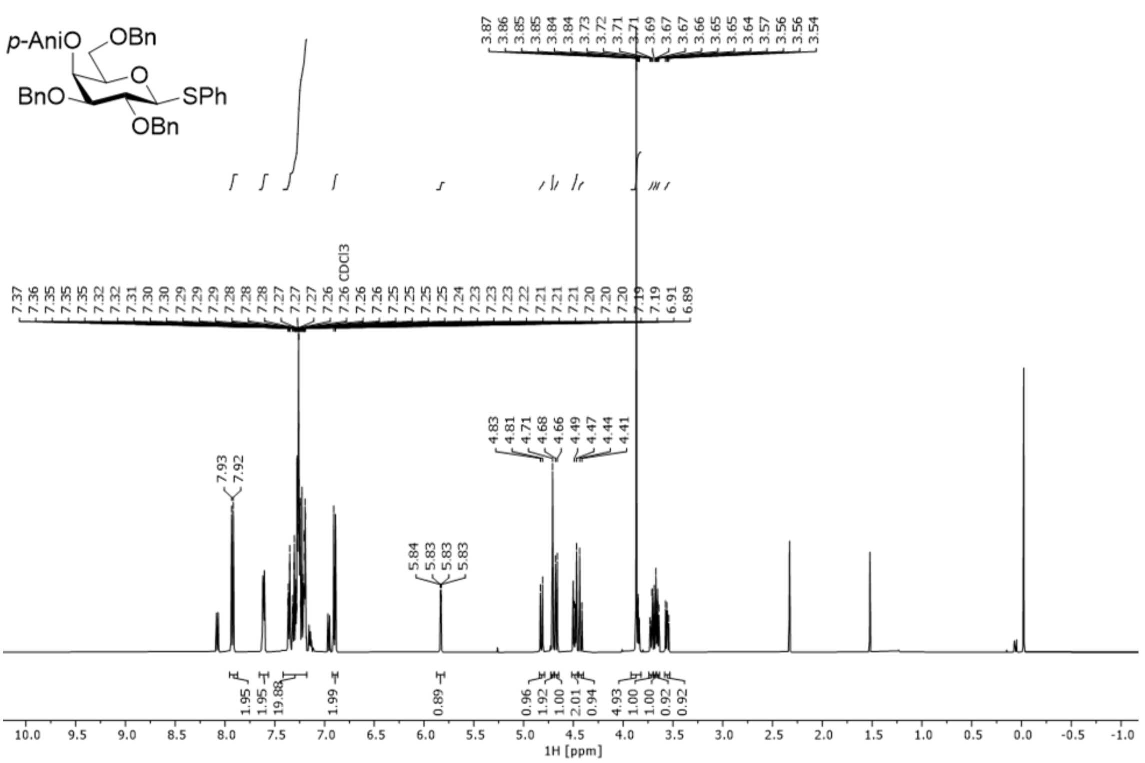

Figure S83: ^{1}H spectrum of the corresponding compound.

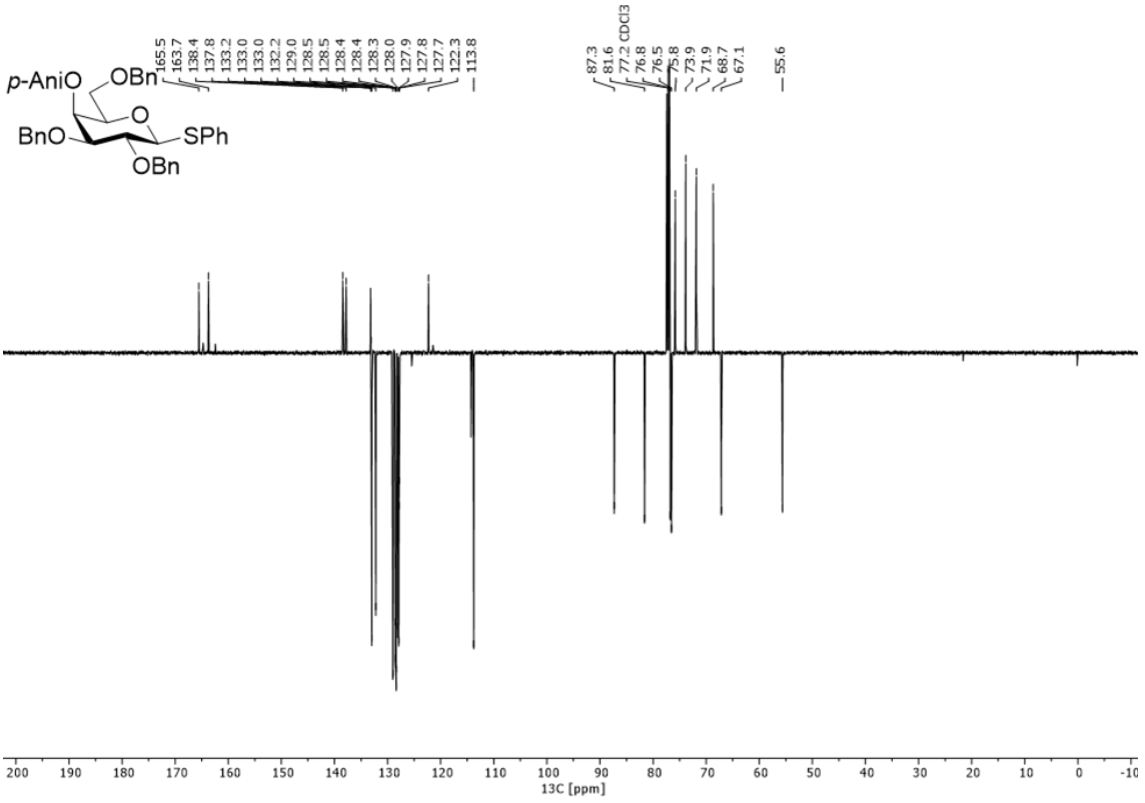

Figure S84: ^{13}C spectrum of the corresponding compound.

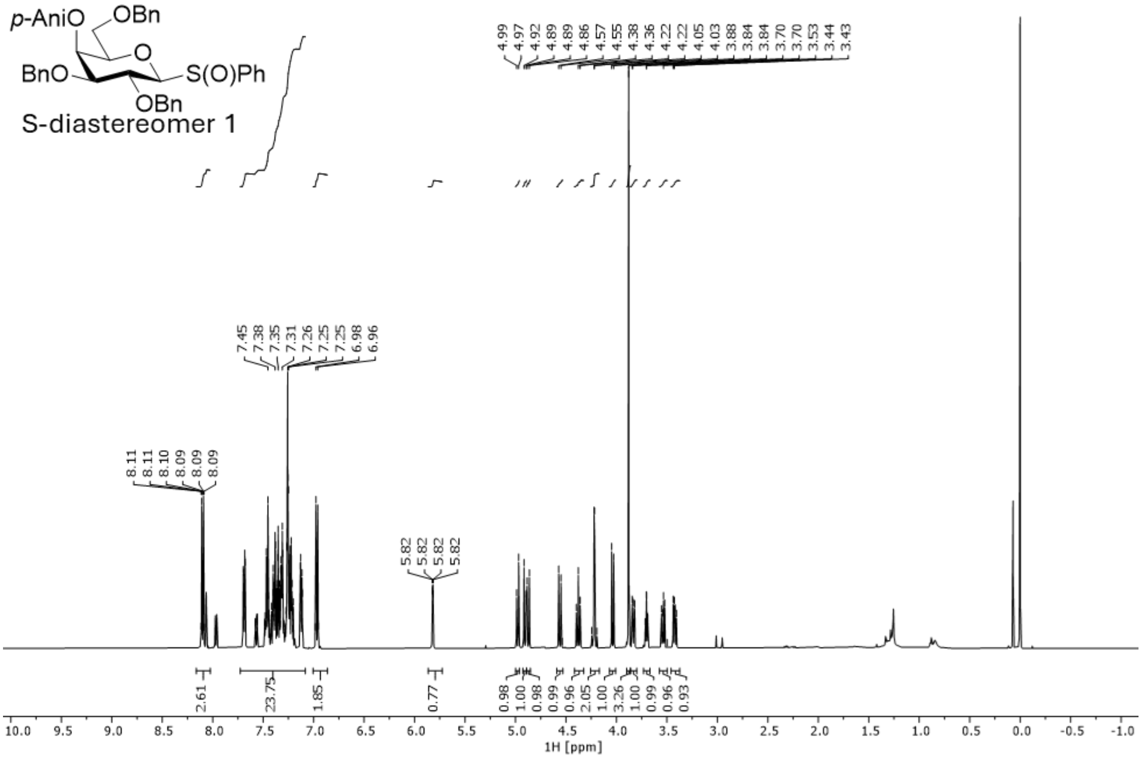

Figure S85: ^{1}H spectrum of the corresponding compound.

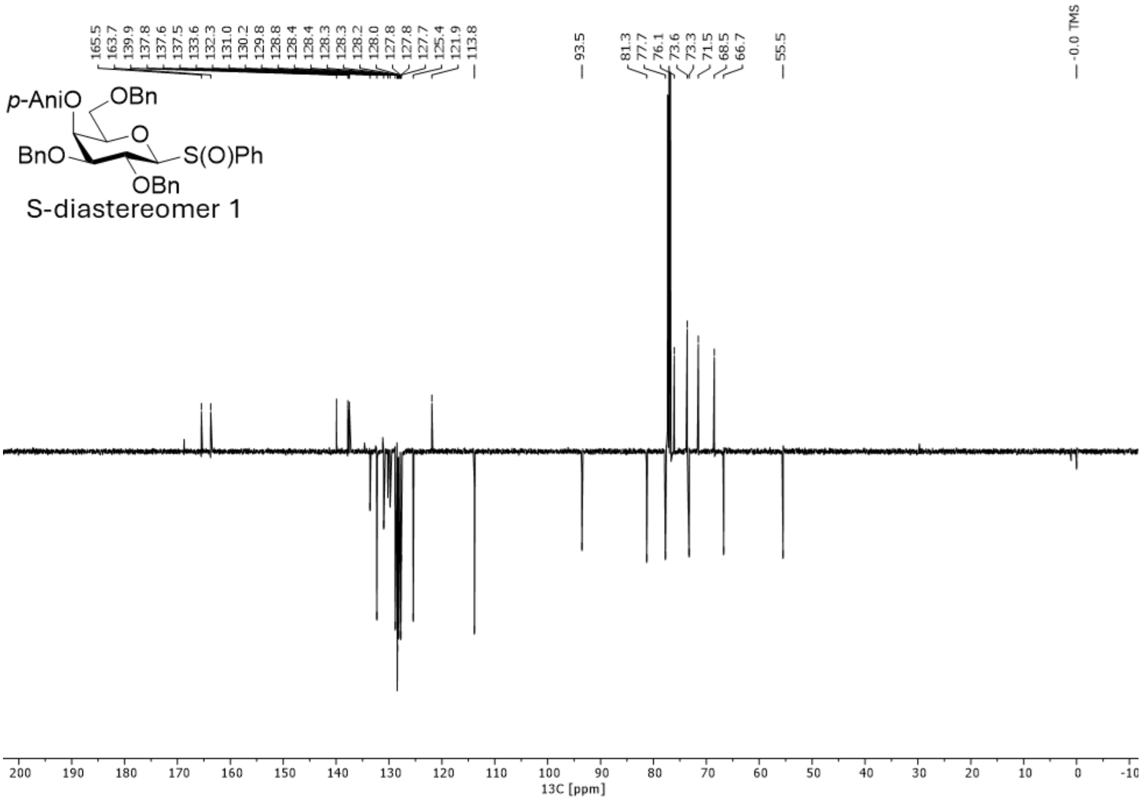

Figure S86: ^{13}C spectrum of the corresponding compound.

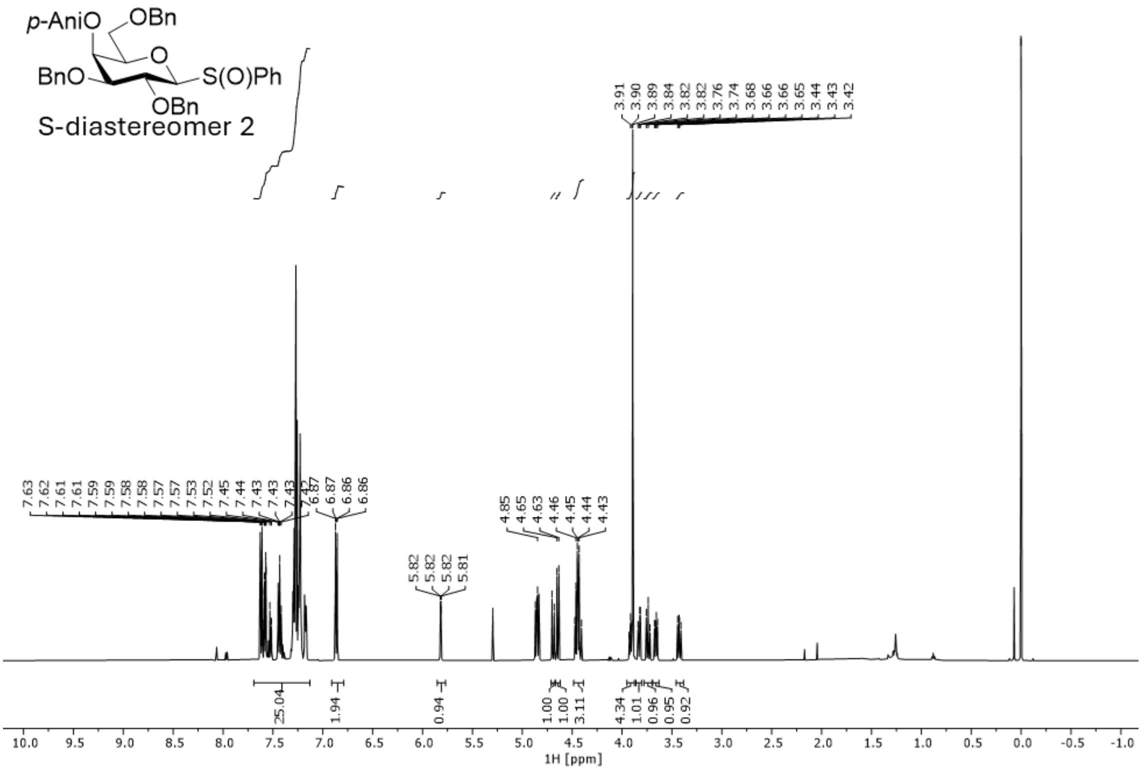

Figure S87: ^{1}H spectrum of the corresponding compound.

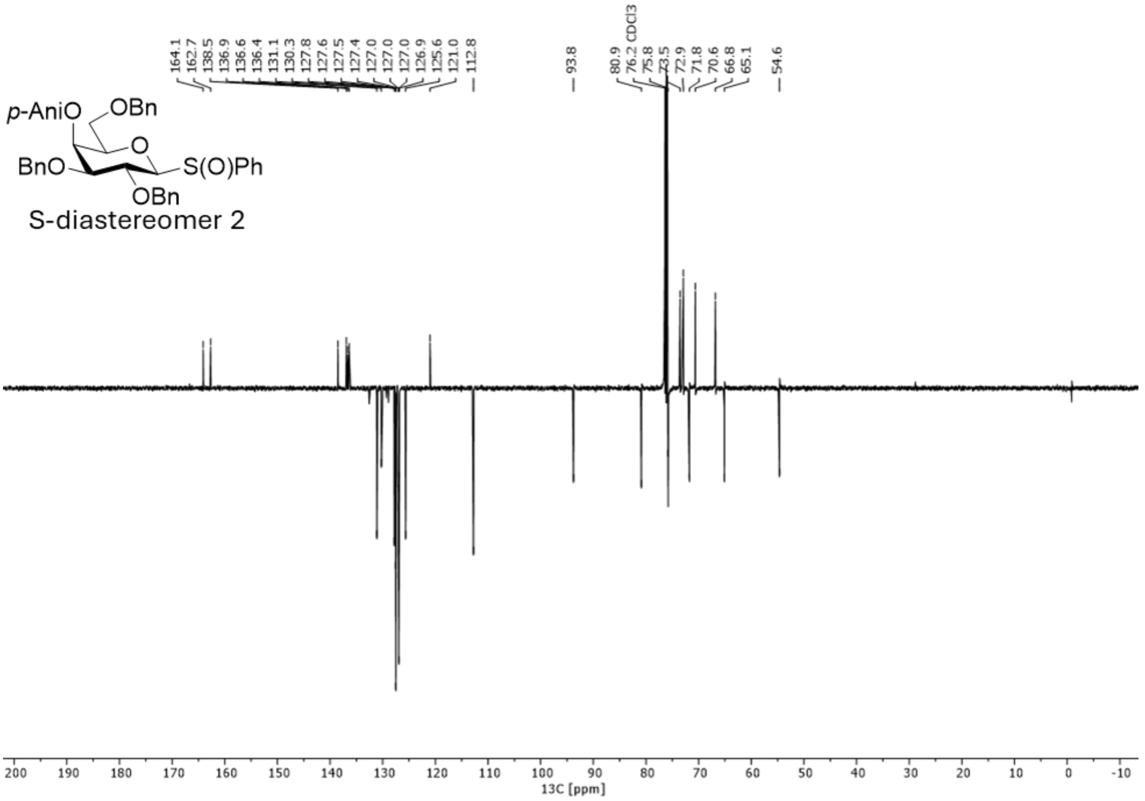

Figure S88: ^{13}C spectrum of the corresponding compound.

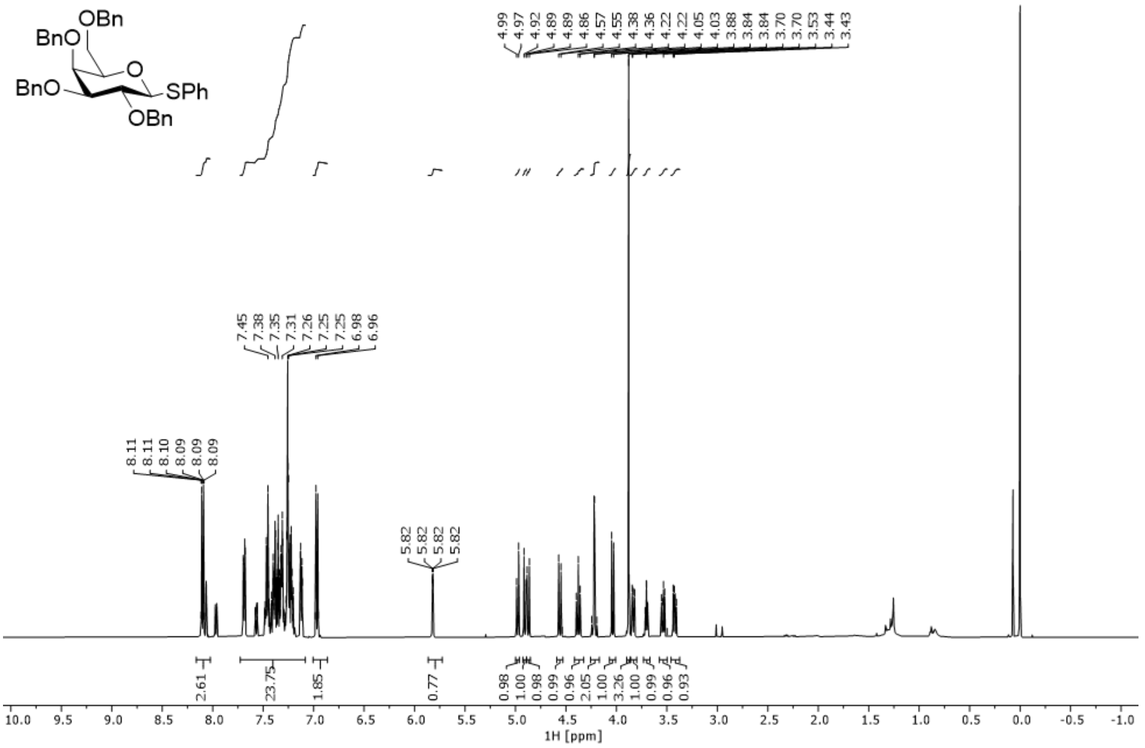

Figure S89: ^{1}H spectrum of the corresponding compound.

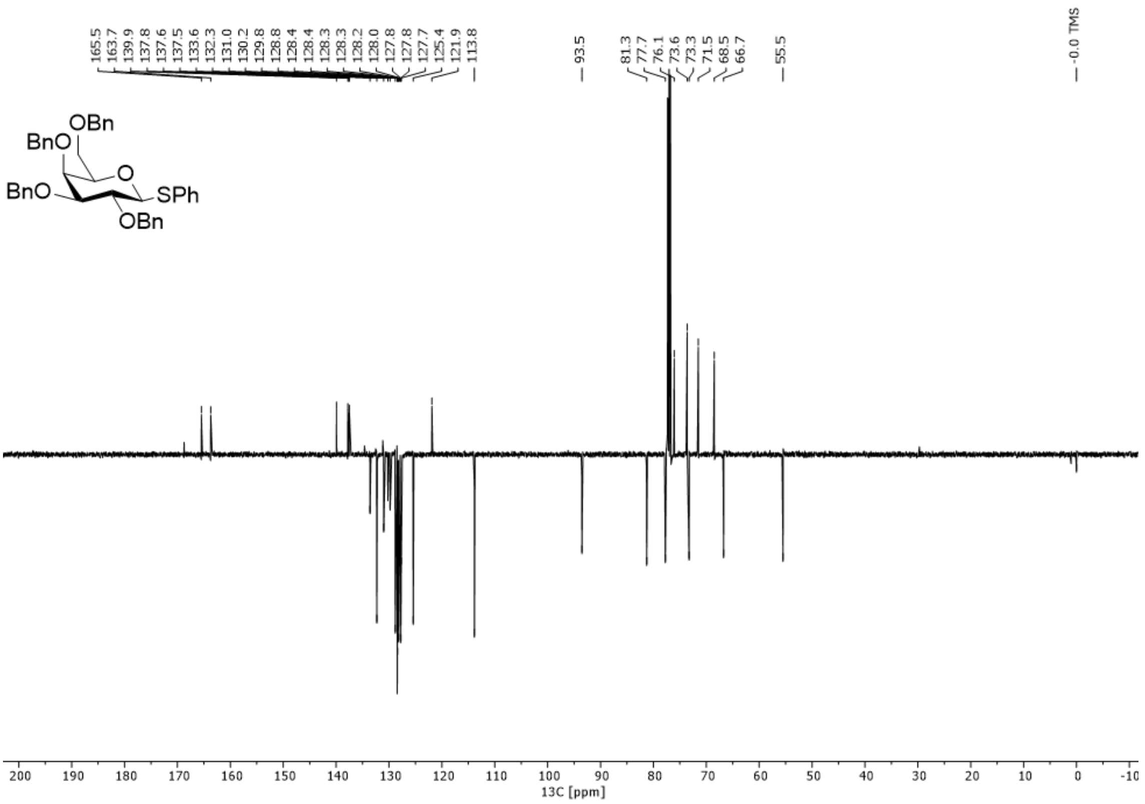

Figure S90: ^{13}C spectrum of the corresponding compound.

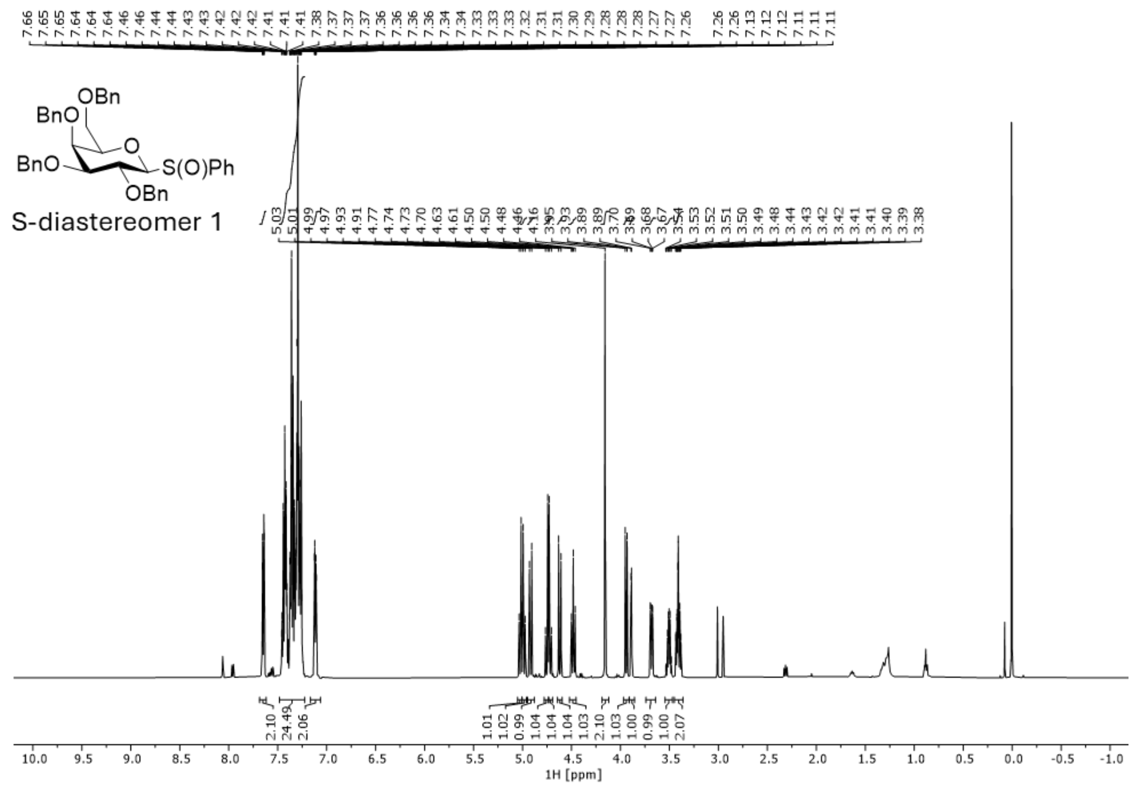

Figure S91: ^{1}H spectrum of the corresponding compound.

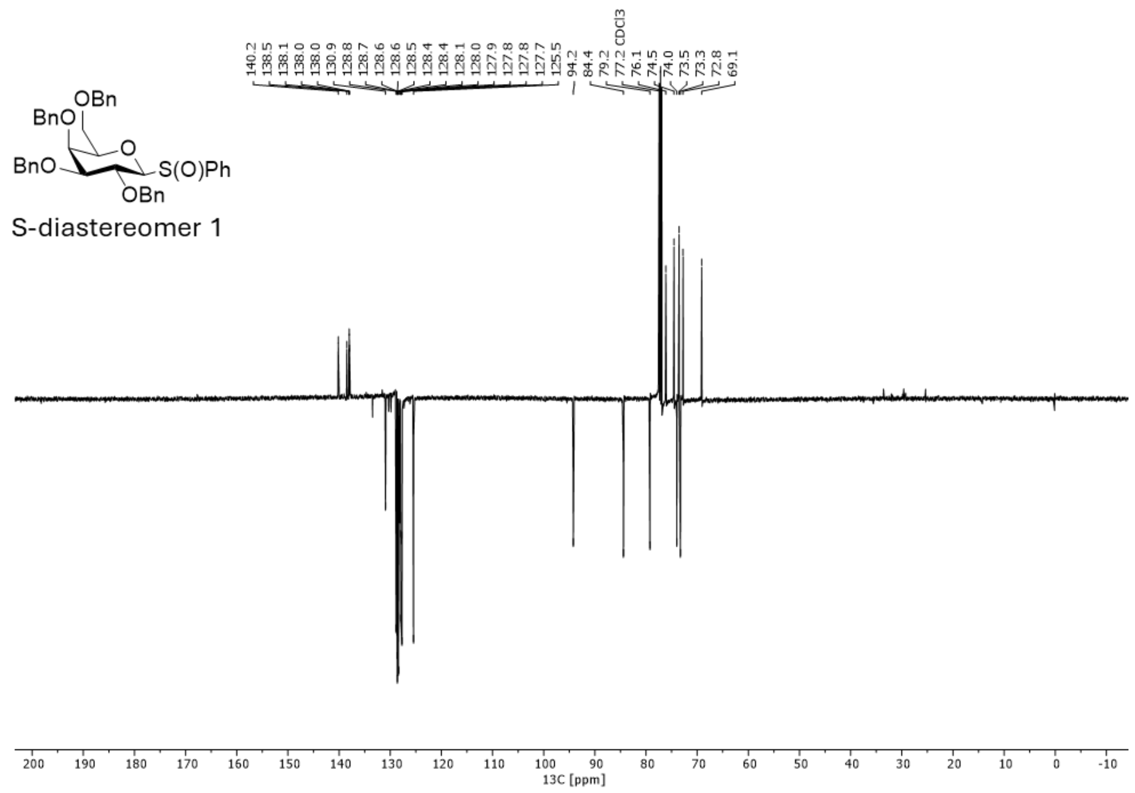

Figure S92: ^{13}C spectrum of the corresponding compound.

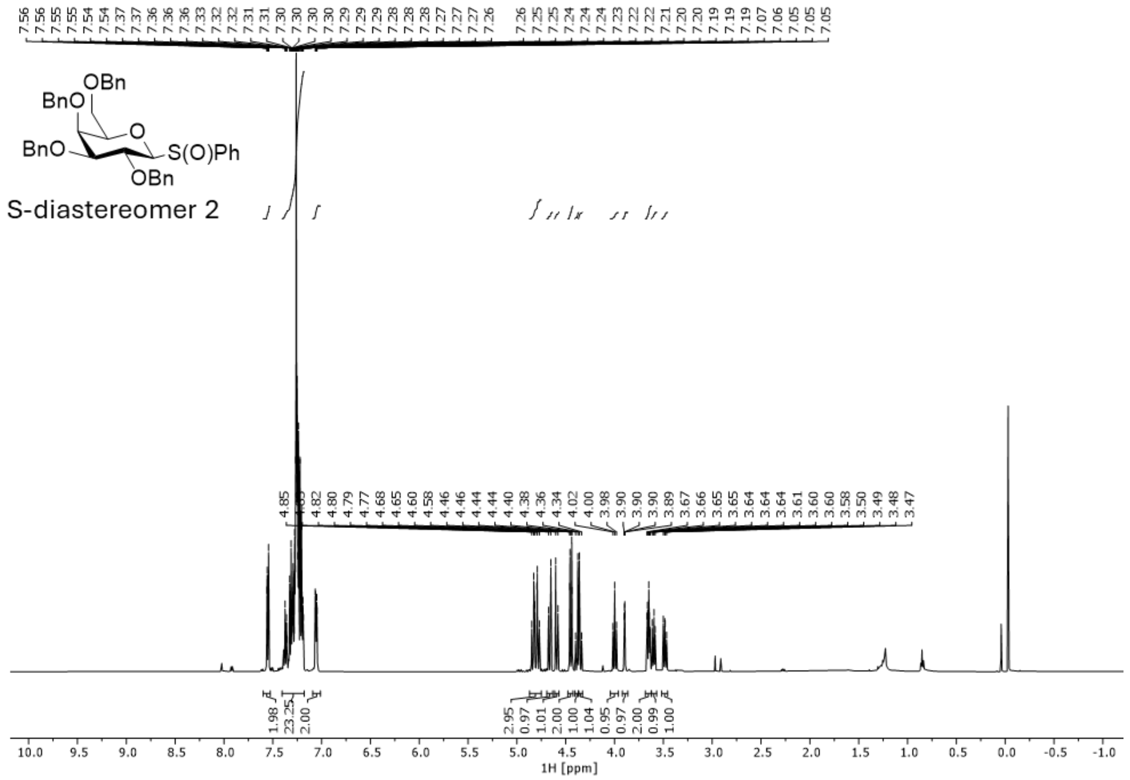

Figure S93: ^{1}H spectrum of the corresponding compound.

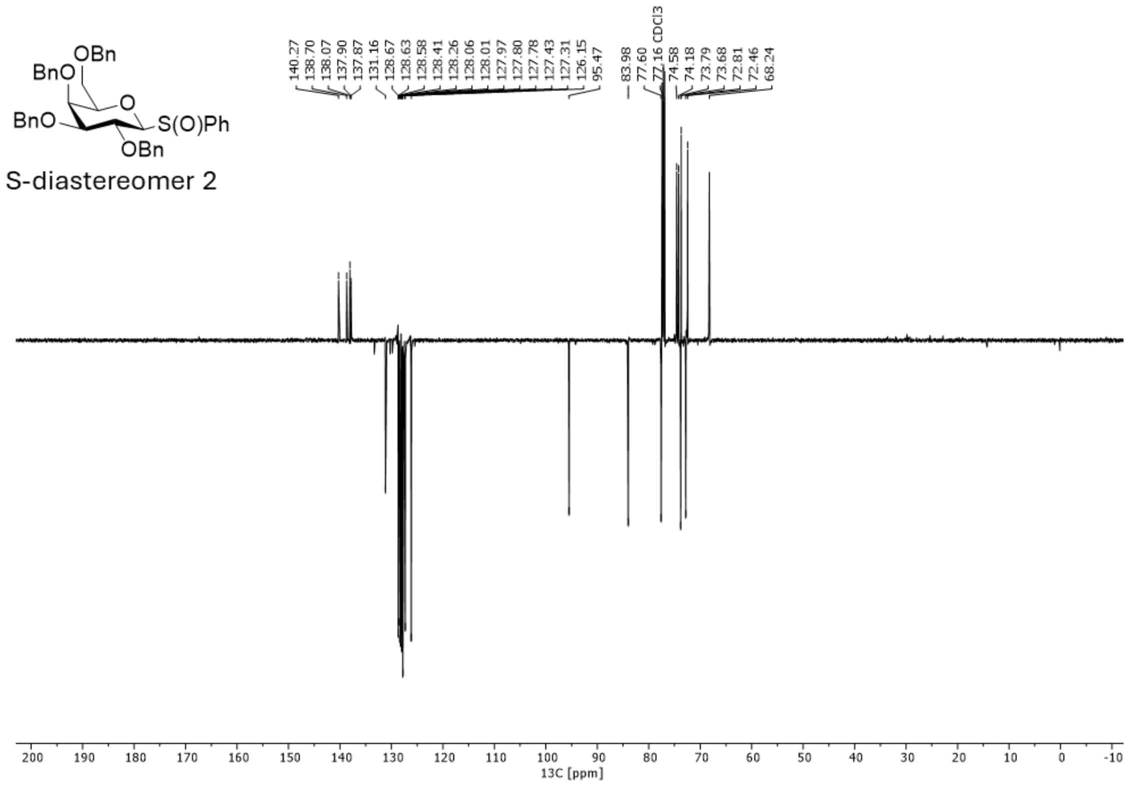

Figure S94: ^{13}C spectrum of the corresponding compound.

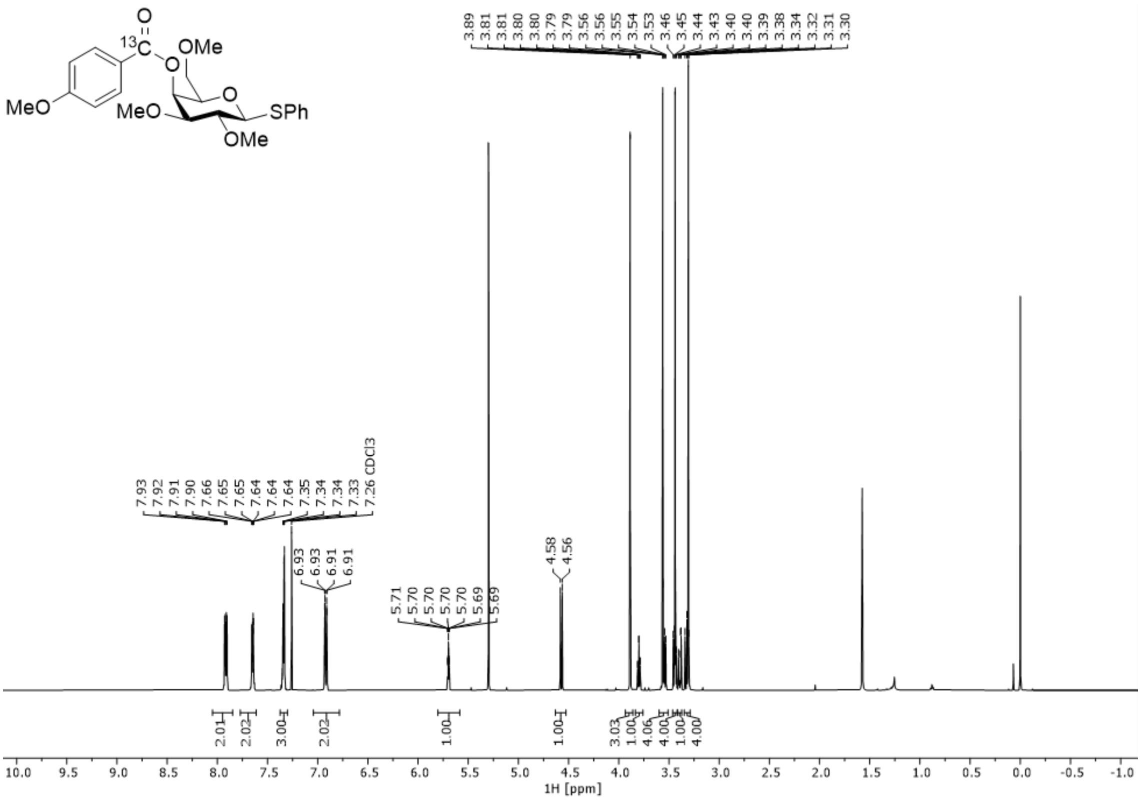

Figure S95: ^{1}H spectrum of the corresponding compound.

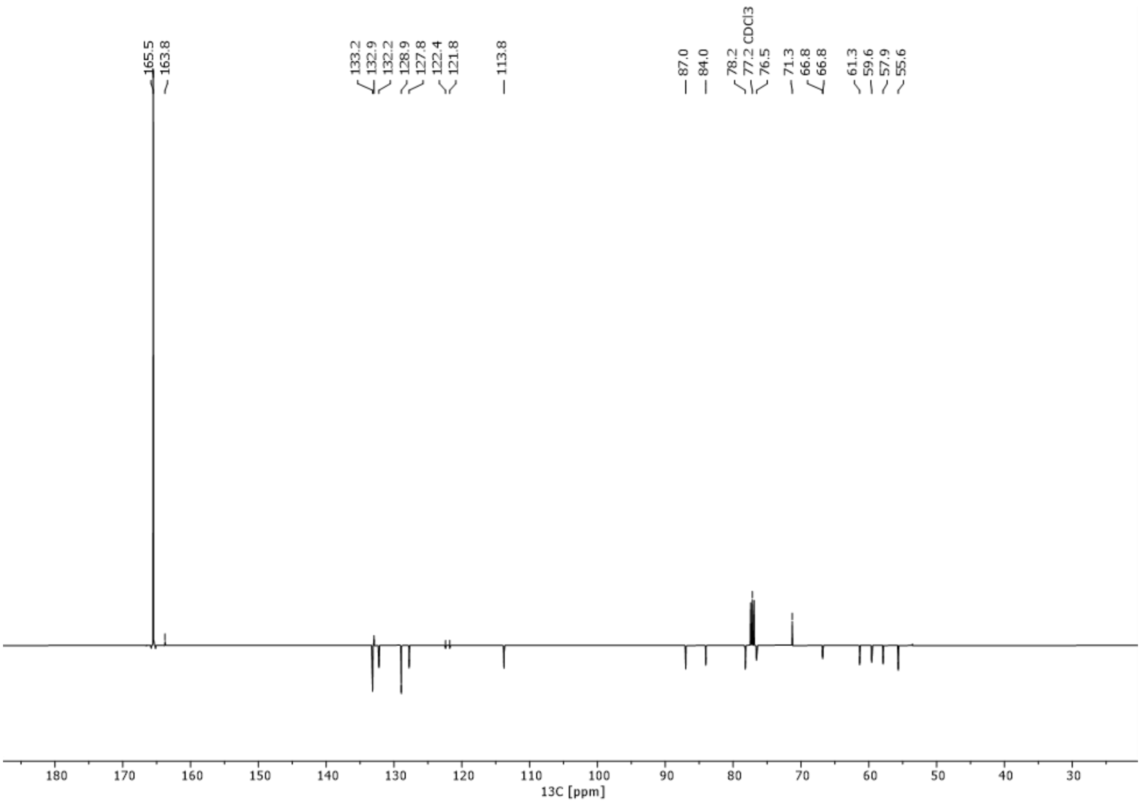

Figure S96: ^{13}C spectrum of the corresponding compound.
